# Impacts of coniferous bark-derived organic soil amendments on microbial communities in arable soil – a microcosm study

**DOI:** 10.1093/femsec/fiad012

**Published:** 2023-02-01

**Authors:** Krista Peltoniemi, Sannakajsa Velmala, Hannu Fritze, Tuula Jyske, Saija Rasi, Taina Pennanen

**Affiliations:** Natural Resources, Natural Resources Institute Finland (Luke), Latokartanonkaari 9, FI-00720 Helsinki, Finland; Natural Resources, Natural Resources Institute Finland (Luke), Latokartanonkaari 9, FI-00720 Helsinki, Finland; Natural Resources, Natural Resources Institute Finland (Luke), Latokartanonkaari 9, FI-00720 Helsinki, Finland; Production Systems, Natural Resources Institute Finland (Luke), Viikinkaari 9, FI-00720 Helsinki, Finland; Production Systems, Natural Resources Institute Finland (Luke), Survontie 9, FI-40500 Jyväskylä, Finland; Natural Resources, Natural Resources Institute Finland (Luke), Latokartanonkaari 9, FI-00720 Helsinki, Finland

**Keywords:** arable soil, bacteria, coniferous bark, forestry by-products, fungi, microbial community, organic amendments, side-streams

## Abstract

A decline in the carbon content of agricultural soils has been reported globally. Amendments of forest industry side-streams might counteract this. We tested the effects of industrial conifer bark and its cascade process materials on the soil microbiome under barley (*Hordeum vulgare* L.) in clay and silt soil microcosms for 10 months, simulating the seasonal temperature changes of the boreal region. Microbial gene copy numbers were higher in clay soils than in silt. All amendments except unextracted bark increased bacterial gene copies in both soils. In turn, all other amendments, but not unextracted bark from an anaerobic digestion process, increased fungal gene copy numbers in silt soil. In clay soil, fungal increase occurred only with unextracted bark and hot water extracted bark. Soil, amendment type and simulated season affected both the bacterial and fungal community composition. Amendments increased bacteria originating from the anaerobic digestion process, as well as dinitrogen fixers and decomposers of plant cells. In turn, unextracted and hot water extracted bark determined the fungal community composition in silt. As fungal abundance increase and community diversification are related to soil carbon acquisition, bark-based amendments to soils can thus contribute to sustainable agriculture.

## Teaser

Bark-derived organic soil amendments affect fungal and bacterial gene copy numbers, their community composition and have the potential to increase microbes, promoting soil and plant health processes.

## Introduction

Deforestation and conversion of land to agriculture depletes one third of the soil carbon (C) pool during the first 10 years and thereafter continues, for example, in Finland at a rate of 0.4% yr^−1^ (Heikkinen et al. 2022), which is in line with estimations of LUCAS surveys conducted from European arable soils (Panagos et al. 2020). Long-term soil C loss also leads to lower crop yields that can be converted if soil C sequestration is increased (Lal 2004). Soil microbes, particularly fungi, are connected to increased C sequestration potential during the restoration of agricultural soils (Morriën et al. 2017, Yang et al. 2022). Indeed, high fungal abundance is reported to result in microbial-derived soil organic matter (SOM) accumulation (Godbold et al. 2006, Kallenbach et al. 2016). Also, organic interactions between the necromass of Gram-negative bacteria and soil yeasts contributed to retention of necromass-C and N in soils (Buckeridge et al. 2020). Furthermore, stable soil organic C formation is linked to soil microbe activity according to the Microbial Carbon Pump hypothesis (Liang et al. 2017), which counteracts the traditional plant litter-centered perspective.

Most arable soils were once forests forming a "wood-wide-web" with fungi as the key soil organisms bridging trees together (Helgason et al. 1998). Fungi affect major ecological processes such as nutrient cycles, good soil structure and disease control, likely preventing further degradation and C loss of arable soils. Thus, management practices restoring fungi are important (Frąc et al. 2018, Hannula and Morriën 2022). There are a few indications of potential positive effects after the application of forestry- or wood-based amendments on soil conditions and soil organisms. For instance, processed pulp mill and fiber sludges from paper mills, used as organic amendments, protected soil from erosion and had a promising positive impact on soil microbial communities (Rasa et al. 2021). Furthermore, wood-derived organic amendments favored saprotrophs over potential plant pathogenic fungi (Clocchiatti et al. 2021a), and forest litter amendments decreased *Fusarium* infections of wheat, probably via forest soil microbes (Ridout and Newcombe 2016). Wood sawdust stimulated the activity of various soil fungi but also the abundance of potentially beneficial rhizosphere bacteria (Clocchiatti et al. 2021b). Another recent study showed that composted pulp mill sludge and fiber sludge induced a very different soil microbial community compared with addition of chopped clover roots (Heikkinen et al. 2021). The study by Heikkinen et al. (2021) showed that wood-derived material has the potential to trigger the fungal community to diversify, which can be reflected in soil C acquisition in agricultural soil, indicating the power of soil amendments to induce community shifts lasting for several years.

Forestry-derived side-streams with potential as amendments to promote soil health can be obtained from wood chips and bark from sawmills, residuals of wood and bark from bioethanol and biogas plants and side-streams from paper pulping. The Finnish Forest industry generated almost 7 million m^3^ of bark as a by-product in 2019, which is usually combusted to generate energy in the form of steam, heat and electricity (Rasi et al. 2019). Forest industry side-streams are an important asset for a sustainable and circular bioeconomy as, instead of burning for energy, they can be used for improving soil structure and health. Although there is substantial potential to use forestry-derived bark side-streams, their suitability as soil amendments is, yet, largely undetermined.

Fresh bark is rich in polyphenolic compounds such as stilbenes and tannins, which have antimicrobial properties preventing microbial growth and functions (Jyske et al. 2022) and might need processing before applied as soil amendment. These naturally bioactive compounds can be isolated from the bark biomass by methods of green chemistry, such as hot water extraction, and processed further into added-value use as functional bioproducts and chemicals (Raitanen et al. 2020, Välimaa et al. 2020, Pap et al. 2021, Granato et al. 2022). The extracted residual bark can be further utilized in follow-up bioprocessing, such as anaerobic digestion. Thus, by cascade processing combining different unit operations, such as hot water extraction and further anaerobic digestion (Rasi et al. 2019), the bark biomass can be fully utilized. Digestates from conventional biogas processes are commonly used as soil amendments and they have proven to improve soil properties and to increase soil carbon content (Jurgutis et al. 2021). However, the effect of anaerobically treated bark-derived amendments is not well studied.

To estimate the impact of bark-derived organic amendments on the abundance and composition of soil microbes (bacteria and fungi), we established a laboratory-scale microcosm experiment to simulate the possible effects throughout an entire growing season, from sowing to harvest. We used barley: a common cereal cash crop plant typically grown in boreal fields. Bark-derived organic amendment materials of two boreal coniferous tree species, namely Scots pine (*Pinus sylvestris* L.) and Norway spruce (*Picea abies* L. Karst), were used. Furthermore, we investigated the effect of digestates resulting from anaerobic biogas processes with rarely used bark-derived organic fractions. Based on the previous findings (Heikkinen et al. 2021), we expected that the addition of bark-derived organic amendments to agricultural soils might serve to increase soil fungal biomass and change the community structure, harboring species connected to soil C sequestration. Thus, we investigated the impact of different coniferous bark-derived organic amendments on (i) microbial abundance (gene copy amounts) and (ii) community composition (amplicon sequencing) in silt and clay arable soils. Our hypotheses were that (1) the unextracted industrial bark and processed bark-derived organic amendments affect bacterial and fungal communities differently, and (2) the soil type (silt vs. clay) determines microbial response to amendments.

## Materials and methods

### Microcosm laboratory experiment

For the microcosms, about 20 liters of arable soil were taken with a clean shovel from the tilled (10 cm) surface of two separate agricultural field sites without plant cover in May 2018. These two sites represented a silt soil (pH 6.9, 7% organic and 72.7% dry matter) from Mikkeli (southeastern Finland 61.68°N, 27.22°E; Pakarinen et al. 2021) and a clay soil (pH 6.5, 2% organic and 76.8% dry matter) from Jokioinen (southwestern Finland 60.80 N, 23.46 E; Rasa et al. 2021). The soils were kept outdoors for up to 48 h before taking them to the laboratory where they were stored at 4°C for a few days prior to starting the microcosm experiment. The microcosm soil pH was determined in distilled water (1 : 3.5, vol/vol), and total C and N were measured from sieved and air-dried samples using a CN analyzer (Leco-TruMac, Leco Corp., MI, USA) (Table 1).

**Table 1.** Mean soil pH (n = 2), total carbon (Ctot) and nitrogen (Ntot) contents (% dry matter) from control soils and organic amendments (n = 1) in the microcosms. Abbreviations: C, control soils without amendments; B, untreated conifer bark; BH, hot water extracted bark: BA, digestate containing untreated bark from an anaerobic digestion process; BHA, digestate containing hot water extracted bark from an anaerobic digestion process. Data from amendments deriving from pine and spruce conifer barks are combined.

Microcosm	pH	Ctot	Ntot
C (silt)	6.9	6	0.3
C (clay)	6.5	3	0.2
B	3.5	49	0.3
BH	3.9	48	0.3
BA	8.6	36	2.7
BHA	8.6	35	2.7

The microcosms comprised four replicate aerated plastic containers (107×94×65 mm, sterivent low container, Duchefa Biochemie, Haarlem, The Netherlands) per treatment (n = 72). Controls had soil only and, in the amended treatments, soil was mixed with, altogether, eight different types of bark-derived materials of both pine and spruce tree species: (1) industrial conifer bark without extraction treatment (B), (2) industrial conifer bark after hot water extraction treatment (BH), (3) digestate containing industrial, unextracted bark after an anaerobic digestion process (BA) and (4) digestate containing industrial, hot water extracted bark after an anaerobic digestion process (BHA). Industrial bark of Norway spruce (*Picea abies* (L.) Karst) and Scots pine (*Pinus sylvestris* L.) trees was obtained from a sawmill (Veljekset Vaara Oy) in Tervola, north-western Finland. Trees were felled in late 2017 and peeled in the sawmill in early January 2018. The fresh bark was collected, transported to the laboratory and stored in the dark at −20°C until further processing. Prior to any processing, bark was milled into 5 cm chips with a cutting mill shredder (Fritsch, Pulverizette, Germany). The hot water extraction was done at 75°C for 1 h using 3 L flow-through extraction equipment (see Väisänen et al. 2019). Anaerobic digestion processing for unextracted and hot water extracted bark was done as a biochemical methane potential (BMP) experiment in mesophilic conditions (37°C). Inoculum for the BMP process was from a farm-scale biogas plant treating cattle slurry, and the volatile solids ratio of 0.5 for substrate/inoculum (VS: VS) was used (Rasi et al. 2019). Dry weights of the materials were pine B 42%, spruce B 44%: BH 35% and all the BA and BHA amendments 6%; 21 g fresh weight (fw) of B, 28 g (fw) of BH and 50 g (fw) of BA and BHA amendments were added to the microcosms filled with 380 g of fresh soil. The C additions to the microcosms with the amendments corresponded to 11 500 kg ha^−1^ (3.8 g C per microcosm) for B and BH and 3000 kg ha^−1^ (1 g C per microcosm) for BA and BHA treatments, if performed at field level. The amounts of organic materials in microcosms were adjusted to correspond approximately to the levels of C in fields applied with organic amendments (see, for example, Rasa et al. 2021). We estimated that, in the field, the amendments are mixed into the uppermost 15 cm ploughing depth. As the thickness of soil layer in the microcosms was only 5 cm, we adjusted the amounts of amendments to corresponding proportions by dividing by three.

The microcosm study was conducted in climate chambers (Binder KBW 720/400, WTB Binder Labortechnik GmbH, Tuttlingen, Germany) simulating soil temperature conditions occurring over 1.5 years in a boreal agricultural field with barley as a crop plant in southern Finland. Barley was chosen because it is the most common crop grown in Finland. The temperature cycling was adjusted so that the change in soil temperature was two times faster than in the field, and thereby we achieved a temperature cycle of over 1.5 years and two growing seasons in 10 months. Simulation of the annual changes in soil temperature was important because fluctuating temperature is relevant to activation of environmental microbes (Korkama-Rajala et al. 2008). Moisture in the microcosms was followed by weighing and water was added when needed. After 7 months of incubation, barley seeds were sown in the microcosms, which were transferred to 18 h light/6 h darkness at 20ºC for 1.5 months (Fig. S1). After 2 weeks the lids were opened because the barley grew taller than the containers. Full daylight tubes were used and the photosynthetically active radiation in the chambers, measured with an Apogee MQ-200 quantum meter (Apogee Instruments, Inc., Logan, Utah, USA), was 170 µmol m^−2^ s^−1^. Barley shoots were harvested by cutting the green parts after 1.5 months growth and biomass was determined by weighing the mass after drying at 50°C for 48 h. Dried shoots were returned to the respective microcosms to let them decompose there.

The experiment was sampled four times: we performed the first sampling during the simulated early winter (4-month incubation); the second during the simulated spring before sowing (6-month incubation); the third during the post-harvest of barley in simulated autumn (8-month incubation); and the fourth from the bare field before simulated winter (10-month incubation) (Supplementary Fig. S1). Sampling was performed by combing small portions of soil, taken by spoon from over the microcosm, into one bulk sample per microcosm.

### DNA extraction and sequencing

At each of the four sampling times, soil sub-samples (n = 288 total amount of samples; n = 72 for each sampling) were taken from each microcosm with a sterile spatula and frozen at -20°C for DNA extraction. DNA was extracted using a NucleoSpin Soil kit (Macherey Nagel, Düren, Germany), according to the protocol of the manufacturer. DNA concentration and purity were determined with a NanoDrop Lite spectrophotometer (Thermo Fisher Scientific, Waltham, MA, USA). Quantitative PCR (qPCR) for the bacterial 16S rRNA genes and partial fungal ITS2 region from the DNA obtained from all four samplings was conducted as described by Peltoniemi et al. (2015). DNA from the second and third samplings, respectively, originating from three out of four replicate microcosms (n = 54 for each sampling), was used for sequencing at the Institute of Genomics of Tartu University, Estonia. For bacteria, the targeted V4 region of the 16S SSU rRNA, and for fungi the ITS2 region, were amplified in a two-step PCR. Bacterial and fungal PCR were performed using the 16S rRNA primers 515F and 806R (Caporaso et al. 2011, 2012) and the ITS primers ITS4 (White et al. 1990) and gITS7 (Ihrmark et al. 2012), respectively, with 8 bp dual index for 24 cycles. The final PCR fragments were run as paired-end 2×300 bp with the MiSeq platform (Illumina, San Diego, CA, USA) using a MiSeq v3 kit producing ∼20–25 M reads per flow cell.

### Processing of raw sequence data and bioinformatics

Sequence assembly, quality filtering, removal of chimeras, primer-dimers and primers from raw 16S and ITS2 sequence reads, along with clustering and taxonomical annotations, were conducted with a PipeCraft 1.0 pipeline (Anslan et al. 2017) as described by Soinne et al. (2020) with slight modifications. Briefly, in the second quality filtering, OTUs that had similarity below 90% for 16S rRNA data and 75% for fungal ITS2 data, query coverage below 70% and reads that were observed fewer than 10 times and OTUs with associations other than bacteria or fungi, were removed from the data. In addition, all mitochondrial and chloroplast matches were removed. Fungal ITS2 derived OTUs were combined according to species hypothesis (Kõljalg et al. 2020; Nilsson et al. 2019). The bacterial 16S rRNA raw data for the second and third samplings consisted of 1981 774 and 2074 812 reads clustering into 17 975 and 18 145 OTUs, respectively, and the respective values after trimming were 1909 593 and 1997 696 reads clustering into 7144 and 7213 OTUs. The fungal ITS2 raw data for the second and the third sampling consisted of 1483 987 and 1530 156 reads clustering into 3699 and 3023 OTUs, respectively, and the respective values after trimming were 1317 334 and 1321 392 clustering into 901 and 784 OTUs. Raw sequence data are deposited in the sequence read archive of the NCBI database under BioProject PRJNA607883 with the accession numbers SAMN30932759–SAMN30932812 for 16S rRNA and ITS2 data.

### Statistics

The total sample count for the experiment was 288 (including four different organic amendments from spruce and pine bark with four samplings in two different soil types, silt and clay, and four replicated control treatments for both silt and clay). All statistical analyses were conducted using R studio version 1.2.5001 and R version 3.6.0 or 3.6.1 (R Core Team 2021). To study the effects of organic amendments on the 16S rRNA gene and ITS-region copy numbers, a linear mixed effects model was fitted by maximum likelihood (lme function from the nlme package). Fungal and bacterial qPCR copy numbers were the response variable and organic amendment as the explanatory factor and tree species (pine vs. spruce) and sample id (microcosm nr) nested within the sampling timepoint as the random factor. The response variables were square root transformed prior to analysis to normalize the distributions. Dunnett's post-hoc comparisons between the control and the four amendments were done with *glht* from the *multcomp* package and Bonferroni correction of *P* values to adjust for multiple comparisons.

OTU data from the amplicon sequencing from the second and third datasets (n = 54 for each sampling) were combined and normalized using the geometric mean of pairwise ratios method (Chen et al. 2018). PERMANOVA was used separately for the two soil types (clay vs. silt) to test the effect of sampling time (second and third sampling), and the type of organic amendment (B, BH, BA, BHA) on fungal and bacterial community composition having the sample id (microcosm nr) as the strata. Bray-Curtis distance matrices were used in the *adonis* function from *vegan* 2.5–5 (Oksanen et al. 2020) with 999 permutations. Pairwise comparisons between organic amendments were calculated with the *pairwise.adonis2* function using the same explanatory and random factors as for *adonis* (Martinez Arbizu 2020). Homogeneity of variances was studied with *betadisper* function for soil type, organic amendment type and sampling time separately. We conducted 3-D NMDS with stable solution from random starts, and axis scaling and species scores with the metaMDS function from *vegan* using the Bray-Curtis dissimilarity index for visualization fungal and bacterial community composition. Differential abundant bacterial and fungal OTU for microcosms from the second and third separate samplings were obtained from the phyloseq object by differential abundance analysis (DESeq2), which identified significantly abundant groups for control versus four different organic amendments, and for silt and clay soil separately (>|2.0| log2 fold change with adjusted *P* < 0.001) (Love et al. 2014, McMurdie and Holmes 2014).

## Results

### Microbial abundances

**Figure 1.**
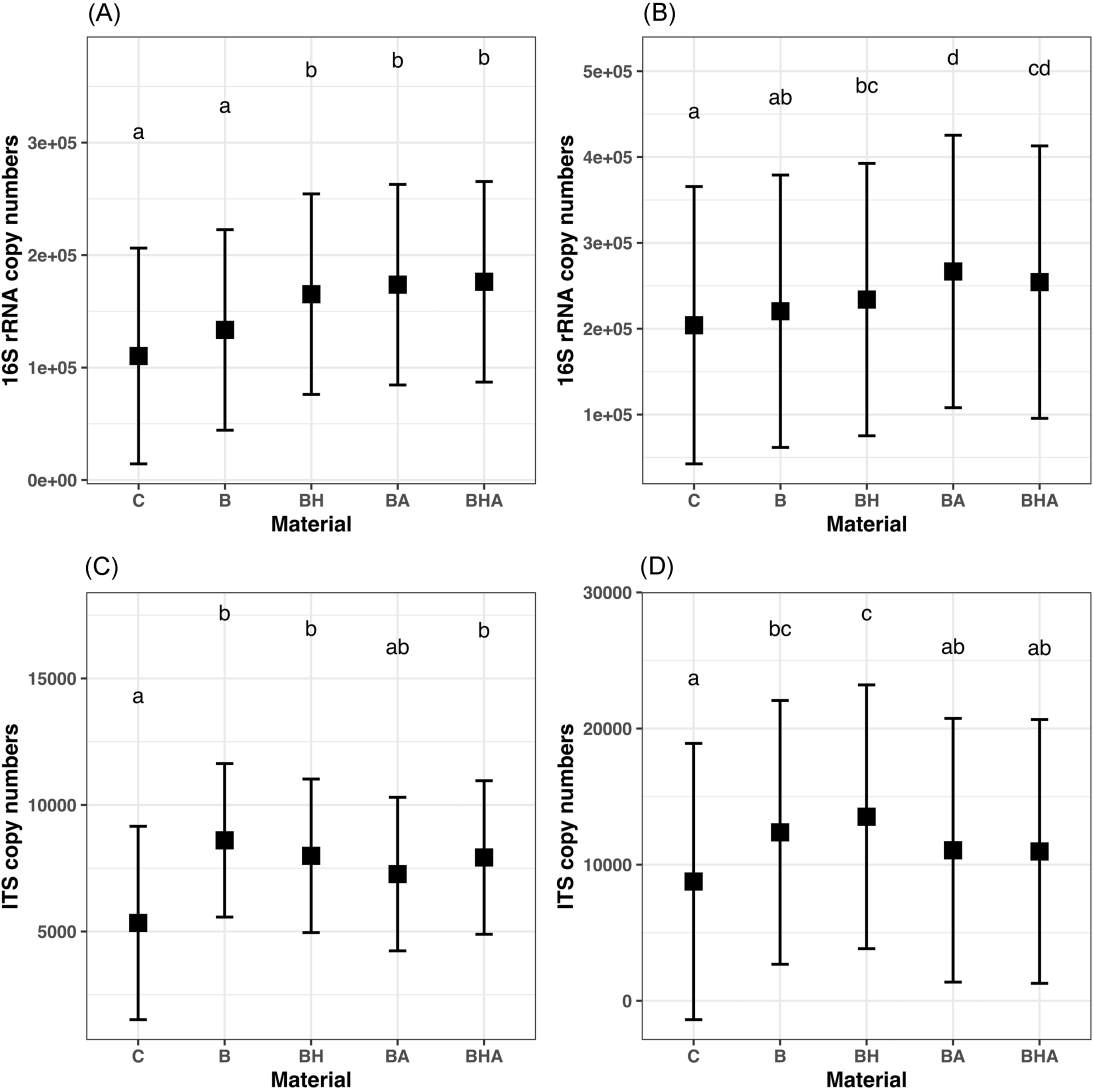
Estimated marginal means obtained with linear model from square root transformed bacterial 16S rRNA gene copy amounts per gram of dry soil matter (d.m.s.) in (**A**) silt and (**B**) in clay soil and fungal ITS region copy amounts per gram of d.m.s. in (**C**) silt and (**D**) in clay supplied with different bark-derived amendments. Error bars indicate the 95% confidence interval of the mean. Means sharing a letter are not significantly different. Data obtained from pine and spruce conifer bark are combined. See abbreviations in Table 1

Pine- and spruce-derived materials induced similar changes (data not shown) in qPCR and therefore the data were combined in the analyses as presented in Fig. 1. The average bacterial 16S rRNA and fungal ITS region copy numbers were lower in silt soil compared with clay soil (Fig. 1). Average bacterial 16S rRNA gene copy numbers increased in microcosms with all other bark-derived amendments, except in those with industrial bark (B) in silt and clay soils (Fig. 1a and b, Table S1). By contrast, average fungal ITS numbers increased in the silt soil microcosm with almost all the bark-derived amendments (B, BH, BHA), whereas average fungal ITS numbers in clay soil increased only in the microcosm with B and BH treatments (Fig. 1c and d, Table S1). Shoot biomasses of barley were significantly higher in microcosms with all amendments in clay soil and in microcosms with BA amendment in silt soil compared with controls (Supplementary Fig. S2).

### Microbial community composition

**Figure 2.**
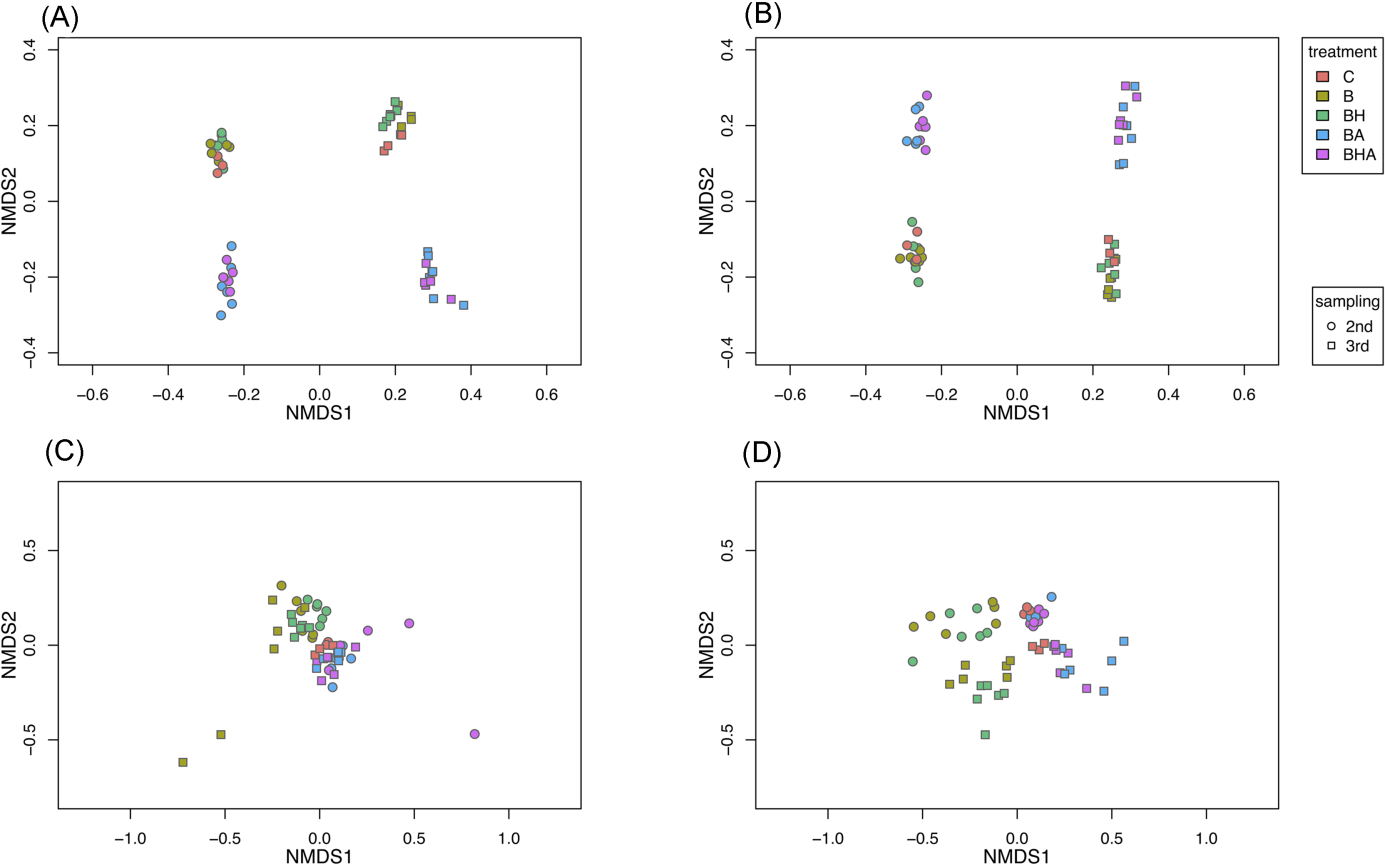
Non-metric multidimensional scaling (NMDS) analysis for bacterial 16S rRNA derived data from microcosms (**A**) in silt soil for axes 1 and 2, (**B**) in clay soil for axes 1 and 2, and for fungal ITS derived data from microcosms (**C**) in silt soil for axes 1 and 2, (**D**) in clay soil for axes 1 and 2. Data from the spring (second) and autumn (third) samplings are presented as circle and square symbols, respectively. See abbreviations in Table 1. The data for samples from pine and spruce derived amendments are combined

The NMDS for the bacterial 16S rRNA data showed clearly that the bacterial communities in the simulated spring and autumn (second and third samplings) separate from each other in both silt and clay soil (Fig. 2a and b). Axes 1 and 2 of the NMDS also showed that the bacterial community of B and BH amendments were more similar and separate from the communities of the microcosms with BA and BHA amendments. By contrast, while the NMDS of the fungal ITS2 data also separated the fungal communities of all amendments, the effect of sampling time was not so drastic compared with bacteria (Fig. 2c and d).

Sampling time explained 35% of the variation in bacterial community composition in both silt and clay soil (PERMANOVA, df = 1, F = 37.1/37.6, R2 = 0.35, *P* < 0.001) and the organic amendment type 19% and 18%, respectively (df = 4, F = 4.9/4.8, R2 = 0.19/0.18, *P* < 0.001). In both soil types sampling time explained 8% of the variation in fungal community composition (df = 4, F = 5.9/6.6, R2 = 0.08, *P* < 0.001). The type of amendment explained 25%, and 29% of the variation in fungal community composition in silt and clay soil, respectively (df = 4, F = 4.5/6.6, R2 = 0.25/0.29, *P* < 0.001). The interaction term was significant for both soil types explaining 8% of the variation in fungal community composition (df = 4, F = 1.5, R2 = 0.08, *P* < 0.017).

According to paired comparisons, bacterial communities did not differ in silt soil between B and BH, or BA and BHA (Table S2). Both soil amendment type and sampling time affected other bacterial communities for all amendments in clay soil. Whereas, both amendment type and sampling time affected other fungal communities for almost all amendments in silt soil; BA and BHA differed only by sampling time (Table S3). In turn, fungal communities of C were statistically undistinguishable from both B and BH, and B differed from both BH and BHA only by sampling time in clay soil.

### Differentially abundant bacterial OTUs

**Figure 3.**
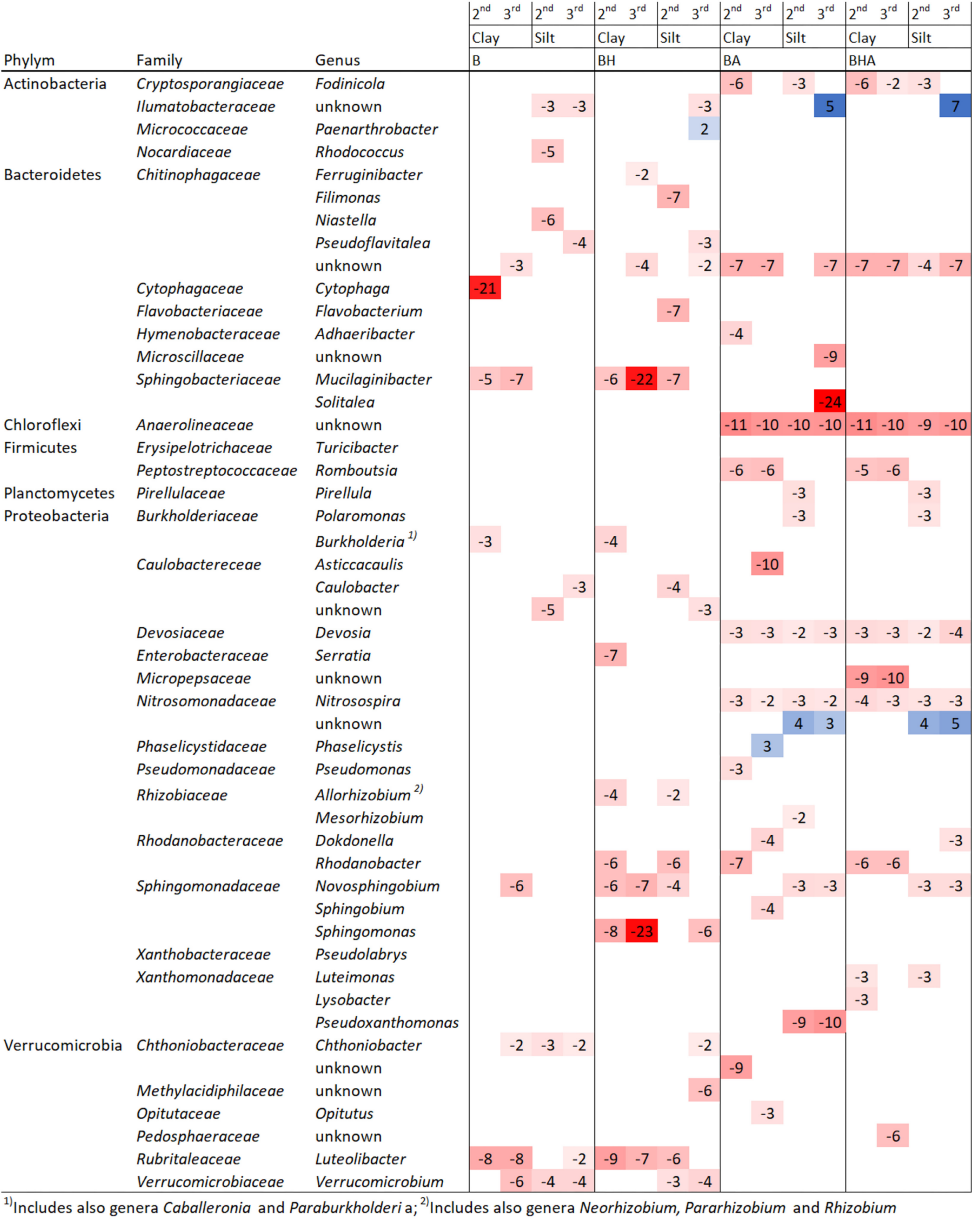
Heatmap showing the most significant bacterial 16S rRNA derived taxa (*P* adj < 0.001) for each microcosm amendment treatment according to differential abundance analysis. At maximum, 10 of the most differentially abundant taxa identified for each treatment are shown at phylum, family and genus level if possible (the full list can be found in Supplementary Table S2). Shown log2fold change (l2fc) values are from paired comparisons between control soils (clay or silt) with four different bark-derived amendments (B, BH, BA, BHA). Red colored values refer to representative taxa for the microcosm with organic amendments and blue colored values for the control soils. Only one value is represented if there were multiple different OTUs with significant values for the same taxa. Results are shown for the simulated spring (second) and autumn after harvest (third) samplings separately. See other abbreviations in Table 1. The data for samples from pine and spruce derived amendments are combined.

Taxa from 10 of the most differentially abundant bacterial OTUs for each microcosm from paired comparisons are presented in Fig. 3. The full list of all significant differentially abundant bacterial OTUs and their closest association to taxa in databases are shown in Table S4. When comparing control soils with microcosms having organic bark-derived amendments, there were less differentially abundant OTUs for B and BH treatments compared with those for BA and BHA treatments (Fig. 3, Table S4). There were differentially abundant OTUs for both soil types from all treatments although they were among 10 of the most significant ones only in some treatments (Fig. 3, Table S4). Those included OTUs representing, for example, genera of Verrucomicrobia (*Chthoniobacter)*, Proteobacteria (*Novosphingobium*) and Bacteroidetes (*Mucilaginibacter*), which were more differentially abundant in B and BH treatments.

Among the most significant differentially abundant OTUs in both clay and silt soil with B or BH amendments were two genera of Verrucomicrobia and Rhizobiaceae, but OTUs for B amendments represented genera of Bacteroidetes, *Cytophaga* in clay and *Niastella* in silt. These were recorded only from samples of the simulated spring (second sampling). Differentially abundant OTUs for BH amendments included the representative genera of Proteobacteria *Serratia* in clay and Bactoiredetes (*Filimonas*) and Verrucomicrobia (*Methylacidiphilaceae*) in silt.

In general, differentially abundant OTUs for BA and BHA treatments were more diverse compared with those for B or BH treatments. These included representatives of many phyla, for example, Cloacimonetes, Fibrobacteres, Firmicutes, Plantomycetes and Synergistetes, which were not recorded from B or BH treatments. OTUs in both soil types with both BA and BHA treatments included representative genera of, for example, Actinobacteria (*Fodinicola*), Chloroflexi (*Anaerolineaceae*), Planctomycetes (*Pirellula*), Proteobacteria (*Devosia, Nitrosospira, Luteimonas, Lysobacter, Mesorhizobium, Pseudoxanthomonas*) and Verrucomicrobia (*Opitutus*). Differentially abundant OTUs for both BA and BHA treatments included genera of Firmicutes (*Romboutsia*) and Bactoidetes (*Adhaeribacter*) in clay soil, and a genus of Proteobacteria (*Polaromonas)* in silt soil.

### Differentially abundant fungal OTUs

**Figure 4.**
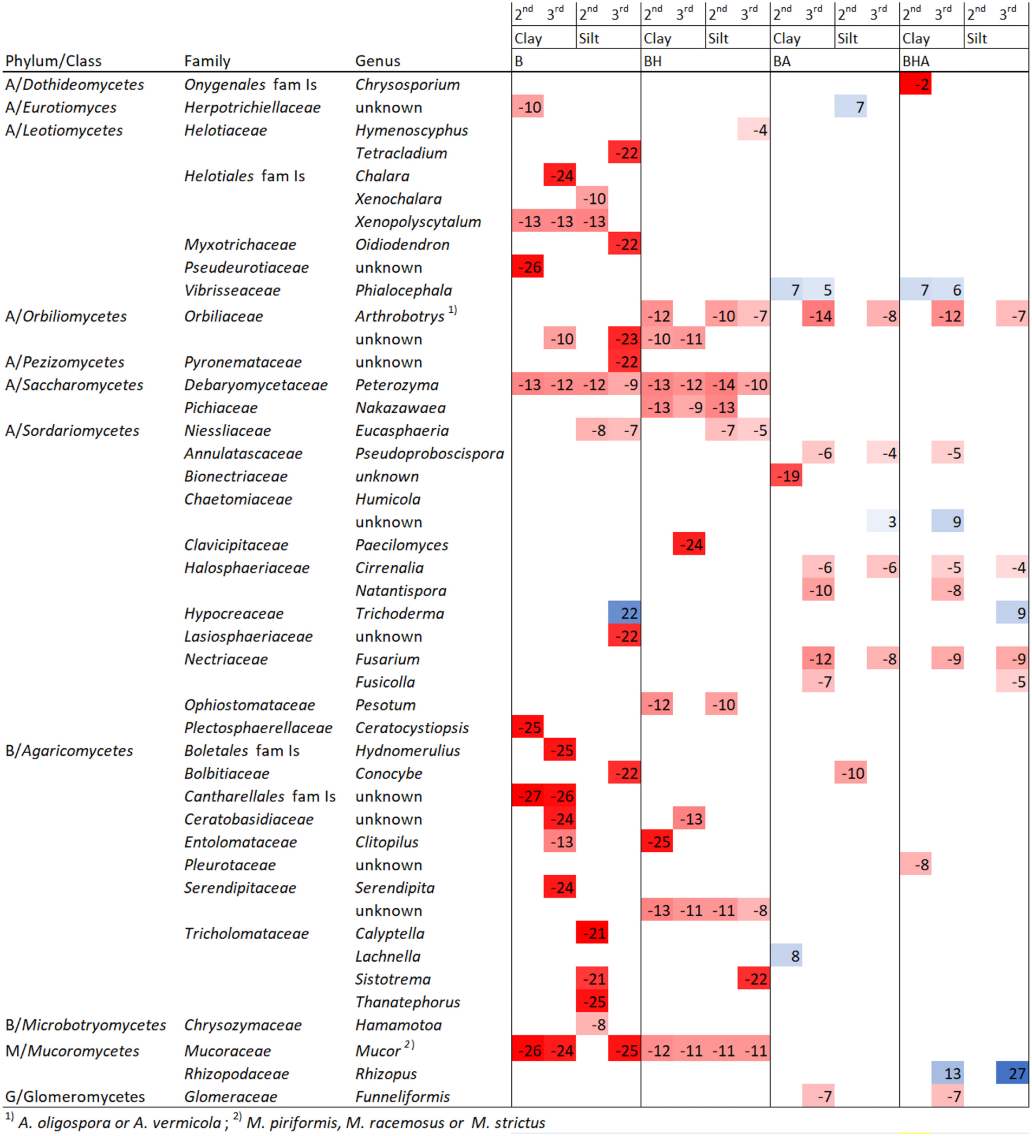
Heatmap showing the most significant fungal ITS derived taxa (*P* adj < 0.001) for each microcosm treatment according to differential abundance analysis. At maximum, 10 of the most differentially abundant taxa identified for each treatment are shown at phylum, class, family and genus level if possible (the full list can be found in Supplementary Table S3). Shown log2fold change (l2fc) values are from paired comparison between control soils (clay or silt) with four different bark-derived amendments (B, BH, BA, BHA). Red colored values refer to representative taxa for organic amendments and blue colored values for control soils. Results are shown for the simulated spring (second) and autumn after harvest (third) samplings separately. Only one value is represented if there were multiple different OTUs with significant values for the same taxa. Abbreviations: A, Ascomycota; B, Basidiomycota; G, Glomeromycota; M, Mucoromycota; R, Rozellomycota; fam Is; family Insertae sedis. See other abbreviations in Table 1. The data for samples from pine and spruce derived amendments are combined.

Taxa from 10 of the most significant differentially abundant fungal OTUs for each microcosm from paired comparisons are presented in Fig. 4. The full list of all significant differentially abundant OTUs and their closest associations to taxa in databases are provided in Supplementary Table S3. When comparing control soils and microcosms with bark-derived amendments, there were more differentially abundant fungal OTUs in microcosms with B and BH treatments compared with those with BA and BHA treatments (Fig. 4, Table S5). Differentially abundant fungal OTU in control soils for microcosms with B amendment were represented by the genus *Trichoderma*(*T. ivoriense*) in silt. Differentially abundant OTUs that were detected both in silt and clay soils with B or BH amendments comprised many representatives, including *Hymenoscyphus, Mucor, Oidiodendron, Peterozyma* and members of the family Serendipitaceae. Differentially abundant OTUs with B or BH amendments were represented by the genera *Clitopilus* and *Serendipita* in clay soil and *Eucasphaeria* and *Sistotrema* in silt.

Among the most significant differentially abundant OTUs detected in both soil types with B amendment were representatives of the genus *Xenopolyscytalium*. OTUs that were more abundant in clay soil with B amendment included, for example, the genera *Ceratocystiopsis, Chlara* and *Hydnomerulius*. Differentially abundant OTUs representing two of the first-mentioned taxa were observed only in samples from the simulated spring (second sampling) and that of the last-mentioned in samples from autumn after harvest. Among the most significant differentially abundant OTUs in silt soil with B amendment were representatives, for example, of the genus *Tetracladium* of the family Helotiaceae, and of the genera *Thanatephorus* and *Calyptella* of the family Tricholomataceae. Differentially abundant OTUs for BH amendment included representatives of, for example, the genus *Pesotum* in both soil types, and the genus *Paecilomyces* in clay.

Differentially abundant OTUs in control clay soils compared with microcosms with BA and BHA amendment included representatives of the genus *Phialocephala* (*P. humicola*) and, correspondingly, in control soils compared with microcosms with BHA amendment, representatives of the genera *Trichoderma* (*T. hamatum*) in silt and *Rhizopus* both in silt and clay. Among the most significant differentially abundant OTUs in both soil types with both BA or BHA amendments were representatives of the genera *Cirrenalia, Fusarium* (*F. solani*) and *Pseudoproboscispora*. The first-mentioned genera were detected in simulated spring samples (second sampling) and the latter after the harvest in autumn (third sampling), and differentially abundant OTUs in clay with both BA and BHA included the genera *Natantispora* and *Funneliformis*. The two latter-mentioned taxa were observed only in samples from autumn after harvest (third sampling). Differentially abundant OTUs for BA amendment were represented by the family Bionectriaceae, and for BHA amendment the genus *Chrysosporium* in clay.

## Discussion

### Factors affecting microbial abundance and community composition in microcosms

The purpose of this experiment was to study if synergies exist between three very timely global issues, recycling of organic material from industrial side-streams, to promote the circular economy and security of supply and to counteract the degradation of agricultural soils. We studied the potential of cascade process materials as soil amendments, meaning forest industry coniferous bark by-products untreated or treated in three incremental ways, in supporting microbial activity and diversity in agricultural soils. Our microcosm experiment simulated an over 1.5-year period of boreal arable soil and is one of the first attempts to investigate soil microbial communities after amendment with bark-derived organic materials. The addition of bark-derived organic amendments changed both the size and composition of the soil microbial communities and was supporting the crop yield. According to our hypothesis, community changes differed for bacteria and fungi and were linked to the soil type, especially for fungi. Industrial unextracted bark (B) and hot water extracted bark (BH) changed fungal community composition in silt soil and the abundance in clay soil, whereas all processed bark materials (BH, BA, BHA) had a greater influence on the bacterial numbers, and bark materials from anaerobic digestion (BA, BHA) on bacterial community composition. The obtained results show the same trend as previous findings, that anaerobic digestates used as biofertilizers increased bacterial gene copy numbers (Coelho et al. 2020). Increased bacterial abundance in microcosms with treated amendments may be partially explained by reduction of inhibiting polyphenolic compounds after cascade processing. The applied hot water treatment aimed at extraction of water-soluble polyphenols for further added-value use. At the same time, the extraction treatment may also have broken the structures of the bark, thus facilitating the microbial digestibility/degradability of the bark (Rasi et al. 2019, Jyske et al. 2020).

However, because the B/BH, and the two other BA/BHA amendments that contained processed slurry, were not added at the same C ha^−1^ rate and induced a highly different microcosm soil pH and soil C and N content, the results are not comparable between the two amendment types and must also be compared with the control (C) treatment. This comparison indicated for the bacterial community that the BA/BHA treatments in both soils and for the fungal community that the B/BH treatments induced a change in silt soil. The BA/BHA addition increased soil pH compared with the C treatment, whereas the B/BH treatment decreased it. It is known that bacterial community changes are correlated to pH, whereas fungal community changes are not to that extent, due to a broader pH tolerance (Rousk et al. 2010). This could imply that the B/BH-induced changes to the fungal community in silt soil were due to other reasons than pH, like, for instance, the bark material itself, and therefore this has the potential to trigger soil C acquisition through changed fungal presence. Phospholipid fatty acid profiling has shown that Gram-positive bacteria increased the C incorporation into temperate beech forest soil, contributing to the C stock of the entire soil profile (Preusser et al. 2021) and thus also bacteria are important, but our qPCR and amplicon sequencing approach does not quantitatively differentiate between Gram-positive and -negative bacteria.

As expected, soil type was one of the determinants affecting both bacterial and fungal abundance and especially fungal community composition (for previously reported soil microbiota see Pakarinen et al. 2021, Rasa et al. 2021). Both fungal and bacterial gene copy numbers were higher in clay compared with silt soil. Indeed, clay properties include unique physical and chemical characteristics, such as water-retention and cation exchange capacities, surface-to-volume ratio, ability to serve as a reservoir of adsorbed organic C, and by being so the clay minerals are the key in the interaction between microorganisms and the lithosphere (Cuadros 2017). The microbial interactions with the clay minerals are a fundamental component of the processes of soil genesis and functioning because clay can significantly alter microbial growth and biosynthetic activity by facilitating nutrients and providing protection against unfavorable physico-chemical conditions (reviewed by Fomina and Skorochod 2020). Sampling time, spring before sowing or autumn after harvest, affected bacterial community composition more than that of fungi. Therefore, type of arable soil and season must always be considered when estimating the effects of agricultural management on microbial communities (Bossio et al. 1998).

### Effect of amendments and soil type on bacterial taxa

The results indicated that there are bacteria that can take advantage of very different qualities of bark-derived amendment, from fresh industrial to highly processed bark material (also containing digested slurry) originating from anaerobic digestion. These included, for instance, cellulolytic *Mucilacibacter*, which are active in the decomposition of cellulose and hemicellulose (López-Mondéjar et al. 2016), *Chthoniobacter* (*C. flavus*), which grows on many of the saccharides found in plant biomass (Sangwan 2004), and *Novosphingobium*, which promotes plant growth and can degrade lignin-related and xenobiotic aromatic compounds (Tiirola et al. 2002, Hashimoto et al. 2010, Notomista et al. 2011, Choi et al. 2015). Also, the denitrifier genus *Rhodanobacter* was most abundant after all amendments and it contains members capable of complete denitrification of nitrate, nitrate and N_2_O to N_2_ (Prakash et al. 2012). The detected representatives include the same functionally important microorganisms that were also detected earlier in the digestates, such as plant-growth promoting, denitrifying and cellulolytic bacteria (Coelho et al. 2020).

However, the results suggest that there are bacteria that prefer unextracted bark (B) or hot water extracted bark (BH) amendments. These included *Niastella* and *Cytophaga*, which contain soil plant-associated bacteria or endophytic bacteria and are involved in the decomposition of plant-derived compounds (cellulose, chitin and pectin) (Reichenbach and Dworkin 1991). Similarly, to the sawdust amendments reported by Clocchiatti et al. (2021), BH treatment seemed to benefit Rhizobiaceae, common nitrogen-fixers associated with roots of legumes and other flowering plants. Some members of this group are also able to solubilize phosphorus (Sridevi and Mallaiah 2009). Other examples of bacteria that may increase after BH treatment were *Filimonas*, which have been detected from plant roots and may act as putative carbohydrate degraders, and *Serratia*, which are known to have antifungal properties, promote nitrogen-fixing symbionts as plant growth promoting bacteria and act as insect pathogens (Kalbe et al. 1996; Zhang et al. 1997, Grimont and Grimont 2006). Another taxon, *Methylacidiphilaceae*, includes verrucomicrobial methanotrophs that can oxidize methane (Op den Camp et al. 2009), which makes them important in the greenhouse gas (GHG) balance. These examples of bacterial taxa suggest that adding unprocessed bark, or even bark from which water-soluble carbohydrates are extracted for bioenergy use, to arable soil, has the potential to increase beneficial soil bacteria, promote nitrogen and carbon cycling and benefit plants directly.

The results show clearly that the soil bacterial community comprises a diverse group of bacteria that can take advantage of highly processed bark material with high pH and N content. For example, amendments originating from the anaerobic digestion process (BA, BHA) seem to increase some representatives of the Planctomycetes that are distributed in a variety of habitats, and some are known to grow anaerobically and autotrophically via oxidation of ammonium (Fuerst 2004). In addition, degraders of many toxins *Devosia*, nitrite oxidizing *Nitrospira* and strictly anaerobic *Anaerolineaceae* were detected. The genus *Devosia* was reported to enrich soils applied with manure containing the antibiotic compound sulfadiazine (Ding et al. 2014). Interestingly, a syntrophic relationship between hydrogenotrophic methanogens and species of *Anaerolineaceae* was reported (Yamada and Sekiguchi 2018), which are probably essential microbial partners in the anaerobic digestion process. Thus, most likely at least some of the observed anaerobes originate from the anaerobic digestion process. However, we detected OTUs representing Cloacimodetes, Firmicutes and Synergistetes only from microcosms with BA or BHA amendments, both of which are commonly found in biogas reactors (Solli et al. 2014). The community from the highly processed bark material, from the anaerobic digestion process, dominated the pre-amendment soil community and could be an example of community coalescence introduced by Rillig et al. (2015).

There were several bacterial taxa that were apparently season or season and soil specific. For example, *Lysobacter* species, detected in B and BHA treatments only in simulated spring before sowing, can produce a range of extracellular enzymes and metabolites that are active against other soil organisms and that are more abundant in soils that suppress the fungal root pathogen *Rhizoctonia solani* (Gómez Expósito et al. 2015). However, *Lysobacter* species were detected previously and were indicative for autumn bulk soil in the same field (Pakarinen et al. 2021), from which the soil for this experiment was collected. In turn, anaerobic *Romboutsia* seemed to be clay specific and showed differential abundance in autumn after harvest. Some species have been isolated from the anaerobic digestion process as well as from soil (Dabrowski et al. 2012, Gao et al. 2014, Gerritsen et al. 2019). Members of the *Romboutsia* genus seem to have a versatile array of metabolic capabilities with respect to carbohydrate utilization, fermentation of single amino acids, anaerobic respiration and end products (Gerritsen et al. 2019). Thus, it may be very important in which period of the growing season organic amendments are applied to the fields in terms of which microbes’ benefit.

It has been demonstrated that Gram-positive bacteria have raised C incorporation into subsoil over time (Preusser et al. 2021). Thus, Gram-positive bacteria are suggested to be better adapted to resource-limited conditions and feed on previously processed C sources (Kramer et al. 2008, Wang et al. 2014), whereas Gram-negative bacteria prefer to use labile C sources (Creamer et al. 2016). We detected only a few OTU representatives from the major Gram-positive bacterial phyla Actinobacteria and Firmicutes, which were more abundant in microcosms with BA and BHA amendments. However, the majority of differentially abundant OTUs detected from microcosms with amendments were Gram-negative bacteria, such as Proteobacteria, Bacteroidetes, Verrucomicrobia and Planctomycetes. BA and BHA digestates may provide more labile C sources for Gram-negative bacteria, which can quickly represent a relatively high proportion of the microbial biomass (Elfstrand et al. 2008). Subsequently, slow-growing Gram-positive bacteria can utilize more recalcitrant substrates and form a stable C stock. However, differential abundance results cannot be treated as truly quantitative data and thus the true ratio of Gram-positive to Gram-negative bacteria cannot be estimated with this dataset.

### Effect of amendments and soil type on fungal taxa

A closer look at the differentially abundant fungal OTUs for B or BH amendments revealed that many fungal representatives may originate from bark-associated insects. For example, one of these representatives from the genus, *Peterozyma toletana*, is a common yeast found in the great spruce bark beetle (*Dendroctonus micans*) (Menkis et al. 2017). Also, other observed yeast-like representative genera, for example, *Ceratocystiopsi*s and *Pesotum* (anamorph of *Ophiostoma*), have also been reported to be associated with the spruce bark beetle *Ips typographus* (Viiri and Lieutier 2004). The genus *Mucor* was detected as a representative taxon in decomposed wood blocks and suggested to contribute to wood decomposition via the breakdown of complex sugars (Fukasawa et al. 2011, Gómez-Brandón et al. 2020). Among other saprotrophic representative genera for bark material is the cellulose-degrading genus *Chlara* (Boberg et al. 2011), the genus *Xenopolyscytalum*, an indicator of near mature and mature forests (Zhao et al. 2020), *Hydnomerulius*, a brown rot cellulose and hemi-cellulose degrader (Kohler et al. 2015), and *Clitopilus* (Raj and Manimohan 2018), which promotes high biomass in spruce monocultures planted on former arable land (Mihál 1998). Saprotrophic fungi that originated from the bark-derived organic amendment are certainly important in improving the quality and health of arable soil because they can help decrease losses of mineral nutrients (de Vries et al. 2011) and increase C sequestration (Six et al. 2006) and water retention (Beare et al. 1997, Helfrich et al. 2015, Liao et al. 2018). Moreover, saprotrophic fungi contribute to the suppression of root-infecting fungal pathogens (van Beneden et al. 2010, Xiong et al. 2017, Siegel-Hertz et al. 2018). Thus, the ability of saprotrophic fungi to attack recalcitrant wood-derived polymers like hemicellulose, cellulose and lignin gives them an advantage to survive in the environment. Indeed, incorporation of wood-derived material in arable soil was associated with increased saprotrophic fungal biomass (van der Wal et al. 2007, Moll et al. 2015, Reardon and Wuest 2016).

Interestingly, a *Hymenoscyphus*, containing dark-septate endophyte (DSE) fungi, was the representative for silt soil microcosms with BH amendment in autumn after harvest. DSE fungi produce dark melanized hyphae and are found inside plant roots or in rhizosphere soil (Berthelot et al. 2019), and frequently co-occur with mycorrhizal fungi (Mandyam and Jumpponen 2005). DSE fungi can be found from a diverse range of ecosystems and host plants and are of a wide taxonomic and functional variety, providing nutrients for plants from SOM and protecting plants from pathogens and harsh soil conditions (Berthelot et al. 2019). Because of their ability to produce highly melanized and recalcitrant hyphae, DSE fungi have an important role in C sequestration into more stable SOM (Siletti et al. 2017). DSE fungi are suggested to link plant roots to soil crusts, fixing carbon and nitrogen via the hyphal network and representing the basis of the Fungal Loop Hypothesis (Porras-Alfalo et al. 2011). Like mycorrhizal fungi, DSE fungi can protect hosts through the production of antibacterial or antifungal metabolites, physical exclusion of other microorganisms, or melanized hyphae (Mandyam and Jumpponen 2005). In addition, another two representative OTUs for the microcosms with the B and BH amendment in autumn after harvest were serendipitoid fungi (genus *Serendipita*, family Serendipitacea), which are known for their mycorrhizal and endophytic associations with a variety of plant species (Craven and Ray 2019). Serendipitoids enhance the growth and stress resistance of barley by upregulating several proteins involved in photosynthesis and carbohydrate metabolism (Sepehri et al. 2021). It was hypothesized that serendipitoids would be able to decompose SOM, contributing to efficient organic matter (OM) turnover and preventing unnecessary losses of C and nutrients (Craven and Ray 2019). Serendipitoids have recently been recorded after fiber sludge amendment in another Finnish agricultural soil, confirming their relevance in forest-derived organic material (Heikkinen et al. 2021).

Interestingly, a common arbuscular mycorrhizal fungi (AMF) genus, *Funneliformis*, was representative for microcosms with BA and BHA amendments after harvest. *Funneliformis* is common in neutral and slightly alkaline soils (Mukerji et al. 2002), and the BA/BHA material was originally very alkaline. On the other hand, BA/BHA materials have introduced excess N (originating from cattle slurry) into soil, and N addition has been noted to reduce the diversity and richness of AMF and suppress spore numbers and hyphal length density (Zhang et al. 2016). Furthermore, increased abundance of *Funneliformis* has been detected 3 years after pulp-mill sludge amendment to a field (Rasa et al. 2021) and Clocchiatti et al. (2021) reported an increase in AMF following sawdust amendment of agricultural soil. Overall, the results suggest that unextracted or mildly treated organic amendments may have the potential to introduce fungi that could promote resilience and sustainability of arable soil.

However, unprocessed bark could potentially introduce plant pathogens (Kazartsev et al. 2018). Increased abundance of *Thanatephorus cucumeris*, which is a teleomorph of the important plant pathogen *Rhizoctonia solani*, was detected in a microcosm with unextracted bark (B). In turn, another *Fusarium* species (*F. solani*) was representative for microcosms with BA and BHA amendments in autumn after harvest. Yet another *Fusarium* species (*F. culmorum*) was representative for control clay soil compared with the microcosm associated with B and BH amendments. *Fusarium culmorum* produces head blight, especially on small-grain cereals such as barley (Scherm et al. 2013). Because species of *Fusarium* are also saprotrophs like *Rhizoctonia*, they might have benefited from fresh plant material after harvest from root and shoot residues in addition to the bark-derived amendment. Thus, the results suggest that the response of fungi depends on the quality of organic material as well as on the type of pathogen.

Along with increased abundance of positive and beneficial fungi from bark-derived amendments, the amendments might also decrease the abundance of some soil fungi. For example, *Phialocephala, Rhizopus* and *Trichoderma* were representative for control soils without bark-derived amendments. Species of *Phialocephala* include common soil fungi and well-known dark-septate endophytes*. Trichoderma* include plant-growth-promoters that suppress plant diseases and have been widely used as biocontrol agents (Zin and Badaluddin 2020). Most *Rhizopus* species are saprotrophs, feeding on a variety of dead organic matter, but some species are also parasitic or pathogenic (Petruzzello 2016). Our results may be explained by increased competition among fungi, that is, bark-derived organic compounds could favor some fungi over others. Another explanation could be that conditions created by bark-derived amendments may have induced these fungi to act as endophytes, resulting in their decreased abundance in soil.

## Conclusions

Our microcosm experiment, which simulated the time span within one growing season, shows that bark-derived organic amendments from sawmills have the potential to both increase the biomass and diversify the communities of agricultural soil microbes. Most of the stimulated microbes represented groups that are known for their beneficial impacts on plants and soil, such as symbiotic AMF and N-fixing bacteria. The treatment effects were largely dependent on the intensity of the bark processing and the type of soil. Soil bacterial communities were mostly induced by highly processed bark treated anaerobically with cattle slurry, whereas the response of soil fungal community to fresh and mildly treated hot water extracted bark was dependent on soil type. These differences may be due to soil pH, the higher-level processing, eliminating the indigenous microbiota of bark, changing the bark to be less recalcitrant for decomposers and having compounds originating from cattle slurry that favored bacteria. If fungi are to be favored, then addition of bark or hot water extracted bark are the potential organic amendments to choose. However, our study was a short-term laboratory experiment, and field studies are needed to verify our observations and particularly to estimate the long-term effects of the side-streams from sawmills and biogas production. Our study shows the way towards seeking new solutions for increasing the sustainability of soils by utilizing by-products more efficiently.

## Supplementary Material

fiad012_Supplemental_File
