# Supplementary material for: Impacts of coniferous bark-derived organic soil amendments on microbial communities in arable soil – a microcosm study
Source: FEMS Microbiol Ecol. 2023 Feb 1;99(3):fiad012. doi: 10.1093/femsec/fiad012 (PMC10013654; doi:10.1093/femsec/fiad012)
Supplement: fiad012_Supplemental_File [file fiad012_supplemental_file.docx]

**Supplementary information**

**Table S1.** The results from linear mixed-effects model fit by maximum likelihood that were used to test the effect of bark-derived organic soil amendments on bacterial 16S rRNA and fungal ITS2 gene copy amounts in microcosms of silt and clay soil at a significance level of *p* ≤0.05. Dunnett’s post-hoc comparisons between the control (C) and the four amendments. Abbreviations: B, unextracted conifer bark; BH, hot water extracted bark: BA, digestate containing untreated bark from an anaerobic digestion process; BHA, digestate containing hot water extracted bark from an anaerobic digestion process.

Data Soil type Amendment Estimate SE z-value p-value

^_______________________________________________________________________________________________________________________^

16SrRNA Silt  B 23191 12253 1.893 0.234

BH 54978 12253 4.487 2.89e-05

BA 63410 12253 5.175 9.12e-07

BHA 65986 12253 5.385 2.90e-07

Clay B 16307 10472 1.557 0.478

BH 29876 10472 2.853 0.017

BA 62640 10472 5.982 8.8e-09

BHA 50232 10472 4.797 6.5e-06

ITS2 Silt  B 3266 802.1 4.072 1.86e-04

BH 2653 802.1 3.308 0.004

BA 1931 802.1 2.408 0.0642

BHA 2588 802.1 3.841 0.005

Clay B 3609 1043 3.46 0.002

BH 4756 1043 4.559 2.1e-05

BA 2293 1043 2.198 0.112

BHA 2207 1043 2.116 0.138

^__________________________________________________________________________________________________________________________^

**Table S2.** Results of PERMANOVA pairwise analyses with 999 permutations to test the effect of sampling time (2^nd^ and 3^rd^ sampling), and amendments and their interaction on bacterial 16SrRNA OTU composition in the soil microcosms separately for a-j) silt and i-t) clay. Differences are considered significant at *p* ≤ 0.05 after the P-values were Bonferroni corrected to consider multiple comparisons (B not significant after correction). The variances of different groups were equal.

a) C vs B (silt) Df SumOfSqs R2 F Pr(>F)

^________________________________________________________________________________________________________________^

Organic amendment 1 0.08341 0.04623 1.1861 0.002

Sampling 1 0.68300 0.37852 9.7119 0.002

Time:Amendment 1 0.05341 0.02960 0.7594 0.589

Residual 14 0.98457 0.54565

^________________________________________________________________________________________________________________^

b) C vs BH (silt) Df SumOfSqs R2 F Pr(>F)

^________________________________________________________________________________________________________________^

Organic amendment 1 0.10056 0.06214 1.6636 0.003

Sampling 1 0.68147 0.42112 11.2740 0.003

Time:Amendment 1 0.05040 0.03115 0.8338 0.502

Residual 13 0.78579 0.48559

^________________________________________________________________________________________________________________^

c) C vs BA (silt) Df SumOfSqs R2 F Pr(>F)

^________________________________________________________________________________________________________________^

Organic amendment 1 0.31772 0.13743 4.5079 0.001

Sampling 1 0.90425 0.39113 12.8299 0.001

Time:Amendment 1 0.10323 0.04465 1.4647 0.165

Residual 14 0.98672 0.42680

^________________________________________________________________________________________________________________^

d) C vs BHA (silt) Df SumOfSqs R2 F Pr(>F)

^________________________________________________________________________________________________________________^

Organic amendment 1 0.32000 0.15271 5.1595 0.001

Sampling 1 0.83749 0.39967 13.5031 0.001

Time:Amendment 1 0.06965 0.03324 1.1230 0.269

Residual 14 0.86831 0.41438

^________________________________________________________________________________________________________________^

e) B vs BH (silt) Df SumOfSqs R2 F Pr(>F)

^________________________________________________________________________________________________________________^

Organic amendment 1 0.06038 0.02692 0.9833 0.391

Sampling 1 0.96369 0.42960 15.6941 0.001

Time:Amendment 1 0.05247 0.02339 0.8545 0.467

Residual 19 1.16668 0.52009

^________________________________________________________________________________________________________________^

f) B vs BA (silt) Df SumOfSqs R2 F Pr(>F)

^________________________________________________________________________________________________________________^

Organic amendment 1 0.49778 0.15767 7.2796 0.001

Sampling 1 1.12794 0.35726 16.4951 0.001

Time:Amendment 1 0.16383 0.05189 2.3958 0.038 (B)

Residual 20 1.36761 0.43318

^________________________________________________________________________________________________________________^

g) B vs BHA (silt) Df SumOfSqs R2 F Pr(>F)

^________________________________________________________________________________________________________________^

Organic amendment 1 0.50377 0.17109 8.0655 0.001

Sampling 1 1.05678 0.35891 16.9193 0.001

Time:Amendment 1 0.13466 0.04573 2.1559 0.056

Residual 20 1.24920 0.42426

^________________________________________________________________________________________________________________^

h) BH vs BA (silt) Df SumOfSqs R2 F Pr(>F)

^________________________________________________________________________________________________________________^

Organic amendment 1 0.53906 0.17998 8.7627 0.001

Sampling 1 1.17942 0.39378 19.1721 0.001

Time:Amendment 1 0.10780 0.03599 1.7524 0.132

Residual 19 1.16883 0.39025

^________________________________________________________________________________________________________________^

i) BH vs BHA (silt) Df SumOfSqs R2 F Pr(>F)

^________________________________________________________________________________________________________________^

Organic amendment 1 0.55667 0.19924 10.0690 0.001

Sampling 1 1.09575 0.39218 19.8199 0.001

Time:Amendment 1 0.09114 0.03262 1.6485 0.137

Residual 19 1.05042 0.37596

^________________________________________________________________________________________________________________^

j) BA vs BHA (silt) Df SumOfSqs R2 F Pr(>F)

^________________________________________________________________________________________________________________^

Organic amendment 1 0.04377 0.01587 0.6996 0.567

Sampling 1 1.41924 0.51466 22.6834 0.001

Time:Amendment 1 0.04326 0.01569 0.6914 0.572

Residual 20 1.25135 0.45378

^________________________________________________________________________________________________________________^

k) C vs B (clay) Df SumOfSqs R2 F Pr(>F)

^________________________________________________________________________________________________________________^

Organic amendment 1 0.09458 0.05969 1.6998 0.002

Sampling 1 0.62903 0.39697 11.3043 0.002

Time:Amendment 1 0.08193 0.05171 1.4724 0.134

Residual 14 0.77903 0.49163

^________________________________________________________________________________________________________________^

l) C vs BH (clay) Df SumOfSqs R2 F Pr(>F)

^________________________________________________________________________________________________________________^

Organic amendment 1 0.10149 0.06259 1.8175 0.001

Sampling 1 0.64304 0.39654 11.5153 0.001

Time:Amendment 1 0.09531 0.05877 1.7067 0.105

Residual 14 0.78180 0.48210

^________________________________________________________________________________________________________________^

m) C vs BA (clay) Df SumOfSqsR2 F Pr(>F)

^________________________________________________________________________________________________________________^

Organic amendment 1 0.31432 0.16008 5.4325 0.001

Sampling 1 0.69633 0.35463 12.0348 0.001

Time:Amendment 1 0.14283 0.07274 2.4686 0.028 (B)

Residual 14 0.81003 0.41254

^________________________________________________________________________________________________________________^

n) C vs BHA (clay) Df SumOfSqs R2 F Pr(>F)

^________________________________________________________________________________________________________________^

Organic amendment 1 0.31837 0.16785 6.5149 0.001

Sampling 1 0.72876 0.38420 14.9127 0.001

Time:Amendment 1 0.16554 0.08727 3.3874 0.008 (B)

Residual 14 0.68416 0.36069

^________________________________________________________________________________________________________________^

o) B vs BH (clay) Df SumOfSqs R2 F Pr(>F)

^________________________________________________________________________________________________________________^

Organic amendment 1 0.04588 0.02037 0.7589 0.001

Sampling 1 0.97027 0.43067 16.0481 0.001

Time:Amendment 1 0.02757 0.01224 0.4561 0.910

Residual 20 1.20920 0.53672

^________________________________________________________________________________________________________________^

p) B vs BA (clay) Df SumOfSqs R2 F Pr(>F)

^________________________________________________________________________________________________________________^

Organic amendment 1 0.41473 0.15077 6.7031 0.001

Sampling 1 1.02583 0.37292 16.5800 0.001

Time:Amendment 1 0.07281 0.02647 1.1768 0.292

Residual 20 1.23743 0.44984

^________________________________________________________________________________________________________________^

q) B vs BHA (clay) Df SumOfSqs R2 F Pr(>F)

^________________________________________________________________________________________________________________^

Organic amendment 1 0.47758 0.17411 8.5930 0.001

Sampling 1 1.06393 0.38788 19.1430 0.001

Time:Amendment 1 0.08985 0.03276 1.6167 0.153

Residual 20 1.11156 0.40525

^________________________________________________________________________________________________________________^

r) BH vs BA (clay) Df SumOfSqs R2 F Pr(>F)

^________________________________________________________________________________________________________________^

Organic amendment 1 0.40163 0.14510 6.4769 0.001

Sampling 1 1.04612 0.37795 16.8701 0.001

Time:Amendment 1 0.07992 0.02887 1.2888 0.239

Residual 20 1.24020 0.44807

^________________________________________________________________________________________________________________^

s) BH vs BHA (clay) Df SumOfSqs R2 F Pr(>F)

^________________________________________________________________________________________________________________^

Organic amendment 1 0.44288 0.16173 7.9489 0.001

Sampling 1 1.09410 0.39955 19.6371 0.001

Time:Amendment 1 0.08707 0.03179 1.5627 0.178

Residual 20 1.11432 0.40693

^________________________________________________________________________________________________________________^

t) BA vs BHA (clay) Df SumOfSqs R2 F Pr(>F)

^________________________________________________________________________________________________________________^

Organic amendment 1 0.07953 0.03176 1.3921 0.001

Sampling 1 1.23029 0.49132 21.5357 0.001

Time:Amendment 1 0.05168 0.02064 0.9047 0.353

Residual 20 1.14256 0.45628

^________________________________________________________________________________________________________________^

**Table S3.** Results of PERMANOVA pairwise analyses with 999 permutations to test the effect of sampling time (2nd and 3rd sampling), and amendments and their interaction on fungal ITS OTU composition in the soil microcosms separately for a-d) silt and e-g) clay. Differences are considered significant at p ≤ 0.05 after the P-values were Bonferroni corrected to consider multiple comparisons (B not significant after correction). The variances of different groups were equal.

a) C vs B (silt) Df SumOfSqsR2 F Pr(>F)

^________________________________________________________________________________________________________________^

Organic amendment 1 0.47643 0.20946 4.5363 0.002

Sampling 1 0.24146 0.10616 2.2990 0.001

Time:Amendment 1 0.08629 0.03794 0.8216 0.378

Residual 14 1.47035 0.64644

^________________________________________________________________________________________________________________^

b) C vs BH (silt) Df SumOfSqs R2 F Pr(>F)

^________________________________________________________________________________________________________________^

Organic amendment 1 0.55771 0.25452 5.9964 0.002

Sampling 1 0.22706 0.10362 2.4413 0.001

Time:Amendment 1 0.10437 0.04763 1.1221 0.186

Residual 14 1.30212 0.59423

^________________________________________________________________________________________________________________^

c) C vs BA (silt) Df SumOfSqs R2 F Pr(>F)

^________________________________________________________________________________________________________________^

Organic amendment 1 0.29774 0.14384 3.2511 0.003

Sampling 1 0.37048 0.17898 4.0454 0.001

Time:Amendment 1 0.11959 0.05777 1.3058 0.105

Residual 14 1.28212 0.61940

^________________________________________________________________________________________________________________^

d) C vs BHA (silt) Df SumOfSqs R2 F Pr(>F)

^________________________________________________________________________________________________________________^

Organic amendment 1 0.21741 0.08221 1.6451 0.001

Sampling 1 0.44352 0.16771 3.3561 0.002

Time:Amendment 1 0.13346 0.05047 1.0099 0.187

Residual 14 1.85014 0.69961

^________________________________________________________________________________________________________________^

e) B vs BH (silt) Df SumOfSqs R2 F Pr(>F)

^________________________________________________________________________________________________________________^

Organic amendment 1 0.23694 0.08410 2.1797 0.001

Sampling 1 0.30826 0.10941 2.8358 0.001

Time:Amendment 1 0.09819 0.03485 0.9033 0.481

Residual 20 2.17406 0.77164

^________________________________________________________________________________________________________________^

f) B vs BA (silt) Df SumOfSqs R2 F Pr(>F)

^________________________________________________________________________________________________________________^

Organic amendment 1 0.6985 0.20437 6.4851 0.001

Sampling 1 0.4092 0.11974 3.7997 0.001

Time:Amendment 1 0.1559 0.04560 1.4471 0.127

Residual 20 2.1541 0.63028

^________________________________________________________________________________________________________________^

g) B v BHA (silt) Df SumOfSqs R2 F Pr(>F)

^________________________________________________________________________________________________________________^

Organic amendment 1 0.6919 0.17017 5.0835 0.001

Sampling 1 0.3825 0.09408 2.8106 0.002

Time:Amendment 1 0.2695 0.06628 1.9799 0.014 (B)

Residual 20 2.7221 0.66948

^________________________________________________________________________________________________________________^

h) BH vs BA (silt) Df SumOfSqs R2 F Pr(>F)

^________________________________________________________________________________________________________________^

Organic amendment 1 0.8002 0.23853 8.0592 0.001

Sampling 1 0.3563 0.10621 3.5885 0.002

Time:Amendment 1 0.2125 0.06333 2.1398 0.028 (B)

Residual 20 1.9858 0.59194

^________________________________________________________________________________________________________________^

i) BH vs BHA (silt) Df SumOfSqs R2 F Pr(>F)

^________________________________________________________________________________________________________________^

Organic amendment 1 0.8412 0.20767 6.5877 0.001

Sampling 1 0.3964 0.09785 3.1040 0.002

Time:Amendment 1 0.2593 0.06402 2.0309 0.019 (B)

Residual 20 2.5539 0.63047

^________________________________________________________________________________________________________________^

j) BA vs BHA (silt) Df SumOfSqs R2 F Pr(>F)

^________________________________________________________________________________________________________________^

Organic amendment 1 0.2222 0.06223 1.7538 0.054 (B)

Sampling 1 0.6400 0.17925 5.0515 0.001

Time:Amendment 1 0.1743 0.04883 1.3761 0.178

Residual 20 2.5339 0.70969

^________________________________________________________________________________________________________________^

k) C vs B (clay) Df SumOfSqs R2 F Pr(>F)

^________________________________________________________________________________________________________________^

Organic amendment 1 0.60890 0.19498 4.1889 0.047 (B)

Sampling 1 0.31613 0.10123 2.1748 0.026 (B)

Time:Amendment 1 0.16286 0.05215 1.1204 0.207

Residual 14 2.03505 0.65165

^________________________________________________________________________________________________________________^

l) C vs BH (clay) Df SumOfSqs R2 F Pr(>F)

^________________________________________________________________________________________________________________^

Organic amendment 1 0.9027 0.27791 6.6978 0.009 (B)

Sampling 1 0.2995 0.09220 2.2222 0.004

Time:Amendment 1 0.1592 0.04900 1.1811 0.173

Residual 14 1.8868 0.58089

^________________________________________________________________________________________________________________^

m) C vs BA (clay) Df SumOfSqs R2 F Pr(>F)

^________________________________________________________________________________________________________________^

Organic amendment 1 0.40008 0.14462 3.6871 0.001

Sampling 1 0.62661 0.22651 5.7748 0.001

Time:Amendment 1 0.22054 0.07972 2.0324 0.008(B)

Residual 14 1.51911 0.54914

^________________________________________________________________________________________________________________^

n) C vs BHA (clay) Df SumOfSqs R2 F Pr(>F)

^________________________________________________________________________________________________________________^

Organic amendment 1 0.37811 0.17826 4.1639 0.001

Sampling 1 0.33614 0.15848 3.7017 0.001

Time:Amendment 1 0.13552 0.06389 1.4923 0.117

Residual 14 1.27131 0.59937

^________________________________________________________________________________________________________________^

o) B vs BH (clay) Df SumOfSqs R2 F Pr(>F)

^________________________________________________________________________________________________________________^

Organic amendment 1 0.3671 0.09003 2.3018 0.047(B)

Sampling 1 0.4444 0.10899 2.7866 0.004

Time:Amendment 1 0.0762 0.01870 0.4780 0.982

Residual 20 3.1896 0.78228

^________________________________________________________________________________________________________________^

p) B vs BA (clay) Df SumOfSqs R2 F Pr(>F)

^________________________________________________________________________________________________________________^

Organic amendment 1 0.9124 0.19649 6.4663 0.001

Sampling 1 0.5472 0.11785 3.8783 0.001

Time:Amendment 1 0.3619 0.07794 2.5650 0.005

Residual 20 2.8219 0.60773

^________________________________________________________________________________________________________________^

q) B vs BHA (clay) Df SumOfSqs R2 F Pr(>F)

^________________________________________________________________________________________________________________^

Organic amendment 1 0.9372 0.23170 7.2818 0.008(B)

Sampling 1 0.3572 0.08830 2.7750 0.001

Time:Amendment 1 0.1765 0.04363 1.3711 0.159

Residual 20 2.5741 0.63638

^________________________________________________________________________________________________________________^

r) BH vs BA (clay) Df SumOfSqs R2 F Pr(>F)

^________________________________________________________________________________________________________________^

Organic amendment 1 1.2408 0.25833 9.2818 0.001

Sampling 1 0.5450 0.11346 4.0767 0.001

Time:Amendment 1 0.3438 0.07158 2.5718 0.002

Residual 20 2.6737 0.55663

^________________________________________________________________________________________________________________^

s) BH vs BHA (clay) Df SumOfSqs R2 F Pr(>F)

^________________________________________________________________________________________________________________^

Organic amendment 1 1.3179 0.30958 10.8657 0.002

Sampling 1 0.3142 0.07382 2.5908 0.001

Time:Amendment 1 0.1991 0.04676 1.6412 0.028(B)

Residual 20 2.4259 0.56984

^________________________________________________________________________________________________________________^

t) BA vs BHA (clay) Df SumOfSqs R2 F Pr(>F)

^________________________________________________________________________________________________________________^

Organic amendment 1 0.14901 0.04793 1.4480 0.001

Sampling 1 0.79955 0.25718 7.7695 0.001

Time:Amendment 1 0.10224 0.03288 0.9935 0.436

Residual 20 2.05816 0.66201

^________________________________________________________________________________________________________________^

**Table S4.** Results from the differential abundance analysis showing all significant (*p* adj <0.001) 16S rRNA derived indicative OTUs with their taxonomic affiliations. Log2foldChange (l2FC) values are from paired comparisons between pure control soils (clay or silt) with four different bark-derived amendments (B, BH, BA, BHA). Results are shown for the clay and silt soil, and for the 2nd and 3rd samplings separately. Positive values refer to OTUs indicative for controls soils and negative for the microcosms with organic amendments. Abbreviations: C, control; B, bark, BH, hot water extracted bark; BA, bark from anaerobic digestion process; BHA, hot water extracted bark from anaerobic digestion; Aci, Acidobacteria; Act, Actinobacteria; Arm, Armatimonadetes; Atr, Atribacteria; Chla, Chlamydiae; Chlo, Chloroflexi; Clo, Cloacimonetes; Cya, Cyanobacteria; Dep, Dependentiae; Elu, Elusimicrobia Bact, Bacteroidetes; Fib, Fibrobacteres; Fir, Firmicutes; Gem, Gemmatimonadetes; Pat, Patescibacteria; Pla, Planctomycetes; Pro, Proteobacteiria; Syn, Synergistetes; Ver, Verrucomicrobia. Bolded texts are represented in the heatmap Figure 3.

2^nd^ sampling OTU_ID l2FC *p* adj Phylum/Class Order Family Genus

^________________________________________________________________________________________________________________________________________________________________________________________________________^

clay

C vs B **Otu00747 -3.3 4.E-04 Pro/Gammaproteobacteria Betaproteobacteriales *Burkholderiaceae* *Burkholderia***

**Otu00790 -5.2 3.E-16 Bact/Bacteroidia Sphingobacteriales *Sphingobacteriaceae Mucilaginibacter***

**Otu01226 -8.2 1.E-05 Ver/Verrucomicrobiae Verrucomicrobiales *Rubritaleaceae Luteolibacter***

**Otu03138 -21.1 6.E-07 Bact/Bacteroidia Cytophagales *Cytophagaceae Cytophaga***

^_______________________________________________________________________________________________________________________________________________________________________________________________________^

silt

C vs B **Otu01244 -3.0 1.7E-07 Ver/Verrucomicrobiae Chthoniobacterales *Chthoniobacteraceae Chthoniobacter***

**Otu00817 -3.1 7.0E-06 Act/Acidimicrobiia Microtrichales *Ilumatobacteraceae* unknown**

**Otu00640 -3.5 2.2E-09 Ver/Verrucomicrobiae Verrucomicrobiales *Verrucomicrobiaceae Verrucomicrobium***

**Otu05170 -5.2 4.1E-04 Pro/Alphaproteobacteria Caulobacterales Caulobacteraceae unknown**

**Otu03157 -5.4 4.5E-04 Act/Actinobacteria Corynebacteriales *Nocardiaceae Rhodococcus***

Otu00447 -6.1 6.8E-04 Pat/Saccharimonadia Saccharimonadales unknown unknown

**Otu05852 -6.4 1.8E-04 Bact/Bacteroidia Chitinophagales *Chitinophagaceae Niastella***

Otu03129 -6.5 6.1E-05 Act/Actinobacteria unknown unknown unknown

Otu03006 -6.7 2.8E-05 Ver/Verrucomicrobiae unknown unknown unknown

^________________________________________________________________________________________________________________________________________________________________________________________________________^

clay

C vs BH **Otu00747 -3.5 4.E-04 Pro/Gammaproteobacteria Betaproteobacteriales *Burkholderiaceae*** ***Burkholderia^1^*^)^**

**Otu01080 -3.6 1.E-03 Pro/Gammaproteobacteria Xanthomonadales *Rhodanobacteraceae Rhodanobacter***

**Otu00897 -4.0 6.E-04 Pro/Alphaproteobacteria Rhizobiales *Rhizobiaceae Allorhizobium^2)^***

**Otu00790 -5.5 3.E-15 Bact/Bacteroidia Sphingobacteriales *Sphingobacteriaceae Mucilaginibacter***

**Otu02429 -6.0 6.E-04 Pro/Alphaproteobacteria Sphingomonadales *Sphingomonadaceae Novosphingobium***

**Otu00766 -6.1 1.E-04 Pro/Gammaproteobacteria Xanthomonadales *Rhodanobacteraceae Rhodanobacter***

**Otu02157 -7.3 3.E-05 Pro/Gammaproteobacteria Enterobacteriales *Enterobacteriaceae Serratia***

**Otu00780 -7.5 1.E-05 Pro/Alphaproteobacteria Sphingomonadales *Sphingomonadaceae Sphingomonas***

**Otu01226 -8.6 5.E-05 Ver/Verrucomicrobiae Verrucomicrobiales *Rubritaleaceae Luteolibacter***

^________________________________________________________________________________________________________________________________________________________________________________________________________^

silt

C vs BH **Otu00897 -2.4 4.E-12 Pro/Alphaproteobacteria Rhizobiales *Rhizobiaceae Allorhizobium^2^*^)^**

**Otu00640 -2.8 3.E-04 Ver/Verrucomicrobiae Verrucomicrobiales *Verrucomicrobiaceae Verrucomicrobium***

**Otu01348 -3.6 2.E-08 Pro/Alphaproteobacteria Caulobacterales *Caulobacteraceae Caulobacter***

**Otu00790 -3.8 1.E-05 Bact/Bacteroidia Sphingobacteriales *Sphingobacteriaceae Mucilaginibacter***

**Otu03508 -4.4 5.E-04 Pro/Alphaproteobacteria Sphingomonadales *Sphingomonadaceae Novosphingobium***

**Otu01226 -5.5 1.E-05 Ver/Verrucomicrobiae Verrucomicrobiales *Rubritaleaceae Luteolibacter***

**Otu00766 -5.8 9.E-05 Pro/Gammaproteobacteria Xanthomonadales *Rhodanobacteraceae Rhodanobacter***

**Otu03260 -6.7 9.E-04 Bact/Bacteroidia Chitinophagales *Chitinophagaceae Filimonas***

Otu03006 -6.7 3.E-04 Ver/Verrucomicrobiae unknown unknown unknown

**Otu03428 -6.7 3.E-04 Bact/Bacteroidia Sphingobacteriales *Sphingobacteriaceae Mucilaginibacter***

**Otu01237 -6.7 6.E-04 Bact/Bacteroidia Flavobacteriales *Flavobacteriaceae Flavobacterium***

^________________________________________________________________________________________________________________________________________________________________________________________________________^

clay

C vs BA Otu02251 4.4 1.E-04 Pro/Deltaproteobacteria Myxococcales unknown unknown

Otu02833 3.2 6.E-07 Pro/Deltaproteobacteria Myxococcales unknown unknown

Otu00754 2.0 3.E-06 Pla/Planctomycetacia Planctomycetales unknown unknown

Otu01195 -2.1 1.E-06 Pro/Alphaproteobacteria Sphingomonadales ***Sphingomonadaceae Altererythrobacter***

Otu00938 -2.3 6.E-08 Pro/Gammaproteobacteria Xanthomonadales *Xanthomonadaceae Luteimonas*

Otu00738 -2.4 5.E-05 Pro/Deltaproteobacteria Myxococcales Sandaracinaceae unknown

Otu04474 -2.5 7.E-04 Pro/Alphaproteobacteria Rhizobiales *Xanthobacteraceae Rhodopseudomonas*

**Otu00450 -3.1 5.E-19 Pro/Alphaproteobacteria Rhizobiales *Devosiaceae Devosia***

**Otu00986 -3.1 4.E-31 Pro/Gammaproteobacteria Betaproteobacteriales *Nitrosomonadaceae Nitrosospira***

Otu00948 -3.2 2.E-04 Pro/Alphaproteobacteria Caulobacterales *Caulobacteraceae Phenylobacterium*

Otu00701 -3.3 2.E-08 Pro/Gammaproteobacteria Xanthomonadales *Xanthomonadaceae Lysobacter*

**Otu01046 -3.3 2.E-20 Pro/Gammaproteobacteria Pseudomonadales *Pseudomonadaceae Pseudomonas***

Otu00766 -3.9 2.E-04 Pro/Gammaproteobacteria Xanthomonadales *Rhodanobacteraceae Rhodanobacter*

**Otu00690 -4.0 1.E-11 Bact/Bacteroidia Cytophagales *Hymenobacteraceae Adhaeribacter***

Otu01348 -4.0 2.E-05 Pro/Alphaproteobacteria Caulobacterales *Caulobacteraceae Caulobacter*

Otu02482 -5.4 7.E-04 Pro/Alphaproteobacteria Micropepsales *Micropepsaceae* unknown

Otu01895 -5.5 7.E-04 Act/Acidimicrobiia Microtrichales *Microtrichaceae* unknown

Otu07072 -5.6 6.E-04 Syn/Synergistia Synergistales *Synergistaceae Thermovirga*

**Otu01208 -5.6 6.E-19 Fir/Clostridia Clostridiales *Peptostreptococcaceae Romboutsia***

Otu03991 -5.8 4.E-04 Act/Actinobacteria Frankiales *Nakamurellaceae Nakamurella*

Otu01075 -5.9 2.E-04 Fir/Clostridia Clostridiales *Ruminococcaceae Fastidiosipila*

Otu02436 -5.9 4.E-04 Fib/Fibrobacteria Fibrobacterales *Fibrobacteraceae* unknown

**Otu01161 -6.0 1.E-14 Act/Actinobacteria Frankiales *Cryptosporangiaceae Fodinicola***

Otu02001 -6.0 2.E-04 Fir/Clostridia Clostridiales *Clostridiaceae_1 Clostridium*

Otu05467 -6.0 3.E-08 Fir/Clostridia Clostridiales *Peptostreptococcaceae Eubacterium_tenue*

Otu05448 -6.0 10.E-05 Pla/Phycisphaerae Tepidisphaerales unknown unknown

Otu03049 -6.1 2.E-04 Pro/Alphaproteobacteria Sphingomonadales *Sphingomonadaceae Novosphingobium*

Otu03712 -6.2 2.E-04 Pro/Alphaproteobacteria Sphingomonadales *Sphingomonadaceae Altererythrobacter*

Otu01995 -6.2 4.E-04 Pro/Gammaproteobacteria Betaproteobacteriales *Burkholderiaceae Herminiimonas*

Otu07019 -6.2 10.E-04 Pla/Planctomycetacia Pirellulales *Pirellulaceae* unknown

Otu01088 -6.3 1.E-04 Fir/Bacilli Bacillales *Paenibacillaceae Paenibacillus*

Otu00383 -6.3 6.E-05 Pat/Saccharimonadia Saccharimonadales *Saccharimonadaceae* unknown

Otu01297 -6.4 1.E-04 Fir/Clostridia Clostridiales *Christensenellaceae* unknown

Otu05174 -6.4 3.E-05 Pro/Gammaproteobacteria Legionellales Legionellaceae Legionella

Otu00512 -6.4 2.E-04 Pat/Saccharimonadia Saccharimonadales *Saccharimonadaceae Cand_Saccharimonas*

Otu02419 -6.4 5.E-07 Ver/Verrucomicrobiae Verrucomicrobiales *Verrucomicrobiaceae Prosthecobacter*

Otu05034 -6.4 2.E-05 Ver/Verrucomicrobiae Pedosphaerales *Pedosphaeraceae* unknown

Otu09878 -6.4 1.E-04 Fir/Bacilli Bacillales *Paenibacillaceae Paenibacillus*

Otu01620 -6.6 2.E-07 Fir/Erysipelotrichia Erysipelotrichales *Erysipelotrichaceae Turicibacter*

Otu03722 -6.7 3.E-05 Aci/unknown unknown unknown unknown

Otu02431 -6.7 6.E-05 Atr/unknown unknown unknown unknown

Otu04395 -6.7 2.E-04 Fir/Clostridia Clostridiales *Christensenellaceae* unknown

**Otu01080 -6.7 2.E-09 Pro/Gammaproteobacteria Xanthomonadales *Rhodanobacteraceae Rhodanobacter***

Otu00428 -6.8 1.E-04 Pat/Saccharimonadia Saccharimonadales unknown unknown

Otu03687 -6.8 2.E-04 Pro/Deltaproteobacteria Myxococcales *Polyangiaceae Sorangium*

Otu02314 -6.9 2.E-06 Pro/Alphaproteobacteria unknown unknown unknown

Otu03979 -7.0 6.E-05 Pro/Alphaproteobacteria Sphingomonadales *Sphingomonadaceae Sphingopyxis*

Otu01989 -7.0 3.E-06 Pro/Gammaproteobacteria Betaproteobacteriales *Burkholderiaceae Advenella*

Otu05065 -7.0 3.E-05 Fir/Clostridia Clostridiales unknown *Sedimentibacter*

Otu01176 -7.1 2.E-05 Fir/Clostridia Clostridiales *Christensenellaceae* unknown

Otu05052 -7.3 2.E-06 Bact/Bacteroidia Bacteroidales unknown unknown

**Otu00514 -7.3 2.E-20 Bact/Bacteroidia Chitinophagales *Chitinophagaceae* unknown**

Otu07178 -7.5 3.E-07 Fir/Erysipelotrichia Erysipelotrichales *Erysipelotrichaceae Solobacterium*

Otu00516 -7.5 2.E-06 Pat/Saccharimonadia Saccharimonadales unknown unknown

Otu01204 -7.6 3.E-07 Pro/Alphaproteobacteria Caulobacterales *Caulobacteraceae Asticcacaulis*

Otu01115 -7.8 1.E-04 Bact/Bacteroidia Bacteroidales *Dysgonomonadaceae Fermentimonas*

Otu02427 -7.8 4.E-08 Bact/Bacteroidia Bacteroidales *Rikenellaceae* unknown

Otu00589 -7.8 4.E-07 Chl/Anaerolineae unknown unknown unknown

Otu01854 -8.0 8.E-08 Bact/Bacteroidia unknown unknown unknown

Otu00839 -8.2 2.E-08 Bact/Bacteroidia Bacteroidales unknown unknown

Otu00610 -8.3 6.E-09 Pro/Deltaproteobacteria Myxococcales *Nannocystaceae* unknown

Otu02306 -8.3 5.E-05 Fir/Clostridia Clostridiales *Ruminococcaceae Fastidiosipila*

Otu02413 -8.4 2.E-08 Pro/Alphaproteobacteria Micropepsales *Micropepsaceae* unknown

**Otu00734 -8.5 2.E-09 Ver/Verrucomicrobiae Chthoniobacterales *Chthoniobacteraceae* unknown**

Otu01081 -8.6 4.E-09 Pro/Gammaproteobacteria Xanthomonadales *Rhodanobacteraceae Rhodanobacter*

Otu02392 -9.4 5.E-06 Clo/Cloacimonadia Cloacimonadales unknown unknown

**Otu00733 -11.1 2.E-16 Chl/Anaerolineae Anaerolineales *Anaerolineaceae* unknown**

^________________________________________________________________________________________________________________________________________________________________________________________________________^

silt

C vs BA Otu03483 6.0 4.E-06 Aci/Acidobacteriia Solibacterales *Solibacteraceae*_Sb3 *Paludibaculum*

Otu03068 4.9 4.E-04 Ver/Verrucomicrobiae Chthoniobacterales *Chthoniobacteraceae Chthoniobacter*

Otu01210 4.3 9.E-07 Pro/Deltaproteobacteria Myxococcales *Haliangiaceae Haliangium*

Otu03280 4.1 5.E-04 Ver/Verrucomicrobiae Pedosphaerales *Pedosphaeraceae* unknown

**Otu05695 3.8 4.E-10 Pro/Gammaproteobacteria Betaproteobacteriales *Nitrosomonadaceae* unknown**

Otu00918 3.6 3.E-07 Chla/Chlamydiae Chlamydiales *Parachlamydiaceae* unknown

Otu01056 3.4 2.E-09 Pro/Gammaproteobacteria Betaproteobacteriales *Nitrosomonadaceae* unknown

Otu03116 3.3 1.E-05 Aci/unknown unknown unknown unknown

Otu02762 3.0 8.E-04 Elu/unknown unknown unknown unknown

Otu04862 2.7 8.E-04 Gem/Gemmatimonadetes Gemmatimonadales *Gemmatimonadaceae* unknown

Otu02144 2.6 1.E-05 Ver/Verrucomicrobiae Verrucomicrobiales *Verrucomicrobiaceae* unknown

Otu01347 2.6 5.E-04 Aci/Acidobacteriia Solibacterales *Solibacteraceae*_Sb3 *Bryobacter*

Otu03356 2.5 3.E-05 Bact/Bacteroidia Cytophagales *Microscillaceae* unknown

Otu05743 2.5 9.E-04 Pro/Deltaproteobacteria Myxococcales *Sandaracinaceae* unknown

Otu00445 2.4 1.E-05 Act/Acidimicrobiia Microtrichales *Ilumatobacteraceae* unknown

Otu02514 2.4 8.E-06 Pro/Gammaproteobacteria Betaproteobacteriales unknown unknown

Otu02133 -2.1 2.E-10 Pro/Gammaproteobacteria Betaproteobacteriales unknown unknown

**Otu00450 -2.2 6.E-19 Pro/Alphaproteobacteria Rhizobiales *Devosiaceae Devosia***

**Otu01024 -2.3 5.E-10 Pro/Alphaproteobacteria Rhizobiales *Rhizobiaceae Mesorhizobium***

Otu01788 -2.3 3.E-04 Pla/Planctomycetacia Pirellulales *Pirellulaceae* unknown

Otu01522 -2.4 5.E-08 Pla/Planctomycetacia Isosphaerales *Isosphaeraceae* unknown

Otu03715 -2.5 5.E-04 Pla/Planctomycetacia Pirellulales *Pirellulaceae* unknown

Otu03969 -2.5 8.E-05 Pla/Planctomycetacia Pirellulales *Pirellulaceae* unknown

Otu01577 -2.5 3.E-04 Pla/Planctomycetacia Isosphaerales *Isosphaeraceae* unknown

Otu03890 -2.5 3.E-04 Dep/Babeliae Babeliales *Vermiphilaceae* unknown

Otu00927 -2.6 2.E-05 Bact/Bacteroidia Flavobacteriales *Flavobacteriaceae Flavobacterium*

Otu02152 -2.6 5.E-04 Bact/Bacteroidia Sphingobacteriales *Sphingobacteriaceae Mucilaginibacter*

**Otu00986 -2.7 2.E-30 Pro/Gammaproteobacteria Betaproteobacteriales *Nitrosomonadaceae Nitrosospira***

Otu00734 -2.7 1.E-04 Ver/Verrucomicrobiae Chthoniobacterales *Chthoniobacteraceae* unknown

Otu02072 -2.7 3.E-04 Pla/Planctomycetacia Isosphaerales *Isosphaeraceae Aquisphaera*

**Otu01161 -2.8 1.E-13 Act/Actinobacteria Frankiales *Cryptosporangiaceae Fodinicola***

Otu00938 -2.8 9.E-10 Pro/Gammaproteobacteria Xanthomonadales *Xanthomonadaceae Luteimonas*

**Otu00933 -2.9 3.E-30 Pro/Gammaproteobacteria Betaproteobacteriales *Burkholderiaceae Polaromonas***

Otu03676 -2.9 2.E-05 Pro/Gammaproteobacteria unknown unknown unknown

Otu01178 -3.0 2.E-06 Pla/Planctomycetacia Planctomycetales *Rubinisphaeraceae* unknown

Otu03090 -3.0 1.E-07 Pla/Planctomycetacia Pirellulales *Pirellulaceae Pirellula*

**Otu01173 -3.1 5.E-17 Pla/Planctomycetacia Pirellulales *Pirellulaceae Pirellula***

Otu01513 -3.1 5.E-05 Bact/Bacteroidia Chitinophagales *Saprospiraceae* unknown

Otu01195 -3.2 4.E-04 Pro/Alphaproteobacteria Sphingomonadales *Sphingomonadaceae Altererythrobacter*

**Otu02429 -3.3 1.E-14 Pro/Alphaproteobacteria Sphingomonadales *Sphingomonadaceae Novosphingobium***

Otu00962 -3.4 2.E-07 Pro/Gammaproteobacteria Xanthomonadales *Xanthomonadaceae Luteimonas*

Otu03612 -3.4 1.E-05 Pro/Alphaproteobacteria Rhizobiales *Devosiaceae Devosia*

Otu00798 -3.4 6.E-04 Pro/Gammaproteobacteria Xanthomonadales *Xanthomonadaceae Lysobacter*

Otu00863 -3.5 7.E-09 Pro/Alphaproteobacteria Caulobacterales *Caulobacteraceae Brevundimonas*

Otu01567 -3.6 3.E-04 Gem/Gemmatimonadetes Gemmatimonadales *Gemmatimonadaceae Gemmatimonas*

Otu00720 -3.7 1.E-09 Bact/Bacteroidia Chitinophagales *Chitinophagaceae* unknown

Otu03682 -3.7 1.E-05 Pro/Alphaproteobacteria Sphingomonadales *Sphingomonadaceae Novosphingobium*

Otu00982 -3.8 6.E-08 Chlo/unknown unknown unknown unknown

Otu02718 -3.8 4.E-07 Pla/Planctomycetacia Pirellulales *Pirellulaceae Pirellula*

Otu00600 -3.9 5.E-07 Pla/Phycisphaerae Phycisphaerales *Phycisphaeraceae* unknown

Otu00638 -4.0 3.E-05 Pla/Planctomycetacia Planctomycetales *Rubinisphaeraceae* unknown

Otu01948 -4.2 3.E-09 Pro/Alphaproteobacteria Rhizobiales *Devosiaceae Devosia*

Otu01995 -4.3 4.E-06 Pro/Gammaproteobacteria Betaproteobacteriales *Burkholderiaceae Herminiimonas*

Otu07475 -4.6 7.E-04 Pro/Alphaproteobacteria Rhizobiales *Xanthobacteraceae Pseudolabrys*

Otu05366 -5.2 9.E-04 Pro/Gammaproteobacteria Legionellales *Legionellaceae Legionella*

Otu00479 -5.3 5.E-05 Pat/Saccharimonadia Saccharimonadales unknown unknown

Otu07795 -5.4 5.E-04 Chla/Chlamydiae Chlamydiales Parachlamydiaceae unknown

Otu00512 -5.4 5.E-04 Pat/Saccharimonadia Saccharimonadales *Saccharimonadaceae Cand_Saccharimonas*

Otu00516 -5.5 9.E-04 Pat/Saccharimonadia Saccharimonadales unknown unknown

Otu03302 -5.5 2.E-04 Pla/Planctomycetacia Pirellulales *Pirellulaceae Pirellula*

Otu01989 -5.7 5.E-04 Pro/Gammaproteobacteria Betaproteobacteriales *Burkholderiaceae Advenella*

Otu02089 -5.7 1.E-04 Cya/Sericytochromatia unknown unknown unknown

Otu01297 -5.7 5.E-04 Fir/Clostridia Clostridiales *Christensenellaceae* unknown

Otu06889 -5.9 5.E-04 Gem/Gemmatimonadetes Gemmatimonadales *Gemmatimonadaceae* unknown

Otu00797 -6.0 4.E-05 Pro/Alphaproteobacteria Sphingomonadales *Sphingomonadaceae Sphingobium*

Otu04078 -6.0 2.E-04 Chlo/Anaerolineae Caldilineales *Caldilineaceae* unknown

Otu03979 -6.0 5.E-05 Pro/Alphaproteobacteria Sphingomonadales *Sphingomonadaceae Sphingopyxis*

Otu01883 -6.0 7.E-05 Fir/Clostridia Clostridiales *Christensenellaceae* unknown

Otu04395 -6.1 3.E-04 Fir/Clostridia Clostridiales *Christensenellaceae* unknown

Otu02431 -6.1 7.E-05 Atr/unknown unknown unknown unknown

Otu03736 -6.1 4.E-05 Pro/Gammaproteobacteria Pseudomonadales unknown unknown

Otu07358 -6.1 3.E-04 Fir/Clostridia Clostridiales unknown unknown

Otu07019 -6.2 4.E-05 Pla/Planctomycetacia Pirellulales *Pirellulaceae* unknown

Otu03696 -6.2 3.E-05 Fir/Clostridia Clostridiales *Peptococcaceae Desulfitibacter*

Otu03673 -6.2 8.E-05 Pro/Alphaproteobacteria Parvibaculales *Parvibaculaceae Parvibaculum*

Otu07068 -6.2 4.E-04 Ver/Verrucomicrobiae Opitutales *Opitutaceae Lacunisphaera*

Otu03992 -6.3 1.E-05 Pro/Alphaproteobacteria Micavibrionales unknown unknown

Otu06886 -6.3 8.E-05 Pro/Deltaproteobacteria Myxococcales *Vulgatibacteraceae Vulgatibacter*

Otu06852 -6.3 4.E-05 Pro/Gammaproteobacteria Xanthomonadales Xanthomonadaceae Pseudoxanthomonas

Otu00936 -6.3 6.E-05 Pro/Alphaproteobacteria Caulobacterales *Caulobacteraceae Brevundimonas*

Otu05065 -6.3 5.E-05 Fir/Clostridia Clostridiales unknown *Sedimentibacter*

Otu05377 -6.3 5.E-05 Pro/Alphaproteobacteria Caulobacterales *Caulobacteraceae Caulobacter*

Otu03722 -6.4 4.E-05 Aci/unknown unknown unknown unknown

Otu03988 -6.5 1.E-05 Bact/Bacteroidia Chitinophagales *Chitinophagaceae Ferruginibacter*

Otu07082 -6.5 2.E-04 Pla/Planctomycetacia Planctomycetales unknown unknown

Otu07381 -6.5 5.E-05 Pro/Gammaproteobacteria Pseudomonadales unknown *Psychrobacter*

Otu03687 -6.5 7.E-06 Pro/Deltaproteobacteria Myxococcales *Polyangiaceae Sorangium*

Otu07178 -6.6 7.E-06 Fir/Erysipelotrichia Erysipelotrichales *Erysipelotrichaceae Solobacterium*

Otu03593 -6.7 2.E-05 Pro/Gammaproteobacteria Betaproteobacteriales *Burkholderiaceae Limnobacter*

Otu03546 -6.8 1.E-06 Bact/Bacteroidia Flavobacteriales unknown unknown

Otu01176 -6.9 9.E-06 Fir/Clostridia Clostridiales *Christensenellaceae* unknown

Otu01075 -7.0 4.E-06 Fir/Clostridia Clostridiales *Ruminococcaceae Fastidiosipila*

Otu01847 -7.0 4.E-07 Bact/Bacteroidia Flavobacteriales *Flavobacteriaceae Flavobacterium*

Otu03675 -7.0 1.E-06 Pro/Alphaproteobacteria Rhizobiales *Beijerinckiaceae* unknown

Otu01886 -7.1 2.E-06 Bact/Bacteroidia Cytophagales *Microscillaceae* unknown

Otu01080 -7.2 3.E-06 Pro/Gammaproteobacteria Xanthomonadales *Rhodanobacteraceae Rhodanobacter*

Otu03640 -7.2 8.E-04 Pro/Alphaproteobacteria Rhizobiales *Devosiaceae Devosia*

Otu00514 -7.2 1.E-09 Bact/Bacteroidia Chitinophagales *Chitinophagaceae* unknown

Otu05052 -7.2 1.E-06 Bact/Bacteroidia Bacteroidales unknown unknown

Otu03703 -7.3 5.E-05 Pro/Deltaproteobacteria Myxococcales *Sandaracinaceae* unknown

Otu00707 -7.4 8.E-08 Bact/Bacteroidia Flavobacteriales *Weeksellaceae Chryseobacterium*

Otu01854 -7.5 6.E-07 Bact/Bacteroidia unknown unknown unknown

Otu00589 -7.5 1.E-07 Chlo/Anaerolineae unknown unknown unknown

Otu01115 -7.5 2.E-07 Bact/Bacteroidia Bacteroidales *Dysgonomonadaceae Fermentimonas*

Otu04124 -7.5 1.E-07 Pla/Planctomycetacia Planctomycetales *Schlesneriaceae Planctopirus*

Otu02427 -7.6 1.E-07 Bact/Bacteroidia Bacteroidales *Rikenellaceae* unknown

Otu03691 -7.6 7.E-08 Bact/Bacteroidia Chitinophagales *Chitinophagaceae Ferruginibacter*

Otu02306 -7.9 6.E-08 Fir/Clostridia Clostridiales *Ruminococcaceae Fastidiosipila*

Otu00839 -8.0 6.E-08 Bact/Bacteroidia Bacteroidales unknown unknown

Otu02392 -8.2 3.E-07 Clo/Cloacimonadia Cloacimonadales unknown unknown

**Otu00932 -9.3 3.E-10 Pro/Gammaproteobacteria Xanthomonadales *Xanthomonadaceae Pseudoxanthomonas***

**Otu00733 -9.8 4.E-13 Chlo/Anaerolineae Anaerolineales *Anaerolineaceae* unknown**

Otu02010 -22.0 2.E-08 Bact/Bacteroidia Bacteroidales *Marinilabiliaceae* unknown

^________________________________________________________________________________________________________________________________________________________________________________________________________^

clay

C vs BHA Otu04554 4.4 4.E-05 Ver/Verrucomicrobiae Pedosphaerales *Pedosphaeraceae* unknown

Otu01056 4.2 4.E-05 Pro/Gammaproteobacteria Betaproteobacteriales *Nitrosomonadaceae* unknown

Otu02833 3.1 2.E-07 Pro/Deltaproteobacteria Myxococcales unknown unknown

Otu02251 2.9 2.E-04 Pro/Deltaproteobacteria Myxococcales unknown unknown

Otu01168 2.1 4.E-06 Pro/Deltaproteobacteria Myxococcales *Haliangiaceae Haliangium*

Otu00798 -2.1 7.E-09 Pro/Gammaproteobacteria Xanthomonadales *Xanthomonadaceae Lysobacter*

Otu01261 -2.2 9.E-04 Pro/Gammaproteobacteria Xanthomonadales *Xanthomonadaceae Luteimonas*

Otu02133 -2.3 2.E-07 Pro/Gammaproteobacteria Betaproteobacteriales unknown unknown

Otu02322 -2.4 2.E-07 Pro/Alphaproteobacteria Sphingomonadales *Sphingomonadaceae Sphingomonas*

Otu02159 -2.4 7.E-08 Pro/Gammaproteobacteria Xanthomonadales Rhodanobacteraceae Dokdonella

Otu00948 -2.5 2.E-04 Pro/Alphaproteobacteria Caulobacterales *Caulobacteraceae Phenylobacterium*

**Otu00938 -2.5 1.E-14 Pro/Gammaproteobacteria Xanthomonadales *Xanthomonadaceae Luteimonas***

Otu00738 -2.5 2.E-05 Pro/Deltaproteobacteria Myxococcales Sandaracinaceae unknown

Otu01195 -2.6 1.E-08 Pro/Alphaproteobacteria Sphingomonadales *Sphingomonadaceae Altererythrobacter*

Otu00957 -2.7 3.E-07 Pla/Phycisphaerae Tepidisphaerales unknown unknown

Otu01111 -2.8 6.E-05 Act/Acidimicrobiia Microtrichales *Microtrichaceae* unknown

Otu04036 -2.8 4.E-04 Arm/unknown unknown unknown unknown

Otu05233 -2.9 6.E-04 Arm/unknown unknown unknown unknown

Otu01860 -3.1 4.E-09 Bact/Bacteroidia Chitinophagales *Chitinophagaceae* unknown

Otu04474 -3.2 9.E-08 Pro/Alphaproteobacteria Rhizobiales *Xanthobacteraceae Rhodopseudomonas*

Otu00932 -3.3 1.E-03 Pro/Gammaproteobacteria Xanthomonadales *Xanthomonadaceae Pseudoxanthomonas*

**Otu00701 -3.4 3.E-19 Pro/Gammaproteobacteria Xanthomonadales *Xanthomonadaceae Lysobacter***

**Otu00450 -3.4 3.E-22 Pro/Alphaproteobacteria Rhizobiales *Devosiaceae Devosia***

**Otu00986 -3.6 3.E-50 Pro/Gammaproteobacteria Betaproteobacteriales *Nitrosomonadaceae Nitrosospira***

Otu00690 -3.7 1.E-10 Bact/Bacteroidia Cytophagales *Hymenobacteraceae Adhaeribacter*

Otu00816 -3.7 5.E-06 Ver/Verrucomicrobiae Opitutales *Opitutaceae Lacunisphaera*

Otu01348 -3.7 5.E-06 Pro/Alphaproteobacteria Caulobacterales *Caulobacteraceae Caulobacter*

Otu00766 -3.8 2.E-06 Pro/Gammaproteobacteria Xanthomonadales *Rhodanobacteraceae Rhodanobacter*

Otu01046 -3.8 1.E-07 Pro/Gammaproteobacteria Pseudomonadales *Pseudomonadaceae Pseudomonas*

Otu00914 -4.1 9.E-04 Pro/Gammaproteobacteria Xanthomonadales *Rhodanobacteraceae Dokdonella*

Otu03139 -4.1 8.E-04 Pro/Alphaproteobacteria Caulobacterales *Caulobacteraceae Asticcacaulis*

Otu01995 -4.2 3.E-04 Pro/Gammaproteobacteria Betaproteobacteriales *Burkholderiaceae Herminiimonas*

Otu00797 -5.1 2.E-04 Pro/Alphaproteobacteria Sphingomonadales *Sphingomonadaceae Sphingobium*

Otu02429 -5.2 8.E-04 Pro/Alphaproteobacteria Sphingomonadales *Sphingomonadaceae Novosphingobium*

Otu00482 -5.4 1.E-03 Pat/Saccharimonadia Saccharimonadales unknown unknown

**Otu01208 -5.4 2.E-25 Fir/Clostridia Clostridiales *Peptostreptococcaceae Romboutsia***

Otu07072 -5.5 5.E-04 Syn/Synergistia Synergistales *Synergistaceae Thermovirga*

Otu00673 -5.5 4.E-04 Chlo/Anaerolineae unknown unknown unknown

Otu01228 -5.5 1.E-04 Act/Actinobacteria Micrococcales *Microbacteriaceae Galbitalea*

Otu04126 -5.5 2.E-04 Gem/Gemmatimonadetes Gemmatimonadales *Gemmatimonadaceae Gemmatimonas*

Otu02433 -5.5 4.E-04 Pla/Planctomycetacia Gemmatales *Gemmataceae Gemmata*

Otu03550 -5.5 3.E-04 Pro/Deltaproteobacteria Myxococcales Sandaracinaceae unknown

Otu04955 -5.6 4.E-04 Ver/Verrucomicrobiae Chthoniobacterales *Terrimicrobiaceae Terrimicrobium*

Otu01297 -5.6 3.E-04 Fir/Clostridia Clostridiales Christensenellaceae unknown

Otu01974 -5.7 9.E-04 Bact/Bacteroidia Flavobacteriales *Flavobacteriaceae Flavobacterium*

Otu05065 -5.7 2.E-04 Fir/Clostridia Clostridiales unknown Sedimentibacter

Otu00512 -5.8 2.E-04 Pat/Saccharimonadia Saccharimonadales *Saccharimonadaceae Cand_Saccharimonas*

Otu01341 -5.8 1.E-04 Fir/Bacilli Bacillales *Paenibacillaceae Paenibacillus*

Otu05448 -5.8 2.E-04 Pla/Phycisphaerae Tepidisphaerales unknown unknown

Otu03428 -5.8 2.E-04 Bact/Bacteroidia Sphingobacteriales *Sphingobacteriaceae Mucilaginibacter*

Otu03049 -5.8 2.E-04 Pro/Alphaproteobacteria Sphingomonadales *Sphingomonadaceae Novosphingobium*

Otu05161 -5.8 3.E-04 Bact/Bacteroidia Bacteroidales Rikenellaceae unknown

Otu05467 -5.8 3.E-09 Fir/Clostridia Clostridiales *Peptostreptococcaceae Eubacterium_tenue*

Otu01895 -5.8 2.E-04 Act/Acidimicrobiia Microtrichales Microtrichaceae unknown

Otu08977 -5.9 2.E-04 Pro/Gammaproteobacteria unknown unknown unknown

Otu03991 -5.9 3.E-04 Act/Actinobacteria Frankiales *Nakamurellaceae Nakamurella*

Otu02431 -5.9 1.E-04 Atr/unknown unknown unknown unknown

Otu04395 -5.9 2.E-04 Fir/Clostridia Clostridiales Christensenellaceae unknown

Otu02001 -6.0 7.E-05 Fir/Clostridia Clostridiales *Clostridiaceae Clostridium*_ss_1

Otu00652 -6.0 8.E-05 Bact/Bacteroidia Chitinophagales Chitinophagaceae unknown

**Otu01080 -6.0 2.E-32 Pro/Gammaproteobacteria Xanthomonadales *Rhodanobacteraceae Rhodanobacter***

Otu02482 -6.1 3.E-05 Pro/Alphaproteobacteria Micropepsales Micropepsaceae unknown

**Otu01161 -6.1 1.E-15 Act/Actinobacteria Frankiales *Cryptosporangiaceae Fodinicola***

Otu05060 -6.1 8.E-05 Bact/Bacteroidia Bacteroidales *Dysgonomonadaceae Proteiniphilum*

Otu01088 -6.1 8.E-05 Fir/Bacilli Bacillales *Paenibacillaceae Paenibacillus*

Otu03722 -6.2 2.E-05 Aci/unknown unknown unknown unknown

Otu05256 -6.3 2.E-04 Pla/Phycisphaerae Tepidisphaerales unknown unknown

Otu01176 -6.4 2.E-05 Fir/Clostridia Clostridiales Christensenellaceae unknown

Otu02419 -6.4 2.E-07 Ver/Verrucomicrobiae Verrucomicrobiales *Verrucomicrobiaceae Prosthecobacter*

Otu01560 -6.4 3.E-05 Pat/Microgenomatia unknown unknown unknown

Otu01620 -6.4 9.E-08 Fir/Erysipelotrichia Erysipelotrichales *Erysipelotrichaceae Turicibacter*

Otu05174 -6.5 9.E-06 Pro/Gammaproteobacteria Legionellales *Legionellaceae Legionella*

Otu05385 -6.5 6.E-05 Pro/Gammaproteobacteria Pseudomonadales *Pseudomonadaceae Pseudomonas*

Otu08719 -6.5 2.E-05 Pro/Deltaproteobacteria Bdellovibrionales *Bdellovibrionaceae Bdellovibrio*

Otu09878 -6.5 7.E-06 Fir/Bacilli Bacillales *Paenibacillaceae Paenibacillus*

Otu02436 -6.6 2.E-05 Fib/Fibrobacteria Fibrobacterales Fibrobacteraceae unknown

Otu03712 -6.8 5.E-06 Pro/Alphaproteobacteria Sphingomonadales *Sphingomonadaceae Altererythrobacter*

Otu01115 -6.9 2.E-06 Bact/Bacteroidia Bacteroidales *Dysgonomonadaceae Fermentimonas*

Otu05034 -6.9 1.E-06 Ver/Verrucomicrobiae Pedosphaerales *Pedosphaeraceae* unknown

Otu00428 -7.0 2.E-06 Pat/Saccharimonadia Saccharimonadales unknown unknown

Otu00383 -7.0 4.E-06 Pat/Saccharimonadia Saccharimonadales *Saccharimonadaceae* unknown

Otu02306 -7.3 3.E-07 Fir/Clostridia Clostridiales *Ruminococcaceae Fastidiosipila*

Otu02314 -7.3 3.E-07 Pro/Alphaproteobacteria unknown unknown unknown

Otu05052 -7.3 2.E-07 Bact/Bacteroidia Bacteroidales unknown unknown

**Otu00514 -7.4 3.E-23 Bact/Bacteroidia Chitinophagales *Chitinophagaceae* unknown**

Otu07178 -7.4 8.E-08 Fir/Erysipelotrichia Erysipelotrichales *Erysipelotrichaceae Solobacterium*

Otu00936 -7.6 1.E-07 Pro/Alphaproteobacteria Caulobacterales *Caulobacteraceae Brevundimonas*

Otu00589 -7.6 4.E-08 Chlo/Anaerolineae unknown unknown unknown

Otu00516 -7.7 3.E-08 Pat/Saccharimonadia Saccharimonadales unknown unknown

Otu01989 -7.8 2.E-07 Pro/Gammaproteobacteria Betaproteobacteriales *Burkholderiaceae Advenella*

Otu00610 -7.8 2.E-06 Pro/Deltaproteobacteria Myxococcales *Nannocystaceae* unknown

Otu03979 -7.9 4.E-08 Pro/Alphaproteobacteria Sphingomonadales *Sphingomonadaceae Sphingopyxis*

Otu02427 -8.1 4.E-08 Bact/Bacteroidia Bacteroidales Rikenellaceae unknown

Otu00839 -8.2 6.E-09 Bact/Bacteroidia Bacteroidales unknown unknown

Otu01204 -8.2 2.E-06 Pro/Alphaproteobacteria Caulobacterales *Caulobacteraceae Asticcacaulis*

Otu01854 -8.2 7.E-09 Bact/Bacteroidia unknown unknown unknown

Otu02392 -8.2 5.E-09 Clo/Cloacimonadia Cloacimonadales unknown unknown

Otu00734 -8.7 1.E-10 Ver/Verrucomicrobiae Chthoniobacterales *Chthoniobacteraceae* unknown

Otu01081 -9.2 7.E-12 Pro/Gammaproteobacteria Xanthomonadales *Rhodanobacteraceae Rhodanobacter*

**Otu02413 -9.4 5.E-12 Pro/Alphaproteobacteria Micropepsales *Micropepsaceae* unknown**

**Otu00733 -10.7 1.E-16 Chlo/Anaerolineae Anaerolineales *Anaerolineaceae* unknown**

^________________________________________________________________________________________________________________________________________________________________________________________________________^

silt

C vs BHA Otu03483 5.4 3.E-05 Aci/Acidobacteriia Solibacterales *Solibacteraceae*_Sb3 Paludibaculum

Otu06440 5.4 3.E-05 Arm/Chthonomonadetes Chthonomonadales *Chthonomonadaceae Chthonomonas*

Otu01210 4.2 2.E-06 Pro/Deltaproteobacteria Myxococcales *Haliangiaceae Haliangium*

**Otu05695 3.7 4.E-10 Pro/Gammaproteobacteria Betaproteobacteriales Nitrosomonadaceae unknown**

Otu00918 3.7 1.E-06 Chla/Chlamydiae Chlamydiales *Parachlamydiaceae* unknown

Otu01234 3.5 3.E-04 Ver/Verrucomicrobiae Pedosphaerales *Pedosphaeraceae* unknown

Otu01056 3.5 7.E-09 Pro/Gammaproteobacteria Betaproteobacteriales *Nitrosomonadaceae* unknown

Otu05743 3.1 3.E-05 Pro/Deltaproteobacteria Myxococcales *Sandaracinaceae* unknown

Otu00445 3.1 3.E-06 Act/Acidimicrobiia Microtrichales *Ilumatobacteraceae* unknown

Otu03356 2.7 9.E-06 Bact/Bacteroidia Cytophagales *Microscillaceae* unknown

Otu03116 2.5 3.E-04 Aci/unknown unknown unknown unknown

Otu00610 2.4 2.E-04 Pro/Deltaproteobacteria Myxococcales *Nannocystaceae* unknown

Otu02514 2.3 5.E-06 Pro/Gammaproteobacteria Betaproteobacteriales unknown unknown

Otu02144 2.3 3.E-05 Ver/Verrucomicrobiae Verrucomicrobiales *Verrucomicrobiaceae* unknown

Otu01788 -2.1 8.E-04 Pla/Planctomycetacia Pirellulales *Pirellulaceae* unknown

Otu01522 -2.2 1.E-07 Pla/Planctomycetacia Isosphaerales *Isosphaeraceae* unknown

**Otu00450 -2.3 6.E-13 Pro/Alphaproteobacteria Rhizobiales *Devosiaceae Devosia***

Otu03969 -2.3 5.E-04 Pla/Planctomycetacia Pirellulales *Pirellulaceae* unknown

Otu01974 -2.3 8.E-04 Bact/Bacteroidia Flavobacteriales *Flavobacteriaceae Flavobacterium* Otu01348 -2.5 2.E-04 Pro/Alphaproteobacteria Caulobacterales *Caulobacteraceae Caulobacter*

**Otu00933 -2.7 4.E-16 Pro/Gammaproteobacteria Betaproteobacteriales *Burkholderiaceae Polaromonas***

**Otu00986 -2.7 1.E-17 Pro/Gammaproteobacteria Betaproteobacteriales *Nitrosomonadaceae Nitrosospira***

**Otu01161 -2.9 3.E-16 Act/Actinobacteria Frankiales *Cryptosporangiaceae Fodinicola***

Otu00734 -3.0 2.E-06 Ver/Verrucomicrobiae Chthoniobacterales *Chthoniobacteraceae* unknown

Otu03682 -3.0 1.E-04 Pro/Alphaproteobacteria Sphingomonadales *Sphingomonadaceae Novosphingobium*

Otu03890 -3.0 7.E-06 Dep/Babeliae Babeliales *Vermiphilaceae* unknown

**Otu00938 -3.1 1.E-14 Pro/Gammaproteobacteria Xanthomonadales *Xanthomonadaceae Luteimonas***

**Otu01173 -3.1 8.E-21 Pla/Planctomycetacia Pirellulales *Pirellulaceae Pirellula***

Otu03090 -3.1 1.E-08 Pla/Planctomycetacia Pirellulales *Pirellulaceae Pirellula*

Otu01178 -3.2 5.E-09 Pla/Planctomycetacia Planctomycetales *Rubinisphaeraceae* unknown

Otu01195 -3.3 2.E-04 Pro/Alphaproteobacteria Sphingomonadales Sphingomonadaceae Altererythrobacter

**Otu02429 -3.3 1.E-17 Pro/Alphaproteobacteria Sphingomonadales *Sphingomonadaceae Novosphingobium***

Otu01513 -3.3 2.E-04 Bact/Bacteroidia Chitinophagales *Saprospiraceae* unknown

Otu00982 -3.3 5.E-07 Chlo/unknown unknown unknown unknown

Otu00863 -3.3 3.E-08 Pro/Alphaproteobacteria Caulobacterales *Caulobacteraceae Brevundimonas*

**Otu00720 -3.5 9.E-13 Bact/Bacteroidia Chitinophagales *Chitinophagaceae* unknown**

Otu00962 -3.6 3.E-07 Pro/Gammaproteobacteria Xanthomonadales *Xanthomonadaceae Luteimonas*

Otu02718 -3.7 1.E-06 Pla/Planctomycetacia Pirellulales *Pirellulaceae Pirellula*

Otu01948 -3.8 2.E-06 Pro/Alphaproteobacteria Rhizobiales *Devosiaceae Devosia*

Otu00638 -3.8 7.E-05 Pla/Planctomycetacia Planctomycetales *Rubinisphaeraceae* unknown

Otu00479 -5.2 6.E-05 Pat/Saccharimonadia Saccharimonadales unknown unknown

Otu07019 -5.3 7.E-04 Pla/Planctomycetacia Pirellulales *Pirellulaceae* unknown

Otu04244 -5.6 4.E-04 Pat/Gracilibacteria Candidatus_Peribacteria unknown unknown

Otu02089 -5.6 2.E-04 Cya/Sericytochromatia unknown unknown unknown

Otu03722 -5.6 5.E-04 Aci/unknown unknown unknown unknown

Otu03302 -5.8 8.E-05 Pla/Planctomycetacia Pirellulales *Pirellulaceae Pirellula*

Otu07795 -5.8 3.E-04 Chla/Chlamydiae Chlamydiales *Parachlamydiaceae* unknown

Otu00707 -5.8 5.E-06 Bact/Bacteroidia Flavobacteriales *Weeksellaceae Chryseobacterium*

Otu07082 -5.8 2.E-04 Pla/Planctomycetacia Planctomycetales unknown unknown

Otu03640 -5.8 3.E-04 Pro/Alphaproteobacteria Rhizobiales *Devosiaceae Devosia*

Otu02002 -5.9 1.E-04 Pro/Alphaproteobacteria Caulobacterales Caulobacteraceae unknown

Otu03736 -5.9 2.E-04 Pro/Gammaproteobacteria Pseudomonadales unknown unknown

Otu01176 -5.9 2.E-04 Fir/Clostridia Clostridiales *Christensenellaceae* unknown

Otu01080 -5.9 1.E-04 Pro/Gammaproteobacteria Xanthomonadales *Rhodanobacteraceae Rhodanobacter*

Otu03696 -6.0 7.E-05 Fir/Clostridia Clostridiales *Peptococcaceae Desulfitibacter*

Otu03979 -6.0 6.E-04 Pro/Alphaproteobacteria Sphingomonadales *Sphingomonadaceae Sphingopyxis*

Otu04124 -6.0 7.E-05 Pla/Planctomycetacia Planctomycetales *Schlesneriaceae Planctopirus*

Otu07178 -6.1 9.E-05 Fir/Erysipelotrichia Erysipelotrichales *Erysipelotrichaceae Solobacterium*

Otu00797 -6.1 2.E-04 Pro/Alphaproteobacteria Sphingomonadales *Sphingomonadaceae Sphingobium*

Otu02306 -6.3 4.E-05 Fir/Clostridia Clostridiales *Ruminococcaceae Fastidiosipila*

Otu01847 -6.3 3.E-05 Bact/Bacteroidia Flavobacteriales *Flavobacteriaceae Flavobacterium*

Otu03691 -6.3 3.E-05 Bact/Bacteroidia Chitinophagales *Chitinophagaceae Ferruginibacter*

Otu03546 -6.4 2.E-05 Bact/Bacteroidia Flavobacteriales unknown unknown

Otu05052 -6.5 2.E-05 Bact/Bacteroidia Bacteroidales unknown unknown

Otu01989 -6.6 9.E-06 Pro/Gammaproteobacteria Betaproteobacteriales *Burkholderiaceae Advenella*

Otu00589 -6.6 2.E-05 Chlo/Anaerolineae unknown unknown unknown

Otu00514 -6.6 3.E-08 Bact/Bacteroidia Chitinophagales *Chitinophagaceae* unknown

Otu05377 -6.6 2.E-05 Pro/Alphaproteobacteria Caulobacterales *Caulobacteraceae Caulobacter*

Otu01886 -6.7 5.E-06 Bact/Bacteroidia Cytophagales *Microscillaceae* unknown

Otu02427 -6.7 9.E-06 Bact/Bacteroidia Bacteroidales *Rikenellaceae* unknown

Otu03992 -6.8 4.E-06 Pro/Alphaproteobacteria Micavibrionales unknown unknown

Otu01115 -6.9 5.E-06 Bact/Bacteroidia Bacteroidales *Dysgonomonadaceae Fermentimonas*

Otu02392 -6.9 5.E-06 Clo/Cloacimonadia Cloacimonadales unknown unknown

Otu03703 -7.3 4.E-05 Pro/Deltaproteobacteria Myxococcales *Sandaracinaceae* unknown

Otu01854 -7.3 8.E-05 Bact/Bacteroidia unknown unknown unknown

Otu00932 -7.5 9.E-08 Pro/Gammaproteobacteria Xanthomonadales *Xanthomonadaceae Pseudoxanthomonas*

Otu03675 -7.6 2.E-05 Pro/Alphaproteobacteria Rhizobiales *Beijerinckiaceae* unknown

Otu00839 -8.0 6.E-08 Bact/Bacteroidia Bacteroidales unknown unknown

**Otu00733 -9.1 7.E-12 Chlo/Anaerolineae Anaerolineales *Anaerolineaceae* unknown**

^________________________________________________________________________________________________________________________________________________________________________________________________________^

3^rd^ sampling

^________________________________________________________________________________________________________________________________________________________________________________________________________^

clay

C vs B Otu01688 3.0 2.E-04 Pro/Deltaproteobacteria Myxococcales unknown unknown

**Otu00710 -2.1 3.E-12 Ver/Verrucomicrobiae Chthoniobacterales *Chthoniobacteraceae Chthoniobacter***

Otu00903 -2.3 9.E-05 Pro/Gammaproteobacteria Pseudomonadales *Pseudomonadaceae Pseudomonas*

**Otu02244 -2.4 8.E-06 Ver/Verrucomicrobiae Chthoniobacterales *Chthoniobacteraceae Chthoniobacter***

**Otu01380 -2.7 6.E-08 Bact/Bacteroidia Chitinophagales *Chitinophagaceae* unknown**

Otu01846 -2.7 9.E-04 Pro/Gammaproteobacteria Betaproteobacteriales *Burkholderiaceae Burkholderia^1^*^)^

Otu01164 -3.2 6.E-04 Pro/Alphaproteobacteria Caulobacterales *Caulobacteraceae Caulobacter*

**Otu04746 -3.5 6.E-07 Bact/Bacteroidia Sphingobacteriales *Sphingobacteriaceae Mucilaginibacter***

Otu01714 -4.5 7.E-05 Pro/Gammaproteobacteria Betaproteobacteriales *Methylophilaceae Methylotenera*

**Otu01329 -5.6 6.E-07 Pro/Alphaproteobacteria Sphingomonadales *Sphingomonadaceae Novosphingobium***

Otu02296 -6.0 4.E-05 Pro/Alphaproteobacteria Azospirillales *Inquilinaceae Inquilinus*

Otu01113 -6.0 5.E-05 Ver/Verrucomicrobiae Verrucomicrobiales *Rubritaleaceae Luteolibacter*

**Otu01091 -6.2 1.E-09 Ver/Verrucomicrobiae Verrucomicrobiales *Verrucomicrobiaceae Verrucomicrobium***

Otu02448 -6.4 9.E-04 Bact/Bacteroidia Cytophagales *Spirosomaceae Dyadobacter*

Otu01109 -6.5 3.E-04 Bact/Bacteroidia Sphingobacteriales *Sphingobacteriaceae Mucilaginibacter*

**Otu02291 -6.5 6.E-12 Bact/Bacteroidia Sphingobacteriales *Sphingobacteriaceae Mucilaginibacter***

**Otu03565 -6.6 2.E-09 Bact/Bacteroidia Sphingobacteriales *Sphingobacteriaceae Mucilaginibacter***

Otu03668 -6.7 2.E-05 Pro/Alphaproteobacteria Sphingomonadales *Sphingomonadaceae Sphingomonas*

**Otu02037 -6.7 3.E-08 Bact/Bacteroidia Sphingobacteriales *Sphingobacteriaceae Mucilaginibacter***

Otu04501 -7.1 4.E-04 Bact/Bacteroidia Chitinophagales *Chitinophagaceae Taibaiella*

**Otu04241 -7.6 5.E-07 Ver/Verrucomicrobiae Verrucomicrobiales *Rubritaleaceae Luteolibacter***

Otu00860 -8.1 8.E-05 Pro/Alphaproteobacteria Sphingomonadales *Sphingomonadaceae Sphingomonas*

^________________________________________________________________________________________________________________________________________________________________________________________________________^

silt

C vs B **Otu02244 -2.2 2.E-06 Ver/Verrucomicrobiae Chthoniobacterales *Chthoniobacteraceae Chthoniobacter***

**Otu01113 -2.5 4.E-04 Ver/Verrucomicrobiae Verrucomicrobiales *Rubritaleaceae Luteolibacter***

**Otu01788 -2.9 2.E-04 Act/Acidimicrobiia Microtrichales *Ilumatobacteraceae* unknown**

**Otu01164 -3.3 2.E-04 Pro/Alphaproteobacteria Caulobacterales *Caulobacteraceae Caulobacter***

**Otu02903 -3.8 5.E-04 Bact/Bacteroidia Chitinophagales *Chitinophagaceae Pseudoflavitalea***

**Otu01091 -4.1 2.E-04 Ver/Verrucomicrobiae Verrucomicrobiales *Verrucomicrobiaceae Verrucomicrobium***

Otu00712 -6.4 2.E-04 Pat/Saccharimonadia Saccharimonadales unknown unknown

^________________________________________________________________________________________________________________________________________________________________________________________________________^

clay

C vs BH **Otu01002 -2.2 4.E-05 Bact/Bacteroidia Chitinophagales *Chitinophagaceae Ferruginibacter***

Otu02207 -2.5 4.E-04 Bact/Bacteroidia Chitinophagales *Chitinophagaceae* unknown

Otu01164 -2.5 5.E-05 Pro/Alphaproteobacteria Caulobacterales *Caulobacteraceae Caulobacter*

Otu01788 -2.7 1.E-04 Act/Acidimicrobiia Microtrichales *Ilumatobacteraceae* unknown

Otu00976 -3.4 3.E-04 Pro/Gammaproteobacteria Betaproteobacteriales *Nitrosomonadaceae* unknown

**Otu01380 -3.6 9.E-12 Bact/Bacteroidia Chitinophagales *Chitinophagaceae* unknown**

**Otu04746 -4.0 3.E-06 Bact/Bacteroidia Sphingobacteriales *Sphingobacteriaceae Mucilaginibacter***

Otu01714 -4.0 3.E-04 Pro/Gammaproteobacteria Betaproteobacteriales *Methylophilaceae Methylotenera*

Otu01753 -4.3 1.E-04 Arm/Fimbriimonadia Fimbriimonadales *Fimbriimonadaceae Fimbriimonas*

Otu01091 -5.2 3.E-04 Ver/Verrucomicrobiae Verrucomicrobiales *Verrucomicrobiaceae Verrucomicrobium*

**Otu02037 -6.1 1.E-07 Bact/Bacteroidia Sphingobacteriales *Sphingobacteriaceae Mucilaginibacter***

Otu03668 -6.1 7.E-04 Pro/Alphaproteobacteria Sphingomonadales *Sphingomonadaceae Sphingomonas*

Otu01341 -6.4 8.E-05 Pro/Gammaproteobacteria Salinisphaerales *Solimonadaceae Panacagrimonas*

**Otu01329 -6.5 7.E-09 Pro/Alphaproteobacteria Sphingomonadales *Sphingomonadaceae Novosphingobium***

**Otu02291 -6.6 1.E-06 Bact/Bacteroidia Sphingobacteriales *Sphingobacteriaceae Mucilaginibacter***

Otu01109 -6.7 9.E-04 Bact/Bacteroidia Sphingobacteriales *Sphingobacteriaceae Mucilaginibacter*

Otu01273 -6.9 2.E-04 Pro/Gammaproteobacteria Xanthomonadales *Rhodanobacteraceae Dokdonella*

**Otu03565 -7.3 6.E-10 Bact/Bacteroidia Sphingobacteriales *Sphingobacteriaceae Mucilaginibacter***

**Otu04241 -7.4 1.E-06 Ver/Verrucomicrobiae Verrucomicrobiales *Rubritaleaceae Luteolibacter***

Otu00860 -9.1 3.E-04 Pro/Alphaproteobacteria Sphingomonadales *Sphingomonadaceae Sphingomonas*

**Otu04854 -21.9 8.E-08 Bact/Bacteroidia Sphingobacteriales *Sphingobacteriaceae Mucilaginibacter***

**Otu04839 -23.0 1.E-08 Pro/Alphaproteobacteria Sphingomonadales *Sphingomonadaceae Sphingomonas***

^________________________________________________________________________________________________________________________________________________________________________________________________________^

silt

C vs BH Otu02640 3 4.E-04 Pro/Deltaproteobacteria Myxococcales *Nannocystaceae* unknown

**Otu03839 2 9.E-05 Act/Actinobacteria Micrococcales *Micrococcaceae Paenarthrobacter***

Otu00471 -2 7.E-04 Pat/Saccharimonadia Saccharimonadales *Saccharimonadaceae* unknown

**Otu01380 -2 2.E-05 Bact/Bacteroidia Chitinophagales *Chitinophagaceae* unknown**

**Otu01213 -2 3.E-20 Ver/Verrucomicrobiae Chthoniobacterales *Chthoniobacteraceae Chthoniobacter***

**Otu01804 -3 9.E-06 Pro/Alphaproteobacteria Caulobacterales *Caulobacteraceae* unknown**

**Otu02903 -3 2.E-05 Bact/Bacteroidia Chitinophagales *Chitinophagaceae Pseudoflavitalea***

**Otu01788 -3 1.E-08 Act/Acidimicrobiia Microtrichales *Ilumatobacteraceae* unknown**

**Otu01091 -4 1.E-05 Ver/Verrucomicrobiae Verrucomicrobiales *Verrucomicrobiaceae Verrucomicrobium***

Otu01269 -6 4.E-04 Ver/Verrucomicrobiae unknown unknown unknown

**Otu03168 -6 1.E-04 Pro/Alphaproteobacteria Sphingomonadales *Sphingomonadaceae Sphingomonas***

Otu03668 -6 3.E-04 Pro/Alphaproteobacteria Sphingomonadales Sphingomonadaceae Sphingomonas

**Otu03453 -6 2.E-04 Ver/Verrucomicrobiae Methylacidiphilales *Methylacidiphilaceae* unknown**

Otu01753 -6 2.E-04 Arm/Fimbriimonadia Fimbriimonadales *Fimbriimonadaceae Fimbriimonas*

Otu02670 -6 9.E-05 Act/Actinobacteria unknown unknown unknown

**Otu04951 -6 7.E-05 Pro/Alphaproteobacteria Sphingomonadales *Sphingomonadaceae Sphingomonas***

Otu00712 -7 9.E-06 Pat/Saccharimonadia Saccharimonadales unknown unknown

^________________________________________________________________________________________________________________________________________________________________________________________________________^

clay

C vs BA Otu02306 6.1 1.E-08 Pro/Deltaproteobacteria Myxococcales unknown unknown

Otu00992 5.8 3.E-04 Aci/unknown unknown unknown unknown

Otu05043 5.7 4.E-04 Ver/Verrucomicrobiae Verrucomicrobiales *Verrucomicrobiaceae* unknown

Otu04820 5.7 6.E-07 Ver/Verrucomicrobiae Pedosphaerales *Pedosphaeraceae* unknown

Otu01920 5.6 3.E-05 Pro/Deltaproteobacteria Myxococcales *Sandaracinaceae* unknown

Otu02259 3.7 2.E-06 Ver/Verrucomicrobiae Chthoniobacterales *Chthoniobacteraceae Chthoniobacter*

Otu02926 3.5 6.E-06 Pro/Deltaproteobacteria Myxococcales *Polyangiaceae Pajaroellobacter*

**Otu02264 3.1 1.E-11 Pro/Deltaproteobacteria Myxococcales *Phaselicystidaceae Phaselicystis***

Otu01760 3.0 3.E-04 Aci/unknown unknown unknown unknown

Otu00871 3.0 2.E-05 Pro/Gammaproteobacteria Betaproteobacteriales *Nitrosomonadaceae* unknown

Otu02319 2.6 1.E-08 Pro/Deltaproteobacteria Myxococcales unknown unknown

Otu02581 2.6 7.E-09 Pro/Deltaproteobacteria Myxococcales *Haliangiaceae Haliangium*

Otu01408 2.6 1.E-05 Ver/Verrucomicrobiae Chthoniobacterales *Chthoniobacteraceae Chthoniobacter*

Otu01141 2.3 1.E-03 Aci/unknown unknown unknown unknown

Otu01161 -2.1 2.E-04 Pro/Alphaproteobacteria Sphingomonadales *Sphingomonadaceae Altererythrobacter*

Otu00504 -2.3 8.E-08 Act/Actinobacteria Frankiales *Cryptosporangiaceae Fodinicola*

Otu01250 -2.3 8.E-04 Pro/Alphaproteobacteria Rhizobiales *Rhizobiaceae Mesorhizobium*

**Otu01884 -2.4 4.E-13 Pro/Gammaproteobacteria Betaproteobacteriales *Nitrosomonadaceae Nitrosospira***

Otu01360 -2.4 3.E-09 Bact/Bacteroidia Chitinophagales *Chitinophagaceae Flavisolibacter*

Otu00563 -2.6 4.E-05 Act/Actinobacteria Micrococcales *Microbacteriaceae Frigoribacterium*

Otu01421 -2.6 6.E-05 Pro/Deltaproteobacteria Myxococcales *Sandaracinaceae* unknown

**Otu02027 -2.6 1.E-15 Ver/Verrucomicrobiae Opitutales *Opitutaceae Opitutus***

Otu01380 -2.7 5.E-08 Bact/Bacteroidia Chitinophagales *Chitinophagaceae* unknown

Otu00615 -2.8 3.E-05 Act/Acidimicrobiia Microtrichales *Microtrichaceae* unknown

Otu00741 -2.8 3.E-04 Pro/Gammaproteobacteria Xanthomonadales *Xanthomonadaceae Lysobacter*

Otu03119 -2.8 1.E-03 Pla/Planctomycetacia Gemmatales *Gemmataceae Gemmata*

Otu05395 -2.9 5.E-05 Pla/Planctomycetacia Gemmatales *Gemmataceae Gemmata*

Otu00913 -3.0 2.E-04 Bact/Bacteroidia Sphingobacteriales unknown unknown

Otu02555 -3.0 3.E-04 Pro/Alphaproteobacteria Rhizobiales *Xanthobacteraceae Pseudolabrys*

Otu03981 -3.1 2.E-04 Pro/Alphaproteobacteria Sphingomonadales *Sphingomonadaceae Altererythrobacter*

Otu01728 -3.2 4.E-04 Bact/Bacteroidia Cytophagales *Microscillaceae Chryseolinea*

**Otu00765 -3.2 7.E-11 Pro/Alphaproteobacteria Rhizobiales *Devosiaceae Devosia***

Otu02436 -3.3 2.E-04 Pro/Alphaproteobacteria Reyranellales *Reyranellaceae Reyranella*

Otu01196 -3.4 5.E-08 Bact/Bacteroidia Cytophagales *Hymenobacteraceae Adhaeribacter*

Otu01392 -3.6 6.E-04 Pro/Gammaproteobacteria Xanthomonadales Rhodanobacteraceae Dokdonella

Otu01164 -3.6 3.E-05 Pro/Alphaproteobacteria Caulobacterales *Caulobacteraceae Caulobacter*

**Otu01165 -3.7 6.E-13 Pro/Gammaproteobacteria Xanthomonadales *Rhodanobacteraceae Dokdonella***

Otu01921 -3.7 1.E-07 Fir/Erysipelotrichia Erysipelotrichales *Erysipelotrichaceae Turicibacter*

Otu01404 -3.9 1.E-04 Gem/Gemmatimonadetes Gemmatimonadales *Gemmatimonadaceae Gemmatimonas*

**Otu01042 -3.9 2.E-09 Pro/Alphaproteobacteria Sphingomonadales *Sphingomonadaceae Sphingobium***

Otu01162 -4.0 1.E-07 Pro/Gammaproteobacteria Pseudomonadales *Pseudomonadaceae Pseudomonas*

Otu02059 -4.1 3.E-04 Act/Actinobacteria Frankiales *Nakamurellaceae Nakamurella*

Otu00806 -4.3 4.E-05 Pro/Alphaproteobacteria Rhizobiales *Rhizobiaceae Allorhizobium^2^*^)^

Otu02511 -4.4 4.E-05 Ver/Verrucomicrobiae Pedosphaerales *Pedosphaeraceae* unknown

Otu06656 -4.4 2.E-04 Fir/Clostridia Clostridiales *Peptostreptococcaceae* unknown

Otu01302 -4.5 4.E-09 Fib/Fibrobacteria Fibrobacterales *Fibrobacteraceae* unknown

Otu02640 -4.7 2.E-07 Pro/Deltaproteobacteria Myxococcales *Nannocystaceae* unknown

Otu01043 -4.7 2.E-07 Pro/Alphaproteobacteria Sphingomonadales *Sphingomonadaceae Novosphingobium*

Otu09086 -4.7 7.E-04 Fir/Clostridia Clostridiales *Peptostreptococcaceae Eubacterium_tenue*

Otu02075 -5.0 2.E-04 Act/Actinobacteria Corynebacteriales *Nocardiaceae Rhodococcus*

Otu03201 -5.1 3.E-04 Ver/Verrucomicrobiae Chthoniobacterales *Chthoniobacteraceae Chthoniobacter*

Otu05466 -5.2 1.E-03 Pro/Gammaproteobacteria Diplorickettsiales *Diplorickettsiaceae Aquicella*

Otu02098 -5.4 1.E-03 Fir/Clostridia Clostridiales *Ruminococcaceae Fastidiosipila*

Otu02496 -5.4 5.E-08 Ver/Verrucomicrobiae Pedosphaerales *Pedosphaeraceae* unknown

Otu08224 -5.5 8.E-04 Pro/Deltaproteobacteria Bdellovibrionales *Bacteriovoracaceae Peredibacter*

Otu05378 -5.6 5.E-04 Pro/Deltaproteobacteria Bdellovibrionales *Bdellovibrionaceae Bdellovibrio*

**Otu00907 -5.6 4.E-13 Fir/Clostridia Clostridiales *Peptostreptococcaceae Romboutsia***

Otu09050 -5.7 6.E-04 Pla/Phycisphaerae Tepidisphaerales unknown unknown

Otu01173 -5.7 2.E-09 Pro/Gammaproteobacteria Xanthomonadales *Rhodanobacteraceae Rhodanobacter*

Otu02449 -5.7 4.E-06 Bact/Bacteroidia Chitinophagales *Chitinophagaceae* unknown

Otu03618 -5.8 5.E-06 Pla/Planctomycetacia Pirellulales *Pirellulaceae Pirellula*

Otu02551 -5.9 4.E-04 Pat/unknown unknown unknown unknown

Otu08966 -6.0 8.E-04 Bact/Bacteroidia Sphingobacteriales unknown unknown

Otu05253 -6.0 5.E-04 Pat/unknown Cand_Uhrbacteria unknown unknown

Otu05297 -6.0 4.E-04 Ver/Verrucomicrobiae Chthoniobacterales *Chthoniobacteraceae* unknown

Otu00642 -6.0 6.E-04 Chl/Anaerolineae unknown unknown unknown

Otu01393 -6.1 1.E-03 Fir/Clostridia Clostridiales *Ruminococcaceae Fastidiosipila*

Otu01176 -6.1 4.E-07 Pro/Gammaproteobacteria Xanthomonadales *Rhodanobacteraceae Rhodanobacter*

Otu09993 -6.2 6.E-05 Fir/Erysipelotrichia Erysipelotrichales *Erysipelotrichaceae Solobacterium*

Otu00690 -6.2 7.E-05 Pro/Alphaproteobacteria Rhizobiales *Beijerinckiaceae Bosea*

Otu07744 -6.2 1.E-04 Pla/Planctomycetacia Planctomycetales unknown unknown

Otu01663 -6.2 1.E-04 Pat/Microgenomatia unknown unknown unknown

Otu05311 -6.3 3.E-05 Pro/Gammaproteobacteria Legionellales *Legionellaceae Legionella*

Otu00632 -6.3 6.E-04 Pat/Saccharimonadia Saccharimonadales unknown unknown

Otu00903 -6.3 6.E-07 Pro/Gammaproteobacteria Pseudomonadales *Pseudomonadaceae Pseudomonas*

Otu05778 -6.4 4.E-04 Pro/Gammaproteobacteria Betaproteobacteriales *Burkholderiaceae Advenella*

Otu02519 -6.4 5.E-05 Pla/Planctomycetacia Pirellulales *Pirellulaceae* unknown

Otu02215 -6.4 5.E-05 Ver/Verrucomicrobiae Chthoniobacterales *Chthoniobacteraceae* unknown

Otu02150 -6.5 3.E-05 Bact/Bacteroidia Bacteroidales unknown unknown

Otu01995 -6.6 2.E-04 Pro/Gammaproteobacteria Legionellales *Legionellaceae Legionella*

Otu04228 -6.6 2.E-04 Bact/Bacteroidia Bacteroidales *Dysgonomonadaceae Fermentimonas*

Otu03980 -6.7 8.E-05 Pro/Alphaproteobacteria Rhizobiales *Devosiaceae Devosia*

Otu04332 -6.7 6.E-04 Clo/Cloacimonadia Cloacimonadales unknown unknown

Otu01271 -7.1 6.E-06 Bact/Bacteroidia Bacteroidales unknown unknown

Otu01358 -7.1 4.E-04 Pla/Planctomycetacia Pirellulales *Pirellulaceae Pirellula*

Otu01301 -7.2 3.E-07 Pro/Alphaproteobacteria unknown unknown unknown

Otu04137 -7.2 6.E-04 Bact/Bacteroidia Sphingobacteriales *Sphingobacteriaceae Pedobacter*

Otu02079 -7.3 6.E-06 Bact/Bacteroidia Chitinophagales *Chitinophagaceae* unknown

**Otu00638 -7.4 1.E-17 Bact/Bacteroidia Chitinophagales *Chitinophagaceae* unknown**

Otu04009 -7.5 1.E-04 Ver/Verrucomicrobiae Chthoniobacterales *Chthoniobacteraceae Chthoniobacter*

Otu03901 -7.6 1.E-06 Pro/Alphaproteobacteria Caulobacterales *Caulobacteraceae Asticcacaulis*

Otu02456 -7.6 3.E-09 Pro/Gammaproteobacteria Xanthomonadales *Rhodanobacteraceae Rhodanobacter*

Otu00633 -8.0 1.E-08 Pat/Saccharimonadia Saccharimonadales unknown unknown

Otu00763 -8.0 4.E-07 Chl/Anaerolineae unknown unknown unknown

Otu02033 -8.0 4.E-06 Pro/Gammaproteobacteria Xanthomonadales *Xanthomonadaceae Pseudoxanthomonas*

Otu05193 -8.4 2.E-09 Ver/Verrucomicrobiae Verrucomicrobiales *Verrucomicrobiaceae Prosthecobacter*

Otu02598 -8.9 2.E-04 Pro/Alphaproteobacteria Sphingomonadales *Sphingomonadaceae Sphingopyxis*

Otu05340 -9.0 4.E-09 Pro/Alphaproteobacteria Micropepsales *Micropepsaceae* unknown

**Otu00894 -9.8 4.E-10 Pro/Alphaproteobacteria Caulobacterales *Caulobacteraceae Asticcacaulis***

**Otu00773 -9.9 2.E-12 Chl/Anaerolineae Anaerolineales *Anaerolineaceae* unknown**

^_______________________________________________________________________________________________________________________________________________________________________________________________________^

silt

C vs BA Otu00955 5.0 6.E-05 Pro/Deltaproteobacteria Myxococcales *Haliangiaceae Haliangium*

Otu06678 5.0 2.E-04 Aci/unknown unknown unknown unknown

**Otu00527 4.8 2.E-09 Act/Acidimicrobiia Microtrichales *Ilumatobacteraceae* unknown**

Otu06637 4.8 9.E-04 Bact/Bacteroidia Cytophagales *Microscillaceae* unknown

Otu00868 4.8 6.E-04 Ver/Verrucomicrobiae Verrucomicrobiales *Verrucomicrobiaceae Roseimicrobium*

Otu01709 4.8 6.E-04 Aci/unknown unknown unknown unknown

Otu00871 4.6 1.E-08 Pro/Gammaproteobacteria Betaproteobacteriales *Nitrosomonadaceae* unknown

Otu00992 4.5 2.E-06 Aci/unknown unknown unknown unknown

Otu01910 4.5 6.E-05 Ver/Verrucomicrobiae Verrucomicrobiales *Verrucomicrobiaceae* unknown

Otu04098 3.6 2.E-04 Chla/Chlamydiae Chlamydiales *Parachlamydiaceae* unknown

Otu00976 3.5 2.E-04 Pro/Gammaproteobacteria Betaproteobacteriales *Nitrosomonadaceae* unknown

**Otu01715 3.1 8.E-10 Pro/Gammaproteobacteria Betaproteobacteriales *Nitrosomonadaceae* unknown**

Otu03070 2.6 8.E-04 Ver/Verrucomicrobiae Chthoniobacterales *Chthoniobacteraceae Chthoniobacter*

Otu00710 2.5 1.E-05 Ver/Verrucomicrobiae Chthoniobacterales *Chthoniobacteraceae Chthoniobacter*

Otu02349 2.4 3.E-05 Aci/Thermoanaerobaculia Thermoanaerobaculales *Thermoanaerobaculaceae* unknown

Otu00715 2.2 1.E-07 Pla/Planctomycetacia Planctomycetales unknown unknown

Otu02720 2.1 9.E-04 Pla/Planctomycetacia Pirellulales *Pirellulaceae* unknown

Otu01289 -2.1 6.E-04 Pro/Deltaproteobacteria Myxococcales *Sandaracinaceae* unknown

Otu00899 -2.1 4.E-05 Pro/Deltaproteobacteria Myxococcales *Haliangiaceae Haliangium*

Otu01961 -2.2 2.E-08 Pla/unknown unknown unknown unknown

**Otu01884 -2.4 1.E-14 Pro/Gammaproteobacteria Betaproteobacteriales *Nitrosomonadaceae Nitrosospira***

Otu01392 -2.4 3.E-05 Pro/Gammaproteobacteria Xanthomonadales *Rhodanobacteraceae Dokdonella*

Otu04097 -2.4 2.E-04 Bact/Ignavibacteria unknown unknown unknown

Otu01258 -2.6 8.E-06 Pro/Gammaproteobacteria Xanthomonadales *Xanthomonadaceae Luteimonas*

Otu01234 -2.6 8.E-18 Pro/Gammaproteobacteria Betaproteobacteriales unknown unknown

Otu01826 -2.6 2.E-06 Pla/Planctomycetacia Pirellulales *Pirellulaceae* unknown

Otu06882 -2.7 3.E-04 Pro/Alphaproteobacteria Rhizobiales *Hyphomicrobiaceae Pedomicrobium*

Otu03888 -2.7 7.E-04 Pro/Alphaproteobacteria Reyranellales *Reyranellaceae* unknown

Otu02168 -2.8 7.E-05 Gem/Gemmatimonadetes Gemmatimonadales *Gemmatimonadaceae* unknown

**Otu00765 -2.9 2.E-11 Pro/Alphaproteobacteria Rhizobiales Devosiaceae Devosia**

Otu01161 -2.9 3.E-04 Pro/Alphaproteobacteria Sphingomonadales *Sphingomonadaceae Altererythrobacter*

Otu03986 -2.9 6.E-04 Ver/Verrucomicrobiae Pedosphaerales *Pedosphaeraceae* unknown

Otu00552 -3.0 6.E-04 Pat/Saccharimonadia Saccharimonadales unknown unknown

Otu02107 -3.0 2.E-04 Pla/Planctomycetacia Pirellulales *Pirellulaceae Pirellula*

Otu06098 -3.1 1.E-06 Aci/Holophagae unknown unknown unknown

Otu04070 -3.1 2.E-04 Pla/Planctomycetacia Isosphaerales *Isosphaeracea Aquisphaera*

Otu02141 -3.2 4.E-10 Pro/Gammaproteobacteria unknown unknown unknown

Otu03954 -3.2 6.E-08 Pla/Planctomycetacia Planctomycetales *Rubinisphaeraceae* unknown

**Otu01043 -3.2 2.E-17 Pro/Alphaproteobacteria Sphingomonadales Sphingomonadaceae Novosphingobium**

Otu04139 -3.3 1.E-04 Bact/Ignavibacteria unknown unknown unknown

Otu02362 -3.3 1.E-06 Bact/Bacteroidia Chitinophagales *Chitinophagaceae* unknown

Otu01983 -3.4 2.E-04 Dep/Babeliae Babeliales *Vermiphilaceae* unknown

Otu03911 -3.7 5.E-07 Pla/Planctomycetacia Planctomycetales *Rubinisphaeraceae* unknown

Otu03190 -3.7 9.E-04 Pla/Planctomycetacia Planctomycetales unknown unknown

Otu04376 -3.8 4.E-06 Bact/Ignavibacteria Kryptoniales unknown unknown

Otu02027 -3.8 9.E-09 Ver/Verrucomicrobiae Opitutales *Opitutaceae Opitutus*

Otu04380 -3.8 6.E-04 Ver/Verrucomicrobiae Opitutales *Opitutaceae Lacunisphaera*

Otu01358 -4.1 3.E-05 Pla/Planctomycetacia Pirellulales *Pirellulaceae Pirellula*

Otu02132 -4.1 6.E-06 Pla/Planctomycetacia Pirellulales *Pirellulaceae* unknown

Otu06430 -4.2 1.E-07 Pro/Deltaproteobacteria Myxococcales unknown unknown

Otu00797 -4.3 2.E-09 Pro/Alphaproteobacteria Rhizobiales *Devosiaceae Devosia*

Otu01284 -4.5 6.E-05 Pro/Alphaproteobacteria Rhizobiales *Xanthobacteraceae Pseudolabrys*

Otu02223 -4.5 1.E-05 Pla/Phycisphaerae Phycisphaerales *Phycisphaeraceae* unknown

Otu01042 -4.6 6.E-05 Pro/Alphaproteobacteria Sphingomonadales *Sphingomonadaceae Sphingobium*

Otu00759 -4.9 6.E-06 Bact/Bacteroidia Flavobacteriales *Flavobacteriaceae Flavobacterium*

Otu00705 -5.0 8.E-07 Pro/Gammaproteobacteria Xanthomonadales *Xanthomonadaceae Luteimonas*

Otu04072 -5.0 3.E-07 Chlo/unknown unknown unknown unknown

Otu03901 -5.1 2.E-04 Pro/Alphaproteobacteria Caulobacterales *Caulobacteraceae Asticcacaulis*

Otu01290 -5.1 3.E-04 Pro/Deltaproteobacteria Myxococcales *Sandaracinaceae* unknown

Otu04111 -5.1 3.E-05 Fib/Fibrobacteria Fibrobacterales *Fibrobacteraceae* unknown

Otu07445 -5.2 7.E-05 Pla/Planctomycetacia Pirellulales *Pirellulaceae Pirellula*

Otu01036 -5.2 8.E-07 Bact/Bacteroidia Chitinophagales *Chitinophagaceae* unknown

Otu04107 -5.3 5.E-06 Pro/Deltaproteobacteria Myxococcales unknown unknown

Otu07744 -5.5 6.E-04 Pla/Planctomycetacia Planctomycetales unknown unknown

Otu04110 -5.7 6.E-04 Pla/Planctomycetacia Planctomycetales unknown unknown

Otu04163 -5.8 5.E-04 Pla/Planctomycetacia Planctomycetales *Rubinisphaeraceae* unknown

Otu02079 -5.8 2.E-05 Bact/Bacteroidia Chitinophagales *Chitinophagaceae* unknown

Otu14080 -5.8 3.E-04 Ver/Verrucomicrobiae Chthoniobacterales *Terrimicrobiaceae* unknown

Otu02002 -5.9 9.E-04 Pro/Alphaproteobacteria Rhizobiales *Devosiaceae* *Devosia*

Otu03795 -5.9 2.E-04 Pat/Saccharimonadia Saccharimonadales unknown unknown

Otu02150 -6.0 1.E-03 Bact/Bacteroidia Bacteroidales unknown unknown

Otu07679 -6.0 2.E-04 Pro/Alphaproteobacteria unknown unknown unknown

Otu07317 -6.0 2.E-04 Pro/Gammaproteobacteria Pseudomonadales *Moraxellaceae* unknown

Otu04332 -6.2 6.E-04 Clo/Cloacimonadia Cloacimonadales unknown unknown

Otu04356 -6.2 4.E-04 Pro/Deltaproteobacteria Myxococcales *Sandaracinaceae* unknown

Otu03794 -6.3 3.E-04 Ver/Verrucomicrobiae Opitutales *Opitutaceae* *Opitutus*

Otu07436 -6.4 1.E-04 Ver/Verrucomicrobiae Pedosphaerales *Pedosphaeraceae* unknown

Otu04159 -6.5 1.E-05 Pla/Planctomycetacia Planctomycetales unknown unknown

Otu01271 -6.5 5.E-04 Bact/Bacteroidia Bacteroidales unknown unknown

**Otu00638 -6.5 2.E-11 Bact/Bacteroidia Chitinophagales Chitinophagaceae unknown**

Otu05193 -6.6 2.E-04 Ver/Verrucomicrobiae Verrucomicrobiales *Verrucomicrobiaceae Prosthecobacter*

Otu04231 -6.6 1.E-05 Pro/Deltaproteobacteria Myxococcales *Sandaracinaceae* unknown

Otu04067 -6.6 1.E-04 Pla/Planctomycetacia Pirellulales *Pirellulaceae Pirellula*

Otu01987 -6.6 2.E-05 Pro/Alphaproteobacteria Micavibrionales unknown unknown

Otu04022 -6.6 4.E-05 Pat/Berkelbacteria unknown unknown unknown

Otu04582 -6.7 3.E-05 Pro/Gammaproteobacteria Pseudomonadales *Moraxellaceae* unknown

Otu03914 -6.7 1.E-05 Bact/Bacteroidia Chitinophagales *Chitinophagaceae Ferruginibacter*

Otu07871 -6.8 7.E-06 Pro/Gammaproteobacteria Betaproteobacteriales *Burkholderiaceae Limnobacter*

Otu04576 -6.8 2.E-04 Pro/Gammaproteobacteria Xanthomonadales *Xanthomonadaceae Pseudoxanthomonas*

Otu00761 -6.9 5.E-04 Pro/Gammaproteobacteria Betaproteobacteriales *Burkholderiaceae Herminiimonas*

Otu03950 -7.0 4.E-06 Pro/Alphaproteobacteria Micavibrionales unknown unknown

Otu03827 -7.1 3.E-05 Pro/Gammaproteobacteria Betaproteobacteriales *Burkholderiaceae Candidimonas*

Otu01997 -7.1 1.E-06 Pro/Deltaproteobacteria Myxococcales *Sandaracinaceae* unknown

Otu04217 -7.3 1.E-06 Bact/Bacteroidia Chitinophagales *Chitinophagaceae* unknown

Otu00763 -7.4 3.E-06 Chlo/Anaerolineae unknown unknown unknown

Otu04228 -7.4 4.E-06 Bact/Bacteroidia Bacteroidales *Dysgonomonadaceae Fermentimonas*

Otu00894 -7.5 3.E-06 Pro/Alphaproteobacteria Caulobacterales *Caulobacteraceae Asticcacaulis*

Otu00594 -7.6 3.E-06 Pat/Parcubacteria Cand_Zambryskibacteria unknown unknown

Otu01404 -7.6 6.E-07 Gem/Gemmatimonadetes Gemmatimonadales *Gemmatimonadaceae Gemmatimonas*

Otu02080 -7.6 5.E-07 Bact/Bacteroidia Flavobacteriales unknown unknown

Otu03976 -7.7 3.E-08 Pro/Alphaproteobacteria Rhizobiales *Beijerinckiaceae* unknown

Otu04220 -7.7 9.E-07 Fib/Fibrobacteria Fibrobacterales *Fibrobacteraceae* unknown

Otu03849 -8.0 3.E-06 Bact/Bacteroidia Chitinophagales *Chitinophagaceae Ferruginibacter*

Otu04557 -8.9 1.E-08 Pro/Deltaproteobacteria Myxococcales unknown unknown

Otu01288 -9.0 9.E-09 Pat/unknown unknown unknown unknown

**Otu00786 -9.1 3.E-10 Bact/Bacteroidia Cytophagales *Microscillaceae* unknown**

Otu01273 -9.1 2.E-08 Pro/Gammaproteobacteria Xanthomonadales *Rhodanobacteraceae Dokdonella*

Otu01176 -9.3 8.E-06 Pro/Gammaproteobacteria Xanthomonadales *Rhodanobacteraceae Rhodanobacter*

**Otu02033 -9.7 1.E-09 Pro/Gammaproteobacteria Xanthomonadales *Xanthomonadaceae Pseudoxanthomonas***

Otu02219 -9.8 1.E-08 Pla/Planctomycetacia Planctomycetales *Schlesneriaceae Planctopirus*

**Otu00773 -9.9 1.E-12 Chlo/Anaerolineae Anaerolineales *Anaerolineaceae* unknown**

Otu02034 -10.4 5.E-04 Pro/Gammaproteobacteria Xanthomonadales *Xanthomonadaceae Thermomonas*

Otu01026 -10.5 3.E-05 Bact/Bacteroidia Flavobacteriales *Weeksellaceae Chryseobacterium*

**Otu01305 -23.9 9.E-10 Bact/Bacteroidia Sphingobacteriales *Sphingobacteriaceae Solitalea***

^________________________________________________________________________________________________________________________________________________________________________________________________________^

clay

C vs BHA Otu04820 6.8 2.E-10 Ver/Verrucomicrobiae Pedosphaerales *Pedosphaeraceae* unknown

Otu02306 6.7 7.E-11 Pro/Deltaproteobacteria Myxococcales unknown unknown

Otu00992 6.0 2.E-05 Aci/unknown unknown unknown unknown

Otu05043 6.0 4.E-05 Ver/Verrucomicrobiae Verrucomicrobiales *Verrucomicrobiaceae* unknown

Otu03370 5.8 1.E-04 Aci/Acidobacteriia Solibacterales *Solibacteraceae Bryobacter*

Otu00871 5.6 2.E-07 Pro/Gammaproteobacteria Betaproteobacteriales *Nitrosomonadaceae* unknown

Otu08435 5.3 2.E-04 Aci/Acidobacteriia Solibacterales *Solibacteraceae Candidatus_Solibacter*

Otu03301 5.3 4.E-06 Ver/Verrucomicrobiae Pedosphaerales *Pedosphaeraceae* unknown

Otu05076 5.3 2.E-05 Ver/Verrucomicrobiae Pedosphaerales *Pedosphaeraceae* unknown

Otu02259 4.9 4.E-08 Ver/Verrucomicrobiae Chthoniobacterales *Chthoniobacteraceae Chthoniobacter*

Otu01347 4.8 4.E-04 Pla/unknown unknown unknown unknown

Otu05543 4.6 8.E-04 Pro/Deltaproteobacteria Myxococcales unknown unknown

Otu03963 4.5 3.E-04 Pla/Planctomycetacia Gemmatales *Gemmataceae* unknown

Otu03985 4.2 1.E-04 Pro/Gammaproteobacteria Betaproteobacteriales *Nitrosomonadaceae* unknown

Otu02319 4.2 1.E-11 Pro/Deltaproteobacteria Myxococcales unknown unknown

Otu01743 4.1 7.E-09 Pla/Planctomycetacia Pirellulales *Pirellulaceae Pirellula*

Otu01141 3.7 5.E-08 Aci/unknown unknown unknown unknown

Otu08175 3.4 4.E-04 Pla/unknown unknown unknown unknown

Otu04330 3.3 4.E-04 Pla/Planctomycetacia Gemmatales *Gemmataceae* unknown

**Otu02581 3.2 2.E-12 Pro/Deltaproteobacteria Myxococcales Haliangiaceae Haliangium**

Otu03331 3.1 4.E-04 Aci/unknown unknown unknown unknown

Otu05566 3.1 6.E-04 Lat/unknown unknown unknown unknown

Otu02465 3.1 3.E-04 Pla/Planctomycetacia Gemmatales *Gemmataceae* unknown

Otu01408 3.0 6.E-07 Ver/Verrucomicrobiae Chthoniobacterales *Chthoniobacteraceae Chthoniobacter*

Otu01126 2.9 4.E-04 Aci/unknown unknown unknown *Luteitalea*

Otu02231 2.8 6.E-04 Lat/unknown unknown unknown unknown

Otu01122 2.7 4.E-12 Aci/unknown unknown unknown unknown

Otu02954 2.7 7.E-05 Aci/unknown unknown unknown unknown

Otu02470 2.6 7.E-04 Pla/unknown unknown unknown unknown

Otu00702 2.6 3.E-05 Aci/unknown unknown unknown unknown

Otu01715 2.5 9.E-04 Pro/Gammaproteobacteria Betaproteobacteriales *Nitrosomonadaceae* unknown

Otu00671 2.5 7.E-07 Aci/Acidobacteriia Solibacterales *Solibacteraceae Candidatus_Solibacter*

Otu01213 2.4 7.E-04 Ver/Verrucomicrobiae Chthoniobacterales *Chthoniobacteraceae Chthoniobacter*

Otu00889 2.3 3.E-07 Aci/unknown unknown unknown unknown

Otu01218 2.3 6.E-04 Aci/unknown unknown unknown unknown

Otu00715 2.2 2.E-10 Pla/Planctomycetacia Planctomycetales unknown unknown

Otu02264 2.1 6.E-08 Pro/Deltaproteobacteria Myxococcales *Phaselicystidaceae Phaselicystis*

Otu01360 -2.1 1.E-07 Bact/Bacteroidia Chitinophagales *Chitinophagaceae Flavisolibacter*

Otu01354 -2.1 3.E-07 Pro/Gammaproteobacteria Xanthomonadales *Xanthomonadaceae Lysobacter*

Otu01250 -2.1 4.E-08 Pro/Alphaproteobacteria Rhizobiales *Rhizobiaceae Mesorhizobium*

**Otu00504 -2.2 7.E-15 Act/Actinobacteria Frankiales Cryptosporangiaceae Fodinicola**

**Otu00899 -2.2 1.E-12 Pro/Deltaproteobacteria Myxococcales Haliangiaceae Haliangium**

Otu05145 -2.3 6.E-05 Pro/Alphaproteobacteria Caulobacterales *Caulobacteraceae Phenylobacterium*

Otu00485 -2.3 3.E-04 Pat/Saccharimonadia Saccharimonadales unknown unknown

Otu00741 -2.3 5.E-06 Pro/Gammaproteobacteria Xanthomonadales *Xanthomonadaceae Lysobacter*

Otu02503 -2.3 8.E-04 Ver/Verrucomicrobiae Opitutales *Opitutaceae Lacunisphaera*

Otu00615 -2.4 2.E-04 Act/Acidimicrobiia Microtrichales *Microtrichaceae* unknown

Otu02051 -2.4 5.E-05 Arm/unknown unknown unknown unknown

Otu01164 -2.4 3.E-06 Pro/Alphaproteobacteria Caulobacterales *Caulobacteraceae Caulobacter*

Otu01174 -2.5 2.E-10 Bact/Bacteroidia Chitinophagales *Chitinophagaceae* unknown

Otu01196 -2.5 2.E-07 Bact/Bacteroidia Cytophagales *Hymenobacteraceae Adhaeribacter*

Otu01161 -2.5 5.E-08 Pro/Alphaproteobacteria Sphingomonadales *Sphingomonadaceae Altererythrobacter*

Otu01421 -2.6 4.E-08 Pro/Deltaproteobacteria Myxococcales *Sandaracinaceae* unknown

Otu00806 -2.7 2.E-05 Pro/Alphaproteobacteria Rhizobiales *Rhizobiaceae Allorhizobium^2^*^)^

Otu03134 -2.7 2.E-04 Pro/Gammaproteobacteria Betaproteobacteriales *Methylophilaceae* unknown

Otu01162 -2.7 3.E-06 Pro/Gammaproteobacteria Pseudomonadales *Pseudomonadaceae* Pseudomonas

Otu01917 -2.9 3.E-04 Act/Acidimicrobiia Microtrichales *Microtrichaceae* unknown

Otu03981 -2.9 9.E-04 Pro/Alphaproteobacteria Sphingomonadales *Sphingomonadaceae Altererythrobacter*

**Otu01884 -2.9 5.E-33 Pro/Gammaproteobacteria Betaproteobacteriales *Nitrosomonadaceae Nitrosospira***

**Otu00765 -3.1 2.E-20 Pro/Alphaproteobacteria Rhizobiales *Devosiaceae Devosia***

Otu00563 -3.1 3.E-06 Act/Actinobacteria Micrococcales *Microbacteriaceae Frigoribacterium*

Otu05381 -3.2 1.E-04 Pro/Alphaproteobacteria Sphingomonadales *Sphingomonadaceae Sphingomonas*

Otu01380 -3.2 2.E-11 Bact/Bacteroidia Chitinophagales *Chitinophagaceae* unknown

Otu04337 -3.3 6.E-05 Pro/Alphaproteobacteria Caulobacterales *Caulobacteraceae Phenylobacterium*

Otu01165 -3.3 1.E-11 Pro/Gammaproteobacteria Xanthomonadales *Rhodanobacteraceae Dokdonella*

Otu08687 -3.4 4.E-04 Chlo/Chloroflexia Thermomicrobiales unknown unknown

Otu01042 -3.5 7.E-06 Pro/Alphaproteobacteria Sphingomonadales *Sphingomonadaceae Sphingobium*

Otu02555 -3.5 2.E-10 Pro/Alphaproteobacteria Rhizobiales *Xanthobacteraceae Pseudolabrys*

Otu09020 -3.7 2.E-04 Pla/Planctomycetacia Gemmatales *Gemmataceae Gemmata*

Otu02059 -4.0 6.E-05 Act/Actinobacteria Frankiales *Nakamurellaceae Nakamurella*

Otu01404 -4.1 3.E-07 Gem/Gemmatimonadetes Gemmatimonadales *Gemmatimonadaceae Gemmatimonas*

Otu01392 -4.1 2.E-05 Pro/Gammaproteobacteria Xanthomonadales *Rhodanobacteraceae Dokdonella*

Otu01921 -4.1 8.E-11 Fir/Erysipelotrichia Erysipelotrichales *Erysipelotrichaceae Turicibacter*

Otu01291 -4.1 8.E-04 Bact/Bacteroidia Chitinophagales *Chitinophagaceae* unknown

Otu04292 -4.4 2.E-06 Bact/Bacteroidia Chitinophagales *Chitinophagaceae Flavitalea*

Otu02640 -4.4 5.E-06 Pro/Deltaproteobacteria Myxococcales *Nannocystaceae* unknown

Otu01043 -4.6 5.E-08 Pro/Alphaproteobacteria Sphingomonadales *Sphingomonadaceae Novosphingobium*

Otu06656 -4.6 1.E-05 Fir/Clostridia Clostridiales *Peptostreptococcaceae* unknown

Otu03618 -4.9 3.E-04 Pla/Planctomycetacia Pirellulales *Pirellulaceae Pirellula*

Otu01302 -4.9 2.E-08 Fib/Fibrobacteria Fibrobacterales *Fibrobacteraceae* unknown

Otu09086 -5.3 2.E-05 Fir/Clostridia Clostridiales *Peptostreptococcaceae Eubacterium_tenue* Otu04229 -5.3 7.E-04 Act/Acidimicrobiia Microtrichales *Microtrichaceae* unknown

Otu09050 -5.3 4.E-04 Pla/Phycisphaerae Tepidisphaerales unknown unknown

Otu10266 -5.5 6.E-04 Fir/Clostridia Clostridiales unknown *Sedimentibacter*

Otu10516 -5.5 5.E-04 Pro/Deltaproteobacteria Myxococcales unknown unknown

Otu08224 -5.5 5.E-04 Pro/Deltaproteobacteria Bdellovibrionales *Bacteriovoracaceae Peredibacter*

**Otu01173 -5.6 8.E-18 Pro/Gammaproteobacteria Xanthomonadales *Rhodanobacteraceae Rhodanobacter***

Otu03980 -5.6 1.E-04 Pro/Alphaproteobacteria Rhizobiales *Devosiaceae Devosia*

Otu03965 -5.6 7.E-04 Bact/Bacteroidia Chitinophagales *Chitinophagaceae Chitinophaga*

Otu05576 -5.6 7.E-04 Fir/Bacilli Lactobacillales *Carnobacteriaceae Trichococcus*

Otu02496 -5.7 7.E-11 Ver/Verrucomicrobiae Pedosphaerales *Pedosphaeraceae* unknown

Otu00497 -5.7 3.E-04 Pat/Saccharimonadia Saccharimonadales unknown unknown

Otu01176 -5.7 1.E-07 Pro/Gammaproteobacteria Xanthomonadales *Rhodanobacteraceae Rhodanobacter*

Otu00632 -5.7 3.E-04 Pat/Saccharimonadia Saccharimonadales unknown unknown

Otu05355 -5.7 4.E-04 Pro/Gammaproteobacteria Xanthomonadales *Rhodanobacteraceae Rhodanobacter*

Otu04284 -5.7 5.E-04 Pro/Alphaproteobacteria Sphingomonadales *Sphingomonadaceae* unknown

Otu05466 -5.7 4.E-04 Pro/Gammaproteobacteria Diplorickettsiales *Diplorickettsiaceae Aquicella*

Otu05331 -5.7 8.E-04 Chla(Chlamydiae Chlamydiales *Parachlamydiaceae Neochlamydia*

**Otu00907 -5.9 3.E-18 Fir/Clostridia Clostridiales *Peptostreptococcaceae Romboutsia***

Otu05297 -5.9 1.E-04 Ver/Verrucomicrobiae Chthoniobacterales *Chthoniobacteraceae* unknown

Otu02449 -5.9 2.E-09 Bact/Bacteroidia Chitinophagales *Chitinophagaceae* unknown

Otu02509 -5.9 3.E-04 Fir/Clostridia Clostridiales *Christensenellaceae* *Christensenellaceae*_R7

Otu04582 -6.0 9.E-05 Pro/Gammaproteobacteria Pseudomonadales *Moraxellaceae* unknown

Otu05316 -6.0 6.E-04 Bact/Bacteroidia Chitinophagales unknown unknown

Otu05301 -6.0 1.E-04 Pro/Gammaproteobacteria unknown unknown unknown

Otu02012 -6.0 2.E-04 Cya/Melainabacteria Vampirovibrionales unknown unknown

Otu02033 -6.0 2.E-06 Pro/Gammaproteobacteria Xanthomonadales *Xanthomonadaceae Pseudoxanthomonas*

Otu07666 -6.2 4.E-05 Pro/Gammaproteobacteria Betaproteobacteriales *Burkholderiaceae Advenella*

Otu05378 -6.2 4.E-05 Pro/Deltaproteobacteria Bdellovibrionales *Bdellovibrionaceae Bdellovibrio*

Otu00642 -6.2 9.E-05 Chlo/Anaerolineae unknown unknown unknown

Otu01995 -6.3 6.E-05 Pro/Gammaproteobacteria Legionellales *Legionellaceae Legionella*

Otu05311 -6.3 1.E-05 Pro/Gammaproteobacteria Legionellales *Legionellaceae Legionella*

Otu01358 -6.4 7.E-05 Pla/Planctomycetacia Pirellulales *Pirellulaceae Pirellula*

Otu03867 -6.4 4.E-05 Fir/Clostridia Clostridiales *Christensenellaceae Christensenellaceae*_R7

Otu02521 -6.4 1.E-05 Pla/Phycisphaerae Tepidisphaerales unknown unknown

Otu05778 -6.5 2.E-05 Pro/Gammaproteobacteria Betaproteobacteriales *Burkholderiaceae Advenella*

Otu09041 -6.5 2.E-04 Pro/Alphaproteobacteria Caulobacterales *Caulobacteraceae Brevundimonas*

Otu01663 -6.5 5.E-05 Pat/Microgenomatia unknown unknown unknown

Otu01393 -6.5 8.E-06 Fir/Clostridia Clostridiales *Ruminococcaceae Fastidiosipila*

Otu02150 -6.6 1.E-05 Bact/Bacteroidia Bacteroidales unknown unknown

Otu02215 -6.6 2.E-05 Ver/Verrucomicrobiae Chthoniobacterales *Chthoniobacteraceae* unknown

Otu03901 -6.7 2.E-05 Pro/Alphaproteobacteria Caulobacterales *Caulobacteraceae Asticcacaulis*

Otu09993 -6.7 3.E-06 Fir/Erysipelotrichia Erysipelotrichales *Erysipelotrichaceae Solobacterium*

Otu01301 -6.9 1.E-06 Pro/Alphaproteobacteria unknown unknown unknown

Otu04228 -7.1 3.E-06 Bact/Bacteroidia Bacteroidales *Dysgonomonadaceae Fermentimonas*

Otu05217 -7.1 2.E-05 Bact/Bacteroidia Bacteroidales unknown unknown

Otu02079 -7.1 4.E-07 Bact/Bacteroidia Chitinophagales *Chitinophagaceae* unknown

Otu02519 -7.1 2.E-06 Pla/Planctomycetacia Pirellulales *Pirellulaceae* unknown

**Otu00638 -7.3 2.E-35 Bact/Bacteroidia Chitinophagales *Chitinophagaceae* unknown**

Otu04332 -7.3 3.E-06 Clo/Cloacimonadia Cloacimonadales unknown unknown

Otu05193 -7.4 3.E-07 Ver/Verrucomicrobiae Verrucomicrobiales *Verrucomicrobiaceae Prosthecobacter*

Otu01271 -7.6 3.E-07 Bact/Bacteroidia Bacteroidales unknown unknown

Otu00633 -7.7 3.E-08 Pat/Saccharimonadia Saccharimonadales unknown unknown

Otu00763 -7.7 4.E-08 Chlo/Anaerolineae unknown unknown unknown

Otu02456 -8.0 1.E-11 Pro/Gammaproteobacteria Xanthomonadales *Rhodanobacteraceae Rhodanobacter*

Otu02598 -8.5 4.E-08 Pro/Alphaproteobacteria Sphingomonadales *Sphingomonadaceae Sphingopyxis*

Otu00894 -9.2 1.E-09 Pro/Alphaproteobacteria Caulobacterales *Caulobacteraceae Asticcacaulis*

**Otu00773 -9.7 2.E-13 Chlo/Anaerolineae Anaerolineales *Anaerolineaceae* unknown**

**Otu05340 -10.1 6.E-13 Pro/Alphaproteobacteria Micropepsales *Micropepsaceae* unknown**

^________________________________________________________________________________________________________________________________________________________________________________________________________^

silt

C vs BHA **Otu00527 6.9 3.E-11 Act/Acidimicrobiia Microtrichales *Ilumatobacteraceae* unknown**

Otu00992 5.8 1.E-07 Aci/unknown unknown unknown unknown

Otu01709 5.4 1.E-04 Aci/unknown unknown unknown unknown

Otu06594 5.1 9.E-04 Pla/Planctomycetacia Pirellulales *Pirellulaceae* unknown

Otu06637 5.0 5.E-04 Bact/Bacteroidia Cytophagales *Microscillaceae* unknown

Otu00868 4.9 4.E-04 Ver/Verrucomicrobiae Verrucomicrobiales *Verrucomicrobiaceae Roseimicrobium*

Otu02357 4.9 8.E-04 Aci/Acidobacteriia Solibacterales *Solibacteraceae Paludibaculum*

**Otu00871 4.6 6.E-11 Pro/Gammaproteobacteria Betaproteobacteriales *Nitrosomonadaceae* unknown**

Otu00976 4.2 2.E-04 Pro/Gammaproteobacteria Betaproteobacteriales *Nitrosomonadaceae* unknown

Otu02301 4.0 1.E-04 Ver/Verrucomicrobiae Pedosphaerales *Pedosphaeraceae* unknown

Otu00955 4.0 2.E-04 Pro/Deltaproteobacteria Myxococcales *Haliangiaceae Haliangium*

Otu01910 3.6 1.E-05 Ver/Verrucomicrobiae Verrucomicrobiales *Verrucomicrobiaceae* unknown

Otu01715 3.3 1.E-10 Pro/Gammaproteobacteria Betaproteobacteriales *Nitrosomonadaceae* unknown

Otu06586 3.3 1.E-04 Pla/Planctomycetacia Gemmatales *Gemmataceae* unknown

Otu01763 3.1 6.E-06 Bact/Bacteroidia Cytophagales *Microscillaceae* unknown

Otu02909 3.1 7.E-04 Pro/Gammaproteobacteria Xanthomonadales *Rhodanobacteraceae Tahibacter*

Otu00967 2.6 2.E-04 Pro/Gammaproteobacteria Betaproteobacteriales *Nitrosomonadaceae* unknown

Otu01858 2.6 4.E-04 Act/Thermoleophilia Solirubrobacterales *Solirubrobacteraceae* unknown

Otu03191 2.4 9.E-04 Pro/Deltaproteobacteria Myxococcales *Sandaracinaceae* unknown

Otu02650 2.3 7.E-05 Pro/Alphaproteobacteria Micropepsales *Micropepsaceae* unknown

Otu00715 2.2 7.E-11 Pla/Planctomycetacia Planctomycetales unknown unknown

Otu03447 2.2 3.E-08 Nit/Nitrospira Nitrospirales *Nitrospiraceae Nitrospira*

Otu01164 -2.1 3.E-04 Pro/Alphaproteobacteria Caulobacterales *Caulobacteraceae Caulobacter*

Otu00899 -2.1 1.E-04 Pro/Deltaproteobacteria Myxococcales *Haliangiaceae Haliangium*

Otu02001 -2.3 5.E-04 Pla/Planctomycetacia Pirellulales *Pirellulaceae Pirellula*

Otu01034 -2.5 8.E-04 Pro/Alphaproteobacteria Rhodobacterales *Rhodobacteraceae Pseudorhodobacter*

**Otu01884 -2.6 1.E-16 Pro/Gammaproteobacteria Betaproteobacteriales *Nitrosomonadaceae Nitrosospira***

Otu01258 -2.7 3.E-07 Pro/Gammaproteobacteria Xanthomonadales *Xanthomonadaceae Luteimonas*

Otu01826 -2.7 2.E-07 Pla/Planctomycetacia Pirellulales *Pirellulaceae* unknown

Otu04097 -2.7 1.E-05 Bact/Ignavibacteria unknown unknown unknown

Otu04183 -2.7 8.E-04 Pla/Planctomycetacia Pirellulales *Pirellulaceae Pirellula*

Otu06882 -2.7 6.E-04 Pro/Alphaproteobacteria Rhizobiales *Hyphomicrobiaceae Pedomicrobium*

Otu01234 -2.8 1.E-16 Pro/Gammaproteobacteria Betaproteobacteriales unknown unknown

**Otu01392 -2.8 6.E-11 Pro/Gammaproteobacteria Xanthomonadales *Rhodanobacteraceae Dokdonella***

Otu07373 -2.8 7.E-04 Pla/Planctomycetacia Planctomycetales *Rubinisphaeraceae* unknown

Otu02154 -2.8 7.E-05 Pla/Planctomycetacia Pirellulales *Pirellulaceae Pirellula*

Otu02141 -2.9 2.E-07 Pro/Gammaproteobacteria unknown unknown unknown

Otu00485 -2.9 9.E-04 Pat/Saccharimonadia Saccharimonadales unknown unknown

**Otu00765 -3.0 1.E-16 Pro/Alphaproteobacteria Rhizobiales *Devosiaceae Devosia***

Otu01161 -3.0 5.E-04 Pro/Alphaproteobacteria Sphingomonadales *Sphingomonadaceae Altererythrobacter*

Otu02168 -3.0 1.E-06 Gem/Gemmatimonadetes Gemmatimonadales *Gemmatimonadaceae* unknown

Otu00794 -3.1 2.E-04 Bact/Bacteroidia Chitinophagales *Saprospiraceae* unknown

Otu02027 -3.1 5.E-05 Ver/Verrucomicrobiae Opitutales *Opitutaceae Opitutus*

Otu02004 -3.2 9.E-06 Pla/Planctomycetacia Pirellulales *Pirellulaceae* unknown

**Otu01043 -3.2 1.E-24 Pro/Alphaproteobacteria Sphingomonadales *Sphingomonadaceae Novosphingobium***

Otu00552 -3.3 5.E-06 Pat/Saccharimonadia Saccharimonadales unknown unknown

Otu06098 -3.4 2.E-07 Aci/Holophagae unknown unknown unknown

Otu03954 -3.4 7.E-09 Pla/Planctomycetacia Planctomycetales *Rubinisphaeraceae* unknown

Otu04070 -3.4 4.E-06 Pla/Planctomycetacia Isosphaerales *Isosphaeraceae Aquisphaera*

Otu01394 -3.5 8.E-05 Pro/Alphaproteobacteria Rhizobiales *Rhizobiaceae Mesorhizobium*

Otu02223 -3.5 1.E-04 Pla/Phycisphaerae Phycisphaerales *Phycisphaeraceae* unknown

Otu02362 -3.6 3.E-08 Bact/Bacteroidia Chitinophagales *Chitinophagaceae* unknown

Otu01983 -3.7 4.E-05 Dep/Babeliae Babeliales *Vermiphilaceae* unknown

Otu04376 -3.7 7.E-06 Bact/Ignavibacteria Kryptoniales unknown unknown

Otu06430 -3.9 8.E-05 Pro/Deltaproteobacteria Myxococcales unknown unknown

Otu04081 -4.1 3.E-04 Pro/Deltaproteobacteria Bdellovibrionales *Bdellovibrionaceae* unknown

Otu03911 -4.1 3.E-08 Pla/Planctomycetacia Planctomycetales *Rubinisphaeraceae* unknown

Otu02132 -4.1 3.E-04 Pla/Planctomycetacia Pirellulales *Pirellulaceae* unknown

**Otu00797 -4.2 4.E-14 Pro/Alphaproteobacteria Rhizobiales *Devosiaceae Devosia***

Otu02021 -4.2 5.E-04 Pro/Deltaproteobacteria Myxococcales *Nannocystaceae Nannocystis*

Otu01284 -4.3 2.E-06 Pro/Alphaproteobacteria Rhizobiales *Xanthobacteraceae Pseudolabrys*

Otu04072 -4.5 7.E-06 Chlo/unknown unknown unknown unknown

Otu00759 -4.5 2.E-07 Bact/Bacteroidia Flavobacteriales *Flavobacteriaceae Flavobacterium*

Otu01290 -4.7 4.E-04 Pro/Deltaproteobacteria Myxococcales *Sandaracinaceae* unknown

Otu01358 -4.7 3.E-08 Pla/Planctomycetacia Pirellulales *Pirellulaceae Pirellula*

**Otu01036 -4.8 4.E-12 Bact/Bacteroidia Chitinophagales *Chitinophagaceae* unknown**

Otu02598 -4.9 2.E-04 Pro/Alphaproteobacteria Sphingomonadales *Sphingomonadaceae Sphingopyxis*

Otu00705 -5.2 9.E-09 Pro/Gammaproteobacteria Xanthomonadales *Xanthomonadaceae Luteimonas*

Otu00633 -5.5 9.E-04 Pat/Saccharimonadia Saccharimonadales unknown unknown

Otu07445 -5.5 2.E-05 Pla/Planctomycetacia Pirellulales *Pirellulaceae Pirellula*

Otu03943 -5.6 1.E-03 Pro/Gammaproteobacteria Xanthomonadales *Xanthomonadaceae Pseudoxanthomonas*

Otu08011 -5.6 9.E-04 Bact/Bacteroidia Cytophagales *Cytophagaceae Sporocytophaga*

Otu03794 -5.8 4.E-04 Ver/Verrucomicrobiae Opitutales *Opitutaceae Opitutus*

Otu04228 -5.9 5.E-04 Bact/Bacteroidia Bacteroidales *Dysgonomonadaceae Fermentimonas*

Otu02079 -5.9 5.E-06 Bact/Bacteroidia Chitinophagales *Chitinophagaceae* unknown

Otu02150 -5.9 1.E-04 Bact/Bacteroidia Bacteroidales unknown unknown

Otu06784 -6.0 4.E-04 Chla/Chlamydiae Chlamydiales *Parachlamydiaceae* unknown

Otu04095 -6.0 1.E-04 Pro/Alphaproteobacteria Rhizobiales *Rhizobiaceae Aquamicrobium*

Otu00761 -6.1 9.E-06 Pro/Gammaproteobacteria Betaproteobacteriales *Burkholderiaceae Herminiimonas*

Otu02077 -6.1 3.E-04 Arm/Fimbriimonadia Fimbriimonadales *Fimbriimonadaceae* unknown

Otu04557 -6.1 5.E-04 Pro/Deltaproteobacteria Myxococcales unknown unknown

Otu04562 -6.2 2.E-04 Act/Actinobacteria Micrococcales Cellulom*onadaceae Oerskovia*

Otu03827 -6.3 9.E-06 Pro/Gammaproteobacteria Betaproteobacteriales *Burkholderiaceae Candidimonas*

Otu07436 -6.4 2.E-04 Ver/Verrucomicrobiae Pedosphaerales *Pedosphaeraceae* unknown

Otu07317 -6.5 2.E-04 Pro/Gammaproteobacteria Pseudomonadales *Moraxellaceae* unknown

Otu04159 -6.5 8.E-05 Pla/Planctomycetacia Planctomycetales unknown unknown

Otu02002 -6.5 5.E-05 Pro/Alphaproteobacteria Rhizobiales *Devosiaceae Devosia*

Otu04163 -6.5 5.E-05 Pla/Planctomycetacia Planctomycetales *Rubinisphaeraceae* unknown

Otu00594 -6.7 3.E-05 Pat/Parcubacteria Cand_Zambryskibacteria unknown unknown

Otu03849 -6.7 1.E-05 Bact/Bacteroidia Chitinophagales *Chitinophagaceae Ferruginibacter*

Otu03936 -6.7 3.E-04 Pla/Planctomycetacia Pirellulales *Pirellulaceae Pirellula*

Otu03914 -6.7 2.E-04 Bact/Bacteroidia Chitinophagales *Chitinophagaceae Ferruginibacter*

**Otu00638 -6.7 9.E-16 Bact/Bacteroidia Chitinophagales *Chitinophagaceae* unknown**

Otu01271 -6.8 4.E-05 Bact/Bacteroidia Bacteroidales unknown unknown

Otu01997 -6.8 8.E-06 Pro/Deltaproteobacteria Myxococcales *Sandaracinaceae* unknown

Otu04067 -6.9 5.E-05 Pla/Planctomycetacia Pirellulales *Pirellulaceae Pirellula*

Otu03818 -6.9 7.E-04 Pro/Deltaproteobacteria Myxococcales *Sandaracinaceae Sandaracinus*

Otu07995 -6.9 5.E-05 Pla/Planctomycetacia Pirellulales *Pirellulaceae Rhodopirellula*

Otu01025 -6.9 2.E-07 Ver/Verrucomicrobiae Chthoniobacterales *Chthoniobacteraceae Chthoniobacter*

Otu04217 -7.0 1.E-05 Bact/Bacteroidia Chitinophagales *Chitinophagaceae* unknown

Otu01987 -7.0 5.E-06 Pro/Alphaproteobacteria Micavibrionales unknown unknown

Otu04582 -7.0 4.E-06 Pro/Gammaproteobacteria Pseudomonadales *Moraxellaceae* unknown

Otu03952 -7.1 4.E-04 Pla/Planctomycetacia Pirellulales *Pirellulaceae Pirellula*

Otu04022 -7.2 1.E-05 Pat/Berkelbacteria unknown unknown unknown

Otu03838 -7.5 1.E-05 Bact/Bacteroidia Chitinophagales *Chitinophagaceae Taibaiella*

Otu00763 -7.6 1.E-06 Chlo/Anaerolineae unknown unknown unknown

Otu01404 -7.6 3.E-06 Gem/Gemmatimonadetes Gemmatimonadales *Gemmatimonadaceae Gemmatimonas*

Otu03950 -7.7 3.E-06 Pro/Alphaproteobacteria Micavibrionales unknown unknown

Otu01026 -7.7 1.E-07 Bact/Bacteroidia Flavobacteriales *Weeksellaceae Chryseobacterium*

Otu03976 -7.9 3.E-08 Pro/Alphaproteobacteria Rhizobiales *Beijerinckiaceae* unknown

Otu02080 -8.0 2.E-08 Bact/Bacteroidia Flavobacteriales unknown unknown

Otu01176 -8.1 3.E-08 Pro/Gammaproteobacteria Xanthomonadales *Rhodanobacteraceae Rhodanobacter*

Otu00894 -8.3 2.E-09 Pro/Alphaproteobacteria Caulobacterales *Caulobacteraceae Asticcacaulis*

Otu02034 -8.7 1.E-07 Pro/Gammaproteobacteria Xanthomonadales *Xanthomonadaceae Thermomonas*

Otu00786 -8.7 4.E-10 Bact/Bacteroidia Cytophagales *Microscillaceae* unknown

Otu01273 -9.1 2.E-10 Pro/Gammaproteobacteria Xanthomonadales *Rhodanobacteraceae Dokdonella*

Otu02219 -9.3 2.E-09 Pla/Planctomycetacia Planctomycetales *Schlesneriaceae Planctopirus*

Otu02033 -9.5 1.E-10 Pro/Gammaproteobacteria Xanthomonadales *Xanthomonadaceae Pseudoxanthomonas*

Otu01288 -9.6 5.E-09 Pat/unknown unknown unknown unknown

**Otu00773 -9.7 2.E-11 Chlo/Anaerolineae Anaerolineales *Anaerolineaceae* unknown**

Otu04567 -21.5 4.E-08 Bact/Bacteroidia Sphingobacteriales *Sphingobacteriaceae Sphingobacterium*

Otu01060 -21.9 2.E-08 Pro/Gammaproteobacteria Betaproteobacteriales *Rhodocyclaceae Dechloromonas*

^________________________________________________________________________________________________________________________________________________________________________________________________________^

1) Including also genera *Caballeronia* and *Paraburkholderia,* 2) Including also genera *Neorhizobium, Pararhizobium* and *Rhizobium*

**Table S5.** Results from the differential abundance analysis that shows all significant (*P* adj <0.001) fungal ITS derived indicative OTUs with their taxonomic affiliations. Log2foldChange (l2FC) values are from paired comparison between pure control soils (clay or silt) with four different bark-derived amendments (B, BH, BA, BHA). Results are shown for the clay and silt soil, and for the 2^nd^ and 3^rd^ samplings separately. Positive values refer to OTUs indicative for controls soils and negative for the microcosms with organic amendments. Abbreviations: As, Ascomycota; Ba, Basidiomycota; Gl, Glomeromycota; Mo, Mortierellomycota; Mu, Mucoromyocta; Ro, Rozellomycota; fIs; family Insertae sedis; C, control; B, bark, BH, hot-water extracted bark; BA, bark from anaerobic digestion process; BHA, hot-water extracted bark from anaerobic digestion. Bolded are represented in heatmap figure 4.

2^nd^ sampling OTU_ID l2FC *P* adj Phylum/Class Family Genus Species

^________________________________________________________________________________________________________________________________________________________________________________________________________^

clay

C vs B Otu3135 7.7 3.E-06 As/Leotiomycetes Vibrisseaceae *Phialocephala P. humicola*

Otu3510 4.7 6.E-05 As/Leotiomycetes unidentified unidentified

Otu2767 -2.1 2.E-05 As/Sordariomycetes Nectriaceae *Dactylonectria D. macrodidyma*

Otu3364 -2.4 9.E-06 As/Leotiomycetes Helotiaceae *Hymenoscyphus H. kathiae*

Otu1557 -3.4 1.E-05 Ba/Microbotryomycetes Chrysozymaceae *Slooffia S. cresolica*

Otu2293 -5.3 1.E-05 As/Sordariomycetes unidentified unidentified

Otu3145 -5.8 1.E-06 As/Leotiomycetes unidentified unidentified

Otu2027 -5.9 2.E-04 As/Saccharomycetes unidentified unidentified

Otu2770 -6.2 3.E-04 As/Eurotiomycetes unidentified unidentified

Otu3459 -6.6 3.E-05 As/Dothideomycetes unidentified unidentified

Otu2715 -6.6 1.E-05 As/Eurotiomycetes unidentified unidentified

Otu2581 -7.0 7.E-07 As/Orbiliomycetes Orbiliaceae *Arthrobotrys A. elegans*

Otu2794 -7.8 5.E-04 Ba/Tremellomycetes unidentified unidentified

Otu2183 -8.0 1.E-08 As/Saccharomycetes unidentified *Candida C. santamariae*

Otu3387 -8.4 1.E-03 As/unidentified unidentified unidentified

Otu3389 -8.5 8.E-05 As/Leotiomycetes Hyaloscyphaceae *Ciliolarina*

Otu3440 -8.6 5.E-09 As/Leotiomycetes unidentified *Chalara*

Otu1651 -8.7 7.E-06 Ba/Microbotryomycetes Chrysozymaceae *Hamamotoa H. lignophila*

Otu1230 -8.9 3.E-09 Ba/Tremellomycetes Cystofilobasidiaceae *Cystofilobasidium C. capitatum*

Otu0923 -9.0 3.E-04 Ba/Microbotryomycetes Sporidiobolaceae *Rhodotorula*

Otu3353 -9.3 1.E-08 As/Leotiomycetes Dermateaceae *Cryptosporiopsis*

Otu2543 -9.4 2.E-07 As/Eurotiomycetes unidentified unidentified

**Otu2748 -9.5 2.E-10 As/Eurotiomycetes Herpotrichiellaceae unidentified**

Otu3317 -9.5 4.E-09 As/Leotiomycetes Helotiales FIs *Xenochalara*

Otu2486 -9.8 2.E-05 As/Orbiliomycetes Orbiliaceae unidentified

Otu2035 -9.8 9.E-05 As/Saccharomycetes Pichiaceae *Nakazawaea N. holstii*

Otu2678 -9.8 3.E-06 As/Orbiliomycetes Orbiliaceae *Arthrobotrys A. vermicola*

Otu1097 -9.9 1.E-07 Ba/Agaricomycetes Cantharellales FIs *Minimedusa M. polyspora*

Otu2679 -10.4 7.E-07 As/Eurotiomycetes Aspergillaceae *Penicillium P. xanthomelinii*

Otu2421 -10.5 1.E-05 As/Dothideomycetes unidentified unidentified

Otu2272 -10.6 1.E-06 Ba/Agaricomycetes Serendipitaceae unidentified

Otu2074 -11.3 3.E-06 Ba/Agaricomycetes Entolomataceae *Clitopilus C. hobsonii*

Otu3149 -11.6 4.E-08 As/Leotiomycetes Helotiaceae *Collophora*

**Otu1847 -12.8 7.E-14 As/Saccharomycetes Debaryomycetaceae *Peterozyma P. toletana***

Otu2089 -12.9 6.E-08 As/Saccharomycetes Pichiaceae *Nakazawaea N. holstii*

**Otu3281 -13.5 7.E-10 As/Leotiomycetes Helotiales FIs *Xenopolyscytalum X. pinea***

Otu1598 -22.9 1.E-09 Mu/Mucoromycetes Mucoraceae *Mucor M. piriformis*

Otu1483 -24.8 3.E-11 Ba/Agaricomycetes unidentified unidentified

**Otu2012 -25.2 5.E-12 Mu/Mucoromycetes Mucoraceae *Mucor M. strictus***

**Otu1400 -25.5 7.E-12 As/Sordariomycetes Ophiostomataceae *Ceratocystiopsis C. minuta***

Otu3319 -25.7 5.E-12 As/Leotiomycetes unidentified unidentified

**Otu2988 -25.7 5.E-12 As/Leotiomycetes Pseudeurotiaceae unidentified**

**Otu2324 -26.3 7.E-14 Mu/Mucoromycetes Mucoraceae *Mucor M. racemosus***

**Otu1471 -26.9 7.E-13 Ba/Agaricomycetes Cantharellales FIs unidentified**

^________________________________________________________________________________________________________________________________________________________________________________________________________^

silt

C vs B Otu1097 -3.7 8.E-04 Ba/Agaricomycetes Cantharellales FIs *Minimedusa M. polyspora*

Otu3145 -5.3 8.E-07 As/Leotiomycetes unidentified unidentified

Otu1900 -5.7 8.E-04 Ba/Agaricomycetes Pleurotaceae unidentified

Otu1693 -6 3.E-14 Ba/Microbotryomycetes unidentified unidentified

Otu1612 -6.6 8.E-04 Ba/Atractiellomycetes unidentified unidentified

Otu2679 -6.6 3.E-05 As/Eurotiomycetes Aspergillaceae *Penicillium P. xanthomelinii*

Otu2183 -6.7 1.E-05 As/Saccharomycetes Saccharomycetales FIs *Candida C. santamariae*

**Otu2341 -8 3.E-35 As/Sordariomycetes Niessliaceae *Eucasphaeria***

**Otu1651 -8 3.E-07 Ba/Microbotryomycetes Chrysozymaceae** *Hamamotoa H. lignophila*

Otu2284 -8.4 4.E-04 As/Saccharomycetes Saccharomycetales FIs *Kuraishia K. capsulata*

Otu1230 -8.5 2.E-06 Ba/Tremellomycetes Cystofilobasidiaceae *Cystofilobasidium C. capitatum*

Otu2272 -8.9 3.E-05 Ba/Agaricomycetes Serendipitaceae unidentified

Otu2678 -9.3 9.E-07 As/Orbiliomycetes Orbiliaceae *Arthrobotrys A. vermicola*

Otu2293 -9.4 8.E-07 As/Sordariomycetes unidentified unidentified

**Otu3317 -10 5.E-08 As/Leotiomycetes Helotiales FIs *Xenochalara***

Otu3149 -10.0 5.E-05 As/Leotiomycetes Helotiaceae *Collophora*

Otu2089 -11.6 8.E-05 As/Saccharomycetes Pichiaceae *Nakazawaea N. holstii*

**Otu1847 -12 9.E-14 As/Saccharomycetes Debaryomycetaceae *Peterozyma P. toletana***

**Otu3281 -13 1.E-07 As/Leotiomycetes Helotiales FIs *Xenopolyscytalum X. pinea***

**Otu1298 -21 6.E-08 Ba/Agaricomycetes Cantharellales FIs *Sistotrema***

**Otu2029 -21 2.E-08 Ba/Agaricomycetes Tricholomataceae *Calyptella C. capula***

Otu1084 -22 7.E-09 Ba/Agaricomycetes Ceratobasidiaceae *Thanatephorus T. cucumeris*

**Otu1122 -25 2.E-11 Ba/Agaricomycetes Ceratobasidiaceae *Thanatephorus T. cucumeris***

^________________________________________________________________________________________________________________________________________________________________________________________________________^

clay

C vs BH

Otu3135 7.2 4.E-05 As/Leotiomycetes Vibrisseaceae *Phialocephala P. humicola*

Otu3365 -2.1 1.E-05 As/Leotiomycetes unidentified unidentified

Otu2675 -3.0 8.E-04 As/Sordariomycetes Nectriaceae *Ilyonectria I. mors-panacis*

Otu3364 -3.6 3.E-05 As/Leotiomycetes Helotiaceae *Hymenoscyphus H. kathiae*

Otu1557 -5.2 1.E-05 Ba/Microbotryomycetes Chrysozymaceae *Slooffia S. cresolica*

Otu3145 -5.9 1.E-07 As/Leotiomycetes unidentified unidentified

Otu2293 -6.1 7.E-04 As/Sordariomycetes unidentified unidentified

Otu2035 -6.1 5.E-04 As/Saccharomycetes Pichiaceae *Nakazawaea N. holstii*

Otu3495 -6.4 4.E-06 As/Leotiomycetes Hyaloscyphaceae *Hyphodiscus H. stereicola*

Otu2581 -6.7 6.E-06 As/Orbiliomycetes Orbiliaceae *Arthrobotrys A. elegans*

Otu3207 -6.7 1.E-04 As/unidentified unidentified unidentified

Otu1612 -7.2 9.E-08 Ba/Atractiellomycetes unidentified unidentified

Otu2742 -7.4 4.E-05 As/Eurotiomycetes Aspergillaceae *Penicillium P. bialowiezense*

Otu3252 -7.6 4.E-05 As/Leotiomycetes unidentified unidentified

Otu3230 -7.7 3.E-04 As/unidentified unidentified unidentified

Otu2744 -7.8 4.E-05 As/Dothideomycetes Dothioraceae *Hormonema H. macrosporum*

Otu2584 -8.1 6.E-04 As/Eurotiomycetes unidentified unidentified

Otu2183 -8.3 2.E-04 As/Saccharomycetes Saccharomycetales *FIs* *Candida C. santamariae*

Otu3353 -8.5 4.E-07 As/Leotiomycetes Dermateaceae *Cryptosporiopsis*

Otu1097 -8.6 2.E-04 Ba/Agaricomycetes Cantharellales fIs *Minimedusa M. polyspora*

Otu2679 -8.8 5.E-05 As/Eurotiomycetes Aspergillaceae *Penicillium P. xanthomelinii*

Otu2794 -9.1 3.E-06 Ba/Tremellomycetes unidentified unidentified

Otu1122 -9.1 6.E-05 Ba/Agaricomycetes Ceratobasidiaceae *Thanatephorus T. cucumeris*

Otu1441 -9.4 2.E-11 Mu/Mucoromycetes Mucoraceae *Mucor M. piriformis*

Otu1863 -9.4 8.E-07 Ba/Agaricomycetes Serendipitaceae *Serendipita*

Otu2284 -9.5 2.E-08 As/Saccharomycetes Saccharomycetales fIs *Kuraishia K. capsulata*

Otu1230 -9.6 3.E-07 Ba/Tremellomycetes Cystofilobasidiaceae *Cystofilobasidium C. capitatum*

Otu3281 -9.7 5.E-05 As/Leotiomycetes Helotiales fIs *Xenopolyscytalum X. pinea*

Otu0923 -10.0 5.E-07 Ba/Microbotryomycetes Sporidiobolaceae *Rhodotorula*

Otu3572 -10.0 1.E-06 As/Leotiomycetes Myxotrichaceae *Oidiodendron O. rhodogenum*

**Otu2486 -10.0 1.E-09 As/Orbiliomycetes Orbiliaceae unidentified**

Otu2828 -10.6 1.E-07 As/Eurotiomycetes Aspergillaceae *Penicillium P. thomii*

Otu3149 -10.8 1.E-07 As/Leotiomycetes Helotiaceae *Collophora*

Otu1526 -10.9 7.E-07 Ba/Agaricomycetes unidentified unidentified

Otu2421 -11.5 2.E-11 As/Dothideomycetes unidentified unidentified

**Otu2678 -11.6 6.E-11 As/Orbiliomycetes Orbiliaceae *Arthrobotrys A. vermicola***

Otu2543 -11.8 3.E-05 As/Eurotiomycetes unidentified unidentified

**Otu1613 -12.1 7.E-14 As/Sordariomycetes Ophiostomataceae *Pesotum P. piceae***

**Otu2324 -12.3 2.E-14 Mu/Mucoromycetes Mucoraceae *Mucor M. racemosus***

**Otu2089 -12.6 6.E-15 As/Saccharomycetes Pichiaceae *Nakazawaea N. holstii***

**Otu2272 -12.7 1.E-09 Ba/Agaricomycetes Serendipitaceae unidentified**

**Otu1847 -13.0 9.E-15 As/Saccharomycetes Debaryomycetaceae *Peterozyma P. toletana***

Otu1403 -21.5 1.E-08 Ba/Agaricomycetes Ceratobasidiaceae *Ceratobasidium*

Otu3463 -22.2 4.E-09 As/Dothideomycetes Mycosphaerellaceae *Septoria S. cerastii*

**Otu2074 -24.8 3.E-11 Ba/Agaricomycetes Entolomataceae *Clitopilus C. hobsonii***

^________________________________________________________________________________________________________________________________________________________________________________________________________^

silt

C vs BH Otu2651 -2.6 1.E-05 As/Sordariomycetes unidentified unidentified

Otu1693 -3.6 6.E-05 Ba/Microbotryomycetes unidentified unidentified

Otu3207 -3.8 4.E-07 As/unidentified unidentified unidentified

Otu2627 -4.2 5.E-05 As/Eurotiomycetes Aspergillaceae *Penicillium P. solitum*

Otu3364 -4.3 2.E-06 As/Leotiomycetes Helotiaceae *Hymenoscyphus H. kathiae*

Otu2514 -5.5 8.E-05 As/Orbiliomycetes Orbiliaceae unidentified

Otu2679 -5.7 5.E-05 As/Eurotiomycetes Aspergillaceae *Penicillium P. xanthomelinii*

Otu3145 -7 5.E-25 As/Leotiomycetes unidentified unidentified

**Otu2341 -7 2.E-11 As/Sordariomycetes Niessliaceae *Eucasphaeria***

Otu2065 -7.1 3.E-04 Ba/Agaricomycetes unidentified unidentified

Otu2293 -7.1 3.E-06 As/Sordariomycetes unidentified unidentified

Otu1612 -7.2 4.E-05 Ba/Atractiellomycetes unidentified unidentified

Otu2460 -7.3 9.E-06 As/Pezizomycetes Pezizaceae *Peziza*

Otu1424 -7.4 3.E-04 Ba/Agaricomycetes Ceratobasidiaceae *Rhizoctonia*

Otu2742 -7.9 6.E-05 As/Eurotiomycetes Aspergillaceae *Penicillium P. bialowiezense*

Otu0626 -8.5 4.E-06 Mo/Mortierellomycetes Mortierellaceae *Mortierella M. angusta*

**Otu2272 -8.9 1.E-09 Ba/Agaricomycetes Serendipitaceae unidentified**

Otu2284 -9.2 8.E-06 As/Saccharomycetes Saccharomycetales FIs *Kuraishia K. capsulata*

**Otu1441 -9.3 3.E-09 Mu/Mucoromycetes Mucoraceae *Mucor M. piriformis***

Otu3149 -10.0 3.E-07 As/Leotiomycetes Helotiaceae Collophora

**Otu1613 -10 2.E-11 As/Sordariomycetes Ophiostomataceae *Pesotum P. piceae***

Otu1230 -10.0 3.E-06 Ba/Tremellomycetes Cystofilobasidiaceae *Cystofilobasidium C. capitatum*

**Otu2678 -10 5.E-14 As/Orbiliomycetes Orbiliaceae *Arthrobotrys A. vermicola***

**Otu2289 -10.6 1.E-08 Ba/Agaricomycetes Serendipitaceae unidentified**

**Otu2324 -11 2.E-17 Mu/Mucoromycetes Mucoraceae *Mucor M. racemosus***

**Otu2089 -13 2.E-11 As/Saccharomycetes Pichiaceae *Nakazawaea N. holstii***

**Otu1847 -14 6.E-15 As/Saccharomycetes Debaryomycetaceae *Peterozyma P. toletana***

^________________________________________________________________________________________________________________________________________________________________________________________________________^

clay

C vs BA **Otu0600 8.4 1.E-10 Ba/Agaricomycetes Tricholomataceae *Lachnella L. villosa***

**Otu3135 7.1 5.E-05 As/Leotiomycetes Vibrisseaceae *Phialocephala P. humicola***

Otu3510 4.9 4.E-06 As/Leotiomycetes unidentified unidentified

Otu2507 2.6 4.E-04 unidentified unidentified unidentified

Otu2704 -3.1 2.E-05 As/unidentified unidentified unidentified

**Otu2495 -18.9 2.E-07 As/Sordariomycetes Bionectriaceae unidentified**

Otu3121 -22.0 3.E-08 As/Leotiomycetes unidentified unidentified

^________________________________________________________________________________________________________________________________________________________________________________________________________^

silt

C vs BA **Otu2113 6.7 2.E-05 A/Eurotiomycetes Herpotrichiellaceae unidentified**

Otu2216 -2.3 1.E-04 unidentified unidentified unidentified

Otu3645 -2.4 4.E-16 R/Rozellomycotina unidentified unidentified

**Otu1989 -9.8 9.E-04 Ba/Agaricomycetes Bolbitiaceae *Conocybe C. anthracophila***

^________________________________________________________________________________________________________________________________________________________________________________________________________^

clay

C vs BHA **Otu3135 7 1.E-04 As/Leotiomycetes Vibrisseaceae *Phialocephala P. humicola***

**Otu3226 -2 4.E-05 As/Eurotiomycetes Onygenales fIs *Chrysosporium C. pseudomerdarium***

Otu2704 -3 7.E-04 As/unidentified unidentified unidentified

**Otu1900 -8 6.E-07 Ba/Agaricomycetes Pleurotaceae unidentified**

^________________________________________________________________________________________________________________________________________________________________________________________________________^

silt

C vs BHA Otu1706 2.5 2.E-04 Ba/Microbotryomycetes unidentified unidentified

Otu3645 -2.3 9.E-06 Ro/Rozellomycotina unidentified unidentified

Otu2216 -2.4 4.E-04 unidentified unidentified unidentified

Otu3220 -20.3 2.E-06 A/Leotiomycetes unidentified unidentified

^________________________________________________________________________________________________________________________________________________________________________________________________________^

3rd sampling

^________________________________________________________________________________________________________________________________________________________________________________________________________^

clay

C vs B

Otu1768 8.1 1.E-07 A/Sordariomycetes unidentified unidentified

Otu2499 5.4 5.E-07 As/Sordariomycetes Nectriaceae *Fusarium F.culmorum*

Otu2440 4.5 5.E-04 As/Sordariomycetes Lasiosphaeriaceae *Podospora*

Otu2754 -3.0 6.E-05 As/Leotiomycetes unidentified unidentified

Otu2792 -3.3 7.E-08 As/Leotiomycetes Helotiaceae *Hymenoscyphus H. kathiae*

Otu1277 -4.5 3.E-07 Ba/Microbotryomycetes Chrysozymaceae *Slooffia S. cresolica*

Otu0993 -6.6 1.E-05 Ba/Tremellomycetes Cystofilobasidiaceae *Cystofilobasidium C. capitatum*

Otu1771 -6.6 3.E-06 As/Saccharomycetes Saccharomycetales FIs *Candida C. santamariae*

Otu1862 -7.1 3.E-06 As/Sordariomycetes unidentified unidentified

Otu1287 -7.4 7.E-04 Ba/Atractiellomycetes unidentified unidentified

Otu1359 -7.4 6.E-07 Ba/Microbotryomycetes Chrysozymaceae *Hamamotoa H. lignophila*

Otu2208 -7.6 2.E-07 As/Orbiliomycetes Orbiliaceae *Arthrobotrys A. vermicola*

Otu0729 -7.7 7.E-05 Ba/Microbotryomycetes Sporidiobolaceae *Rhodotorula*

Otu0777 -7.8 6.E-05 Mu/Endogonomycetes unidentified unidentified

Otu2094 -7.9 7.E-06 As/Eurotiomycetes unidentified unidentified

Otu0836 -8.2 3.E-04 Ba/Agaricomycetes Cantharellales FIs *Minimedusa M. polyspora*

Otu2109 -8.3 7.E-06 As/Orbiliomycetes Orbiliaceae *Arthrobotrys A. elegans*

Otu2203 -8.6 7.E-08 As/Eurotiomycetes Aspergillaceae *Penicillium P. xanthomelinii*

Otu1465 -8.7 1.E-03 Ba/Agaricomycetes Serendipitaceae unidentified

Otu2270 -8.7 7.E-07 As/Eurotiomycetes Herpotrichiellaceae unidentified

Otu1658 -9.0 3.E-04 Ba/Agaricomycetes Entolomataceae *Clitopilus*

Otu2758 -9.6 2.E-05 As/Leotiomycetes Helotiales FIs *Xenochalara*

Otu1703 -9.7 6.E-06 As/Saccharomycetes Pichiaceae *Nakazawaea N. holstii*

Otu2935 -9.9 2.E-05 As/Leotiomycetes Myxotrichaceae *Oidiodendron O. echinulatum*

Otu1983 -10.1 7.E-07 As/Dothideomycetes unidentified unidentified

Otu1887 -10.2 3.E-04 Mu/Mucoromycetes Mucoraceae *Mucor M. racemosus*

**Otu2008 -10.2 1.E-10 As/Orbiliomycetes Orbiliaceae unidentified**

Otu1830 -10.5 7.E-05 Ba/Agaricomycetes Serendipitaceae unidentified

Otu2589 -10.8 1.E-06 As/Leotiomycetes Helotiaceae *Collophora*

Otu2838 -11.1 3.E-06 As/Leotiomycetes Dermateaceae *Cryptosporiopsis*

Otu0242 -11.5 5.E-04 Ba/Agaricomycetes Omphalotaceae *Omphalotus O. mexicanus*

**Otu1498 -12.2 8.E-12 As/Saccharomycetes Debaryomycetaceae *Peterozyma P. toletana***

**Otu2728 -12.6 1.E-10 As/Leotiomycetes Helotiales FIs *Xenopolyscytalum X. pinea***

**Otu1675 -13.2 8.E-12 Ba/Agaricomycetes Entolomataceae *Clitopilus C. hobsonii***

Otu2652 -20.9 3.E-08 As/Leotiomycetes Helotiales Fls *Cadophora C. melinii*

Otu0250 -21.4 1.E-08 Ba/Agaricomycetes Omphalotaceae *Omphalotus O. illudens*

Otu2134 -21.9 6.E-09 As/Orbiliomycetes Orbiliaceae *Orbilia O. rectispora*

Otu2739 -22.9 8.E-10 As/Leotiomycetes unidentified unidentified

Otu2785 -23.1 6.E-10 As/Leotiomycetes Hyaloscyphaceae *Ciliolarina*

**Otu0908 -23.6 2.E-10 Ba/Agaricomycetes Ceratobasidiaceae unidentified**

**Otu2746 -23.8 1.E-10 As/Leotiomycetes Helotiales FIs *Chalara C. holubovae***

**Otu1640 -23.9 1.E-10 Mu/Mucoromycetes Mucoraceae *Mucor M. strictus***

**Otu1523 -24.2 1.E-10 Ba/Agaricomycetes Serendipitaceae Serendipita**

**Otu1163 -24.8 5.E-11 Ba/Agaricomycetes Boletales FIs *Hydnomerulius H. pinastri***

**Otu1189 -25.6 1.E-11 Ba/Agaricomycetes Cantharellales FIs unidentified**

^________________________________________________________________________________________________________________________________________________________________________________________________________^

silt

control vs B

**Otu2029 21.7 4.E-09 As/Sordariomycetes Hypocreaceae *Trichoderma T. ivoriense***

Otu2792 -4.2 2.E-04 As/Leotiomycetes Helotiaceae *Hymenoscyphus H. kathiae*

Otu2669 -5.0 9.E-07 As/Leotiomycetes unidentified unidentified

Otu2208 -6.3 4.E-05 As/Orbiliomycetes Orbiliaceae *Arthrobotrys A. vermicola*

Otu1862 -7.2 4.E-05 As/Sordariomycetes unidentified unidentified

Otu1370 -7.4 2.E-06 Ba/Microbotryomycetes unidentified unidentified

**Otu1913 -7.4 2.E-26 As/Sordariomycetes Niessliaceae *Eucasphaeria***

**Otu1498 -8.9 2.E-08 As/Saccharomycetes Debaryomycetaceae *Peterozyma P. toletana***

Otu1830 -9.4 3.E-07 Ba/Agaricomycetes Serendipitaceae unidentified

Otu1287 -10.9 3.E-06 Ba/Atractiellomycetes unidentified unidentified

Otu2728 -11.6 1.E-06 As/Leotiomycetes Helotiales FIs *Xenopolyscytalum X. pinea*

**Otu2932 -21.5 1.E-08 As/Leotiomycetes Myxotrichaceae *Oidiodendron O. rhodogenum***

**Otu2619 -21.6 1.E-08 As/Sordariomycetes Lasiosphaeriaceae unidentified**

**Otu1636 -21.7 9.E-09 Ba/Agaricomycetes Bolbitiaceae *Conocybe C. semiglobata***

**Otu2281 -22.1 5.E-09 As/Pezizomycetes Pyronemataceae unidentified**

**Otu2761 -22.4 4.E-09 As/Leotiomycetes Helotiaceae *Tetracladium***

**Otu2027 -22.5 4.E-09 As/Orbiliomycetes Orbiliaceae unidentified**

**Otu1887 -25.0 8.E-14 Mu/Mucoromycetes Mucoraceae *Mucor M. racemosus***

^________________________________________________________________________________________________________________________________________________________________________________________________________^

clay

C vs BH Otu1768 5.7 5.E-05 As/Sordariomycetes unidentified unidentified

Otu2010 4.0 5.E-04 As/Sordariomycetes Hypocreales FIs *Acremonium A. persicinum*

Otu2499 4.0 8.E-05 As/Sordariomycetes Nectriaceae *Fusarium F. culmorum*

Otu2419 2.9 4.E-04 As/Sordariomycetes Chaetomiaceae *Chaetomidium C. gallecicum*

Otu2451 -3.0 4.E-06 As/Sordariomycetes Chaetomiaceae *Chaetomium*

Otu2792 -3.6 3.E-08 As/Leotiomycetes Helotiaceae *Hymenoscyphus H. kathiae*

Otu1277 -3.7 8.E-07 Ba/Microbotryomycetes Chrysozymaceae *Slooffia S. cresolica*

Otu2754 -4.0 2.E-06 As/Leotiomycetes unidentified unidentified

Otu2851 -6.0 2.E-06 As/Leotiomycetes Hyaloscyphaceae *Hyphodiscus H. stereicola*

Otu0993 -6.6 2.E-04 Ba/Tremellomycetes Cystofilobasidiaceae *Cystofilobasidium C. capitatum*

Otu2660 -6.8 4.E-04 As/unidentified unidentified unidentified

Otu1849 -7.1 2.E-06 As/Saccharomycetes Saccharomycetales FIs *Kuraishia K. capsulata*

Otu2277 -7.4 3.E-04 As/Eurotiomycetes Aspergillaceae *Penicillium P. bialowiezense*

Otu0729 -7.4 7.E-07 Ba/Microbotryomycetes Sporidiobolaceae *Rhodotorula*

Otu2318 -7.4 4.E-06 Ba/Tremellomycetes unidentified unidentified

Otu1862 -7.6 4.E-07 As/Sordariomycetes unidentified unidentified

Otu2203 -7.9 1.E-04 As/Eurotiomycetes Aspergillaceae *Penicillium P. xanthomelinii*

Otu1287 -8.3 4.E-08 Ba/Atractiellomycetes unidentified unidentified

Otu2109 -8.6 3.E-08 As/Orbiliomycetes Orbiliaceae *Arthrobotrys A. elegans*

Otu1181 -8.7 1.E-09 Mu/Mucoromycetes Mucoraceae *Mucor M. piriformis*

Otu0777 -9 6.E-15 Mu/Endogonomycetes unidentified unidentified

Otu0927 -9.1 1.E-05 Ba/Agaricomycetes Ceratobasidiaceae *Thanatephorus T. cucumeris*

Otu2589 -9.3 4.E-07 As/Leotiomycetes Helotiaceae *Collophora*

Otu2208 -9.4 1.E-06 As/Orbiliomycetes Orbiliaceae *Arthrobotrys A. vermicola*

**Otu1703 -9 4.E-10 As/Saccharomycetes Pichiaceae *Nakazawaea N. holstii***

Otu2838 -10.0 1.E-05 As/Leotiomycetes Dermateaceae *Cryptosporiopsis*

Otu1254 -10.0 3.E-08 Ba/Agaricomycetes unidentified unidentified

Otu2932 -10.6 5.E-05 As/Leotiomycetes Myxotrichaceae *Oidiodendron O. rhodogenum*

**Otu2008 -11 5.E-15 As/Orbiliomycetes Orbiliaceae unidentified**

**Otu1830 -11 2.E-10 Ba/Agaricomycetes Serendipitaceae unidentified**

Otu1296 -10.9 1.E-09 As/Sordariomycetes Ophiostomataceae *Pesotum P. piceae*

Otu0242 -10.9 4.E-04 Ba/Agaricomycetes Omphalotaceae *Omphalotus O. mexicanus*

Otu1983 -11 8.E-12 As/Dothideomycetes unidentified unidentified

**Otu1887 -11 4.E-11 Mu/Mucoromycetes Mucoraceae *Mucor M. racemosus***

**Otu1498 -12 5.E-14 As/Saccharomycetes Debaryomycetaceae *Peterozyma P. toletana***

Otu1675 -12.7 7.E-04 Ba/Agaricomycetes Entolomataceae *Clitopilus C. hobsonii*

Otu1523 -13.0 1.E-07 Ba/Agaricomycetes Serendipitaceae *Serendipita*

**Otu0908 -13 7.E-11 Ba/Agaricomycetes Ceratobasidiaceae unidentified**

Otu2169 -21.5 1.E-08 As/Sordariomycetes Hypocreales FIs *Acremonium*

Otu2747 -21.9 7.E-09 As/Leotiomycetes Hyaloscyphaceae *Gyoerffyella*

**Otu2198 -24 2.E-10 As/Sordariomycetes Clavicipitaceae *Paecilomyces P. penicillatus***

Otu1243 -25 3.E-11 Ba/Agaricomycetes unidentified unidentified

^________________________________________________________________________________________________________________________________________________________________________________________________________^

silt

C vs BH Otu1887 7 1.E-11 Ba/Agaricomycetes unidentified unidentified

Otu2208 -2.8 2.E-04 Ba/Microbotryomycetes unidentified unidentified

Otu2669 -4 2.E-10 As/Leotiomycetes Helotiaceae *Hymenoscyphus H. kathiae*

Otu0777 -4 2.E-13 As/Leotiomycetes unidentified unidentified

Otu1913 -4.1 6.E-04 Ba/GS27 unidentified unidentified

Otu1830 -4.4 2.E-05 As/Sordariomycetes unidentified unidentified

**Otu1667 -5 8.E-12 As/Sordariomycetes Niessliaceae *Eucasphaeria***

Otu1498 -6.4 5.E-05 As/Eurotiomycetes Aspergillaceae *Penicillium P. bialowiezense*

Otu2792 -6.5 1.E-03 Ba/Tremellomycetes Cystofilobasidiaceae *Cystofilobasidium C. capitatum*

**Otu1259 -7 2.E-13 As/Orbiliomycetes Orbiliaceae *Arthrobotrys A. vermicola***

Otu1296 -7.1 8.E-04 As/Pezizomycetes Pezizaceae Peziza

Otu1181 -7.7 3.E-05 As/Saccharomycetes Saccharomycetales FIs *Kuraishia K. capsulata*

**Otu2115 -8 1.E-11 Ba/Agaricomycetes Serendipitaceae unidentified**

Otu1703 -7.8 2.E-08 Mu/Mucoromycetes Mucoraceae *Mucor M. piriformis*

Otu1853 -8.7 8.E-09 As/Sordariomycetes Ophiostomataceae *Pesotum P. piceae*

Otu1862 -9.1 6.E-07 As/Saccharomycetes Pichiaceae *Nakazawaea N. holstii*

Otu1287 -9.2 5.E-05 Mo/Mortierellomycetes Mortierellaceae *Mortierella M. angusta*

**Otu1849 -10 1.E-10 As/Saccharomycetes Debaryomycetaceae *Peterozyma P. toletana***

Otu2277 -9.6 1.E-06 Ba/Agaricomycetes Serendipitaceae unidentified

**Otu0464 -11 3.E-15 Mu/Mucoromycetes Mucoraceae *Mucor M. racemosus***

Otu1413 -13.0 2.E-05 Ba/Atractiellomycetes unidentified unidentified

Otu1045 -21.1 2.E-08 As/Dothideomycetes Pleosporaceae *Bipolaris B. sorokiniana*

**Otu2024 -22.2 4.E-09 Ba/Agaricomycetes Cantharellales FIs *Sistotrema S. brinkmannii***

Otu0993 -24 3.E-12 Mu/Endogonomycetes unidentified unidentified

^________________________________________________________________________________________________________________________________________________________________________________________________________^

clay

C vs BA Otu1040 23.3 2.E-10 Ba/Agaricomycetes unidentified unidentified

Otu1768 7.8 8.E-06 As/Sordariomycetes unidentified unidentified

Otu2596 4.6 3.E-04 As/Leotiomycetes Vibrisseaceae *Phialocephala P. humicola*

Otu2087 3.1 2.E-04 As/Sordariomycetes Plectosphaerellaceae *Plectosphaerella P. cucumerina*

Otu2215 -4.2 2.E-05 As/unidentified unidentified unidentified

**Otu1824 -5.7 2.E-07 As/Sordariomycetes Annulatascaceae *Pseudoproboscispora***

**Otu1711 -6.3 7.E-27 As/Sordariomycetes Halosphaeriaceae *Cirrenalia C. iberica***

**Otu2238 -6.6 3.E-05 As/Sordariomycetes Nectriaceae *Fusicolla F. aquaeductuum***

**Otu0763 -6.7 3.E-04 Gl/Glomeromycetes Glomeraceae *Funneliformis F. caledonium***

**Otu1706 -9.6 6.E-05 As/Sordariomycetes Halosphaeriaceae *Natantispora N. retorquens***

**Otu2147 -11.5 3.E-05 As/Sordariomycetes Nectriaceae *Fusarium F. solani***

**Otu1781 -14.1 3.E-12 As/Orbiliomycetes Orbiliaceae *Arthrobotrys A. oligospora***

Otu2498 -22.0 9.E-09 As/Sordariomycetes unidentified unidentified Sordariomycetes sp

^________________________________________________________________________________________________________________________________________________________________________________________________________^

silt

C vs BA Otu1711 3.1 4.E-05 As/Sordariomycetes unidentified unidentified

**Otu2147 -4.1 4.E-07 As/Sordariomycetes Annulatascaceae *Pseudoproboscispora***

**Otu1781 -6.2 1.E-17 As/Sordariomycetes Halosphaeriaceae *Cirrenalia C. iberica***

**Otu2113 -7.9 2.E-24 As/Orbiliomycetes Orbiliaceae *Arthrobotrys A. oligospora***

**Otu0777 -8.2 8.E-11 As/Sordariomycetes Nectriaceae *Fusarium F. solani***

Otu1824 -23.4 4.E-10 Mu/Endogonomycetes unidentified unidentified

^________________________________________________________________________________________________________________________________________________________________________________________________________^

clay

C vs BHA **Otu1732 13.3 4.E-09 Mu/Mucoromycetes Rhizopodaceae *Rhizopus R. arrhizus***

**Otu1768 8.5 1.E-07 As/Sordariomycetes unidentified unidentified**

**Otu2596 5.6 4.E-08 As/Leotiomycetes Vibrisseaceae *Phialocephala P. humicola***

Otu2087 2.4 2.E-05 As/Sordariomycetes Plectosphaerellaceae *Plectosphaerella P. cucumerina*

**Otu1824 -4.8 3.E-04 As/Sordariomycetes Annulatascaceae Pseudoproboscispora**

**Otu1711 -5.4 3.E-12 As/Sordariomycetes Halosphaeriaceae *Cirrenalia C. iberica***

**Otu0763 -6.9 1.E-04 Gl/Glomeromycetes Glomeraceae *Funneliformis F. caledonium***

**Otu1706 -8.1 2.E-05 As/Sordariomycetes Halosphaeriaceae *Natantispora N. retorquens***

**Otu2147 -8.6 1.E-09 As/Sordariomycetes Nectriaceae *Fusarium F. solani***

**Otu1781 -12.0 3.E-09 As/Orbiliomycetes Orbiliaceae *Arthrobotrys A. oligospora***

^________________________________________________________________________________________________________________________________________________________________________________________________________^

silt

C vs BHA **Otu1732 27.2 5.E-14 Mu/Mucoromycetes Rhizopodaceae *Rhizopus R. arrhizus***

**Otu2012 9.2 9.E-05 As/Sordariomycetes Hypocreaceae *Trichoderma T. hamatum***

Otu2993 -1.9 7.E-05 Ro/Rozellomycotina unidentified unidentified

Otu1803 -2.6 5.E-05 unidentified unidentified unidentified

**Otu1711 -4.4 4.E-07 As/Sordariomycetes Halosphaeriaceae *Cirrenalia C. iberica***

**Otu2238 -5.4 4.E-04 As/Sordariomycetes Nectriaceae *Fusicolla F. aquaeductuum***

**Otu1781 -7.1 8.E-14 As/Orbiliomycetes Orbiliaceae *Arthrobotrys A. oligospora***

**Otu2147 -8.6 5.E-05 As/Sordariomycetes Nectriaceae *Fusarium F. solani***

^________________________________________________________________________________________________________________________________________________________________________________________________________^

**Figure S1.** Duration (number of months), simulated growing period by month with temperature conditions along with procedures and time of samplings indicated as arrows conducted in the microcosm experiment.


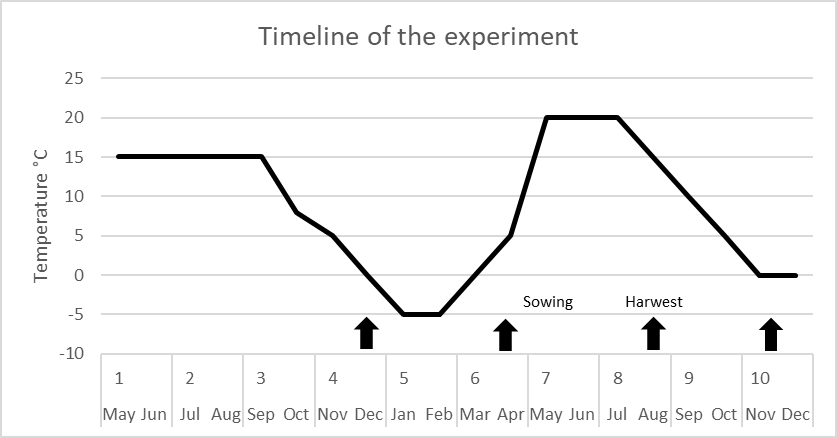

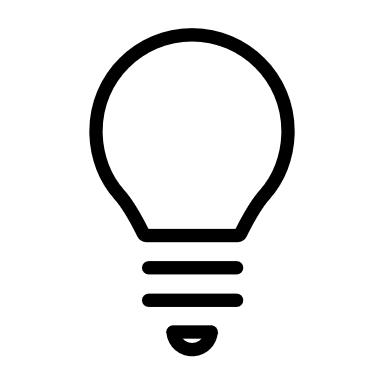


**Figure S2.** Shoot biomass per gram of dry mass after the harvest (3^rd^ sampling) in microcosms. Asterisks show the differences in microcosms in clay and silt soil between controls (C) and four different bark-derived organic soil amendments (B, BH, BA, BHA) determined with ANOVA and t-test at *p* > 0.05. The data for samples from pine and spruce-bark derived amendments are combined (36 microcosm samples for both clay and silt soil).

**
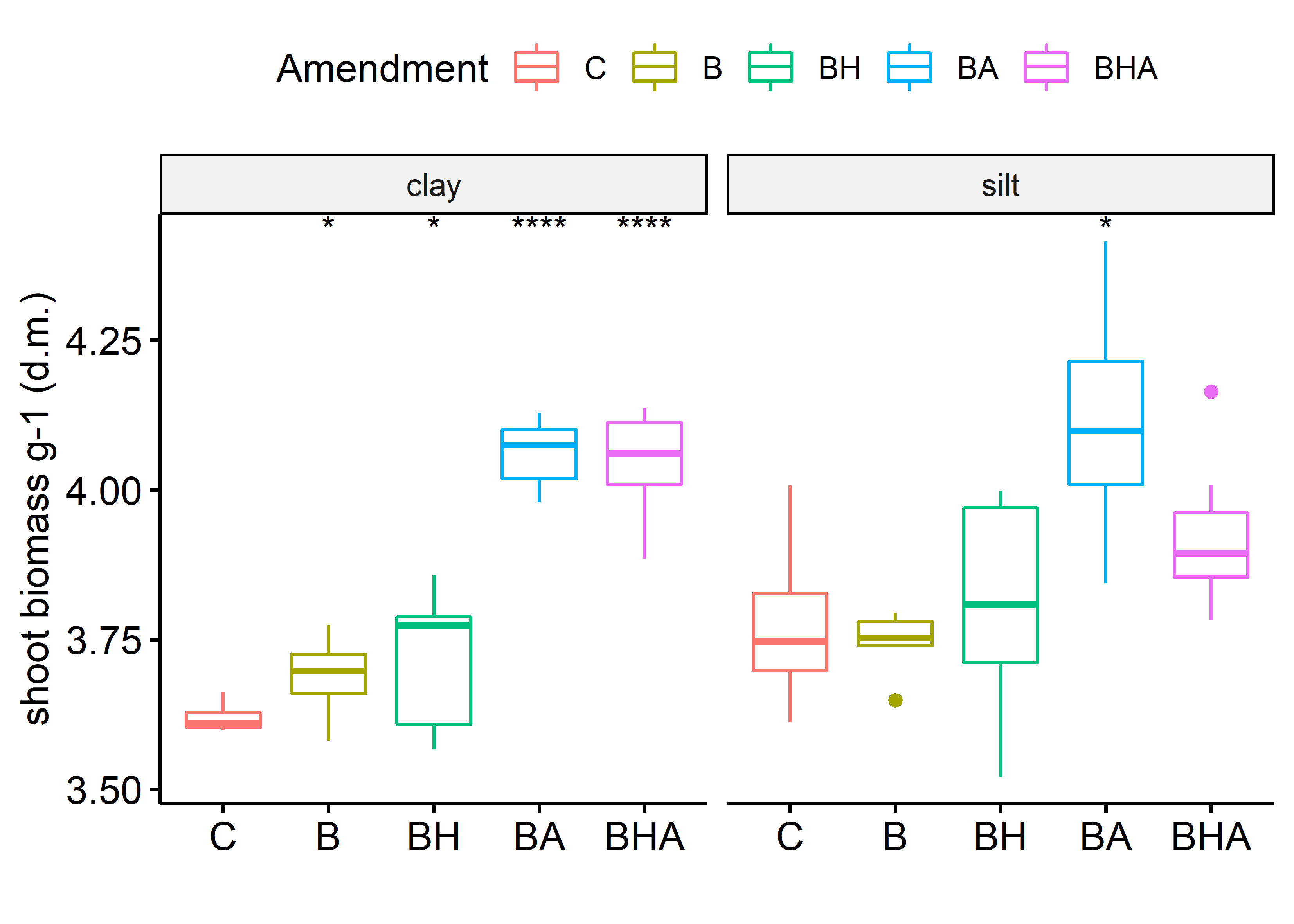
**
